# Multi-scale computational modeling towards efficacy in radiopharmaceutical therapies while minimizing side effects: Modeling of amino acid infusion

**DOI:** 10.1371/journal.pcbi.1013247

**Published:** 2025-07-16

**Authors:** Aryan Golzaryan, Mohammad Souri, Farshad M. Kashkooli, Arman Rahmim, M. Soltani

**Affiliations:** 1 Department of Mechanical Engineering, K. N. Toosi University of Technology, Tehran, Iran; 2 Department of Integrative Oncology, BC Cancer Research Institute, Vancouver, British Columbia, Canada; 3 Departments of Radiology and Physics, University of British Columbia, Vancouver, British Columbia, Canada; 4 Department of Electrical and Computer Engineering, University of Waterloo, Waterloo, Ontario, Canada; 5 Centre for Biotechnology and Bioengineering (CBB), University of Waterloo, Waterloo, Ontario, Canada; 6 Centre for Sustainable Business, International Business University, Toronto, Ontario, Canada; 7 Balsillie School Fellow at the Balsillie School of International Affairs (BSIA), Waterloo, Ontario, Canada; University at Buffalo - The State University of New York, UNITED STATES OF AMERICA

## Abstract

Amino acid infusion (AAI) is a technique used in radiopharmaceutical therapy (RPT) to reduce toxicity in kidney and increase clearance rate of radiopharmaceuticals from body. In this study our aim is to evaluate its effect in personalized RPT considering kidney and salivary glands as dose limiting organs using a multiscale modeling framework. We developed a Physiologically-Based Pharmacokinetic (PBPK) model consisting of 19 compartments, personalized it for four prostate cancer patients using data derived from gamma camera imaging. This model was used to investigate the influence of AAI on the absorbed dose to tumors and organs at risk. We then computed the maximum safe injected activity based on the PBPK model. To address the effects of interstitial fluid pressure (IFP) and tumor heterogeneity, we coupled the PBPK model with convection-diffusion-reaction (CDR) equations. To compare the effectiveness of our modeling approaches, we calculated absorbed doses to the tumors with and without AAI, using both the standalone PBPK model and the coupled PBPK-CDR model. Our findings revealed a relative error (RE) of 9.6% ± 2.2% (mean ± SD) in total tumor absorbed dose calculation between PBPK and CDR equations, attributable to the consideration of IFP. Moreover, AAI proved beneficial for RPT when the kidney was designated as the organ-at-risk. It enabled an increase in radiopharmaceutical injection from 12.3 ± 6.32 MBq (mean ± SD) to 15.45 ± 6.95 MBq (RE: 28.5% ± 15.7%), resulting in a corresponding increase in tumor absorbed dose from 67.8 ± 47.45 Gy to 72.43 ± 51.03 Gy (RE: 8.6% ± 5.4%), while maintaining critical kidney absorbed dose limits. However, this was not observed when the salivary gland was considered the dose-limiting organ. Although, AAI allowed for increased therapeutic injection ranging from 4.22 ± 2.23 MBq to 5.25 ± 3.14 MBq (RE: 19.2% ± 9.9%), it results in a minimal increase in tumor absorbed dose of 0.22 ± 0.04 (RE: 1.4% ± 1.3%). Statistical analysis using the Wilcoxon Signed-Rank Test revealed significant effects of AAI on administered activity and tumor absorbed dose (p-value = 0.007 < 0.05). Finally, a local sensitivity analysis was performed on selected radiation and tumor transportation parameters individually to evaluate their impact on the tumor absorbed dose. In conclusion, selection of organ-at-risk in personalized RPT is critical, as it determines the injected activity amount and the efficacy of delivery-enhancing techniques.

## Author summary

Radiopharmaceutical therapy (RPT) is emerging as a highly efficient approach to treating cancer due to its precise and potent targeting of cancerous cells. However, several issues require attention. The need to determine the appropriate amount of injected therapeutics to prevent both over and under-treatment; and the ongoing effort to enhance the efficiency of radiopharmaceutical delivery. Mathematical models serve as potent instruments for simulating the distribution of radiopharmaceuticals. In the current study, we concentrate on a crucial aspect of RPT that, to our knowledge, has not been previously examined through the use of advanced modeling techniques. It is very important to enable higher rate of delivered dose to tumors while reducing side effects in RPT. To address this issue, the present study assesses the impact of amino acid administration on precise RPT for four patients using a multi-scale modeling framework. We have developed a multiscale mathematical framework to investigate the influence of amino acid administration on the absorbed dose to tumors and organs at risk. We then computed the maximum safe injected activity and discussed the importance of identifying organs at risk for each patient, as these organs determine the maximum amount of injected radioactivity that is safe.

## 1. Introduction

Cancer continues to pose significant threats to human health worldwide, with its complex and diverse nature, challenging conventional treatment approaches [1–3]. Over the years, advancements in medical science have led to the development of various treatment options ranging from surgery and chemotherapy to immunotherapy and targeted therapy [4]. Among these, radiopharmaceutical therapy (RPT) has emerged as a promising novel approach that utilizes the power of radioactive isotopes to selectively target and destroy cancer cells while minimizing damage to healthy tissues [5].

In recent years, efforts have been made to enhance the efficacy and safety of RPT through the implementation of innovative techniques. These include methods such as salivary gland cooling [6], the metronomic approach [7,8], and amino acid administration (AAI) [9,10], which aim to optimize treatment outcomes by reducing adverse effects and enhancing tumor targeting. However, the full potential of these techniques requires further analysis and investigation to determine their effectiveness in clinical settings. Nevertheless, the concept of personalized RPT has gained traction, wherein treatment strategies are tailored to individual patients based on their unique characteristics and medical history [11–14].

Physiologically based pharmacokinetic (PBPK) and pharmacokinetic-pharmacodynamic (PK-PD) models are advanced tools that utilize mathematical algorithms to simulate drug concentrations in various tissues and organs over time, as well as their resulting effects [15–19]. PBPK modeling has significantly advanced in recent years, enhancing its capability to assess absorption, distribution, metabolism, and excretion (ADME) properties, especially in the rapidly evolving field of radiopharmaceuticals [20–23] and pharmaceutical nanoparticles [24]. These models effectively facilitate the extrapolation of drug pharmacokinetics from animal studies to human applications [25], as demonstrated by accurately predicting levofloxacin tissue penetration in intra-abdominal infections, which underscores their vital role in anti-infective PK-PD analyses [26]. Comprehensive systematic reviews have further validated PBPK models’ robust predictive accuracy throughout oncology drug development [27]. Integration of PBPK models with pharmacodynamic models, such as those used in cisplatin-based intraoperative intraperitoneal chemotherapy, exemplifies their practical clinical utility, enabling dose optimization and toxicity reduction [28,29]. Specific applications, such as a whole-body PBPK model for propranolol dosing in liver cirrhosis patients, further illustrate the tailored applicability of these models to distinct patient populations [30]. PBPK models have also become instrumental in predicting drug-drug interactions (DDIs), through the simulation of metabolic pathway interactions [31,32]. Expanding their utility further, PBPK models are increasingly applied in specialized patient populations, including pediatric patients and those undergoing treatments for acute lymphoblastic leukemia [33,34]. Enhanced by machine learning integration, PBPK models continue to refine predictive accuracy across diverse demographic groups [35]. Additionally, the application of these models in targeted therapies, including antibody-drug conjugates, addresses the intricate PK-PD relationships unique to these complex agents, facilitating more precise dosing strategies for individual patient needs [36].

Despite their substantial advantages, PBPK models present both opportunities and challenges. Mechanistically, they provide physiologically relevant predictions that support individualized dosing and enable extrapolations across species and modalities [37–39]. PBPK models are particularly valuable for predicting tissue-specific and tumor-specific pharmacokinetics. However, their inherent complexity often necessitates extensive parameterization and large volumes of input data, which can hinder parameter optimization and limit practical feasibility [40]. As a result, researchers frequently adjust the level of organ-specific detail according to the modeling objective and therapeutic context, simplifying the model structure where appropriate. Most whole-body PBPK models represent organs and tissues as compartments interconnected by arterial and venous blood flows [41]. A widely accepted structural framework—also adopted in our approach—consists of interconnected compartments corresponding to individual organs and tissues [28,29]. These are linked through arterial and venous blood pools that constitute the systemic circulation, responsible for distributing substances throughout the body. Typically, blood flows from arteries to veins; however, the lung compartment represents an exception, where flow occurs from veins to arteries [42–47]. This compartment incorporates both the pulmonary circulation, which facilitates gas exchange, and the heart functioning as a pump (though it is not modeled as a distinct organ) [41,46–48]. Instead, the heart compartment accounts for myocardial tissue and its associated capillaries [41,46–48].

While this standardized structure provides a robust foundation, variations in organ representation and connectivity are often introduced based on the specific goals of the model and the pharmacokinetic properties of the drug under investigation. Accordingly, PBPK model complexity and predictive accuracy vary depending on the intended application and target organs. The level of detail may range from simplified models with a few lumped tissue groups to comprehensive full-body models featuring numerous distinct compartments, each with its own blood flow rate [49,50]. In some cases, tissues with similar perfusion characteristics or drug transit times are consolidated into single compartments to reduce computational demands. Modular PBPK frameworks also allow flexible adaptation of blood flow structures to accommodate different physiological scenarios or population groups [51].

PBPK modeling also serves as a key tools in advancing personalized RPT [52]. PBPK modeling utilizes mathematical algorithms to simulate the absorption, distribution, metabolism, and excretion of drugs within the body, allowing for the prediction of drug concentrations in various tissues and organs over time [20–23]. By incorporating patient-specific data such as physiological parameters and tumor characteristics, PBPK modeling enables clinicians to optimize treatment regimens and minimize potential side effects [42–45]. While PBPK modeling offers numerous advantages in personalized RPT, such as its ability to account for inter-individual variability and predict drug behavior under different conditions, it is limited by the fact that it commonly involves ordinary differential equations (ODEs) with respect to time only, not enabling spatial coupling [53,54]. To address this limitation, convection-diffusion-reaction (CDR) equations have been proposed as spatiotemporal modeling approaches, invoking partial differential equations (PDEs) with respect to both time and space, that enable a more comprehensive modeling of drug distributions within tumors [55,56]. Additionally, advancements in microvascular modeling, such as those proposed, hold promise for further enhancing the accuracy and reliability of RPT simulation by providing detailed insights into the distribution of radioactive isotopes within the tumor microenvironment [57,58].

Understanding tumor progression from angiogenesis to advanced growth stages necessitates robust modeling frameworks that can capture the complex spatiotemporal dynamics of cancer biology [59,60]. Angiogenesis, the formation of new blood vessels from pre-existing vasculature, is a pivotal process that supports tumor expansion [60,61]. A systems biology perspective has contributed significantly to deciphering the regulatory mechanisms behind vascular growth and remodeling, integrating signaling pathways, cellular behavior, and tissue-level responses [62]. Mathematical models—ranging from simple compartmental representations to spatially resolved systems—have been instrumental in capturing these dynamics and linking them to experimental observations [63,64]. Two-dimensional (2D) and three-dimensional (3D) models are both widely used in tumor modeling, each serving distinct research objectives and computational constraints [65,66]. 2D models are commonly employed due to their computational efficiency and suitability for studying processes such as tumor cell proliferation, nutrient diffusion, and angiogenesis within simplified geometries [1–3,67,68]. These models are particularly useful for performing extensive parameter sweeps or integrating with agent-based and continuum frameworks [69]. In contrast, 3D models offer the advantage of integrating with advanced in vitro systems such as microfluidic platforms, where experimental imaging data can be coupled with hybrid multiscale models to investigate vascularization dynamics and cellular organization in more realistic environments [70]. Nevertheless, their increased biological detail comes at a higher computational cost. Modeling the tumor microenvironment—an intricate network of stromal cells, extracellular matrix, and signaling gradients—requires capturing not only spatial organization but also dynamic interactions that regulate tumor invasion and growth [71]. The selection between static (time-invariant) and dynamic (time-evolving) frameworks further distinguishes models based on whether they capture snapshots or progression of the tumor microenvironment.

The tumor microenvironment (TME) plays a critical role in modulating tumor behavior, therapeutic resistance, and overall treatment efficacy. It encompasses a heterogeneous and evolving milieu, including stromal cells, extracellular matrix (ECM), blood and lymphatic vessels, oxygen and pH gradients, and immune cell infiltrates [72]. Dynamic modeling of the TME enables simulation of how gradients of oxygen and nutrients develop and change over time, influencing tumor proliferation, migration, and necrosis. These models are particularly valuable for studying drug delivery, which is heavily affected by irregular and leaky tumor vasculature, elevated interstitial fluid pressure, and heterogeneous permeability [73]. Transport processes such as diffusion, convection, and cellular uptake are commonly modeled using PDEs, often coupled with cell-scale or tissue-scale dynamics [59]. Mechanically coupled models, such as reaction–diffusion frameworks, have been used to predict tumor response to therapies like neoadjuvant chemotherapy in breast cancer [74], while MRI-informed models have captured glioma growth alongside mass effects induced by tumor expansion [75]. Image-based, patient-specific fluid dynamics modeling has further enhanced the ability to optimize treatment strategies tailored to individual tumor physiology [76]. In addition, simulation approaches have been employed to assess the effectiveness of advanced therapeutic modalities, including gold nanoparticle-assisted microwave-induced hyperthermia, in improving tumor control [77].

To bridge scales and incorporate biological complexity, hybrid models have emerged as a powerful framework in computational oncology [78]. These models combine discrete elements—such as individual cells, blood vessels, or drug particles—with continuous fields like oxygen, growth factors, and therapeutic agents to simulate multiscale interactions across biological hierarchies. Hybrid modeling is particularly well-suited for capturing the coupling between tumor growth, angiogenesis, immune responses [79], and drug transport [80–83]. Agent-based models are often employed to represent the stochastic behavior of individual cells, while PDEs or stochastic differential equations describe the diffusion and convection of extracellular signals and therapeutic agents [84]. Recent studies have demonstrated the effectiveness of these models when calibrated against experimental data, such as Bayesian approaches used to predict in vitro tumor growth from agent-based simulations [85]. Experimental platforms, including 3D tumor spheroids and microfluidic systems, offer valuable datasets for validating such models. For instance, mathematical modeling of tumor spheroids has revealed dynamic cellular behaviors like proliferation and necrosis influenced by diffusion-limited nutrient availability [86], while 3D cultures have illuminated how self-generated gradients and adhesion dynamics guide active cell migration [87]. Integration of experimental and computational methods is especially powerful in quantifying cancer progression and morphology in controlled 3D environments, as shown in multiscale studies that bridge microscopy data with model-based predictions [88,89]. The integration of tumor metabolic processes with spatially-resolved multiscale models has clarified how metabolism drives tumor growth and metastasis, enhancing the development of targeted treatment strategies [90,91]. As the field progresses, hybrid modeling frameworks are increasingly incorporating experimental data streams, machine learning tools, and patient-specific parameters, thereby moving toward predictive and personalized simulations in cancer research [92].

In any case, PBPK and CDR modeling have the potential to provide significant understanding of various phenomena in RPT. In our own recent efforts, we have recently modeled and investigated non-linearities, multi-bolus injections as well as albumin binding [93], and developed a novel spatiotemporal model to examine how various physiological parameters affect the delivery of RPT, using ^177^Lu-labeled Prostate-specific membrane antigen (PSMA), taking into account the complex tumor microenvironment [55,94]. Additionally, we have designed a framework to identify the optimal injection profile, employing metronomic injection strategies to maximize the dose delivered to the tumor while minimizing the amount of therapeutics administered [8].

In the current study, we concentrate on a crucial aspect of RPT that, to our knowledge, has not been previously examined through the use of advanced modeling techniques. We have developed a multiscale framework by integrating a PBPK model with the CDR equation. It is very important to enable higher rate of delivered dose to tumors while reducing side effects in RPT. To address this issue, the present study assesses the impact of AAI on precise RPT for four patients using a multi-scale modeling framework. In clinical practice, amino acids are administered 30 minutes before drug injection, resulting in an increase in the clearance rate of radiopharmaceuticals; this approach has been previously investigated using PBPK models to assess its impact on toxicity [20–23,42–45,52,95–98]. A whole-body scale mathematical analysis is conducted to assess the impact of amino acid infusion on the circulation of radiopharmaceuticals via a PBPK modeling approach. Additionally, the study aims to increase the accuracy of radiopharmaceutical distribution in the tumor by examining the effect of the tumor microenvironment. Therefore, by modeling the angiogenesis process from two parent vessels using a system of PDEs, microvessels around and inside the tumor are simulated, and the resulting geometry is utilized to couple PBPK equations with CDR equations. Using personalized RPT, the kidney and salivary gland are assumed as organs-at-risk, each separately, in order to predict the influence of AAI on the efficacy of treatment. Finally, the Wilcoxon Signed-Rank Test was conducted to assess the statistical significance of the effect of AAI on the tumor absorbed dose, as well as the significance of results derived from the PBPK model compared to the coupled PBPK-CDR model. Additionally, a local sensitivity analysis was performed on selected radiation and tumor transportation parameters individually to evaluate their impact on the tumor absorbed dose.

## 2. Methods

**Fig 1:**
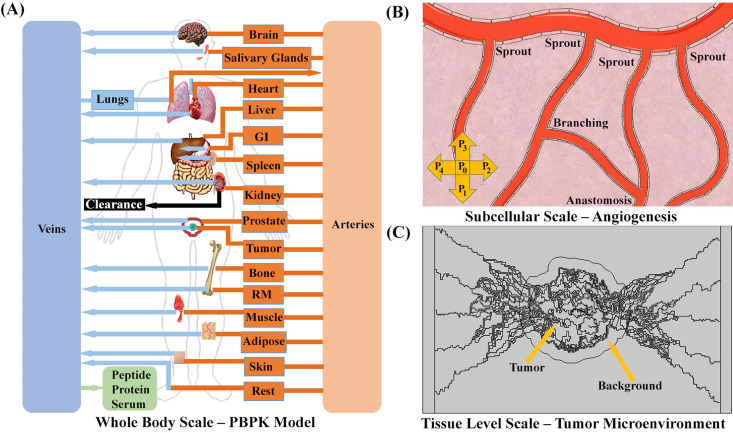
Illustration of the strategy. **(A)** A whole-body physiologically based pharmacokinetic (PBPK) model, consisting of 19 compartments, including normal organs and a tumor (prostate cancer), was developed. This model calculates absorbed doses in all orans including tumor and organs-at risk, and determines drug concentration within vascular space of tumor. In this framework, oxygenated blood enters the arterial compartment, from which it is distributed to all tissue compartments based on organ-specific blood flow rates. Blood exiting the tissues flows into the venous compartment and is routed back to the lungs for reoxygenation, thereby completing the circulatory loop. The systemic circulation is simplified into two main compartments—arterial and venous blood—with oxygenated blood from the lungs entering the arterial side directly, bypassing explicit modeling of the heart’s pumping function. Image from Pixabay (https://www.pixabay.com), used under the Pixabay License. **(B)** A schematic of capillary growth driven by the movement of individual tip endothelial cells (tECs). The migration of each cell, located at the nodes of a computational lattice (finite difference discretization), is governed by five states. These states are determined by coefficients representing the probabilities of the cell remaining stationary (P_0_) or moving in one of four directions: right (P_1_), up (P_2_), left (P_3_) or down (P_4_). The processes of anastomosis and branching occur as part of angiogenesis. Tumor induced angiogenesis creates a nonhomogeneous microvessels distribution within the tumor and it defines as tumor vascular space. Image created using Servier Medical Art (https://smart.servier.com/), licensed under CC BY 4.0. **(C)** The computational domain designed to simulate the spatiotemporal distribution of radiopharmaceuticals, encompassing parent vessels, tumor microvasculature, tumor interstitial space, and surrounding normal host tissue. Using the drug concentration in the tumor’s vascular space, derived from PBPK model, and the synthesized microvasculature, the CDR equations simulate spatiotemporal distribution of radiopharmaceuticals in order to calculate delivered dose to tumor more accurately. More information about mathematical modeling of angiogenesis process and PBPK model provided in S1 Text.

In this study, we construct a synthetic model of tumor microvasculature using mathematical modeling to simulate the angiogenesis process. Subsequently, we investigate fluid dynamics within microvessels and the extracellular spaces of simulated tumor microvasculature, aiming to analyze the impact of convective processes on the transportation and distribution of therapeutic agents. The analysis then shifts to examining the concentration of radiopharmaceuticals within organs-at-risk and their distribution in tumor vasculature through PBPK modeling. Data from four patients [42] are utilized to personalize the PBPK model and explore the effects of AAI on the concentration of radiopharmaceuticals in tumor microvasculature. Finally, the concentration of radiopharmaceuticals in the vascular space of the tumor is used as input for the CDR equation to simulate the spatiotemporal distribution in the tumor. Fig 1 provides a graphical representation of the study’s framework.

In the human body, oxygenated blood leaves the lungs and first reaches the left side of the heart, where it is then pumped into the arterial circulation to be delivered to various organs. Explicitly modeling every detail of this cardiac pumping mechanism is highly complex and often unnecessary for pharmacokinetic modeling. Therefore, in this study, we employ a widely accepted PBPK framework [8,20–23,28,46,47], in which the systemic circulation is simplified into two primary compartments: arterial blood and venous blood (Fig 1A) [41–45,48]. Within this PBPK framework, the heart’s physiological pumping function is not explicitly represented. Instead, oxygenated blood from the lungs is assumed to directly enter the arterial compartment, which then serves as the main conduit for distribution to the tissues [8,20–23,28,46,47]. The heart itself is modeled as a standard tissue compartment—similar to other organs—to capture its potential for peptide uptake, with defined vascular and interstitial sub-compartments. This abstraction allows for a computationally efficient yet physiologically meaningful simulation of whole-body peptide kinetics. From the arterial blood, perfusion into individual tissue compartments occurs, each characterized by specific blood flow rates, volumes, and drug or tracer uptake dynamics. Subsequently, blood exiting these tissues flows into the venous compartment, ultimately returning to the lungs for reoxygenation, thereby completing the circulatory loop [8,20–23,28,46,47].

### 2.1. Governing equation

#### 2.1.1. Convection-diffusion-reaction equation.

To comprehensively capture the distribution of radiopharmaceuticals within tumors and assess their impact, this organ is divided into five distinct compartments: vascular space, interstitial space, receptors on the cell membrane, and intracellular space. Due to the emission range of ^177^Lu, a segment of muscle is also included, where the presence of radiopharmaceuticals could potentially influence the tumor. The equation governing the concentration of labelled ($C_{int}^{*}$) and unlabeled peptides ($C_{int}$) in IS is expressed as follows [99,100], with the associated parameters summarized in Table 1:

**Table 1:** Parameters considered in CDR equation for simulating spatiotemporal distribution in tumor microenvironment.

Variable	Expression	Value	Reference
$D_{PSMA}$	Diffusivity of PSMA ligand	$8.7\times 10^{-7}\;[\frac{{cm}^{2}}{s}]$	[101]
$k_{on}$	Association Rate of Ligands to Receptors	$0.046\;[\frac{liter}{nmol\times min}]$	[102]
$k_{off}$	Disassociation Rate of Ligands from Receptors	$0.046\;[\frac{1}{\min}]$	[102,103]
$\lambda_{phy}$	Physical Decay of ^177^Lu	$7.15\times 10^{-5}\;[\frac{1}{\min}]$	–
R	Receptor Density	Estimated – Table 2	–
P	Vessel wall permeability	$3.3\times 10^{-4}\;[\frac{cm}{s}]$	[104,105]
$\sigma_{f}$	Coefficient of filtration reflection	0.9	[106]
$k_{int}$	Internalization Rate of Radiopharmaceutical	0.001$\;[\frac{1}{\min}]$	[43]
$\lambda_{\deg}$	Release Rate of Radiopharmaceutical	Estimated – Table 2	–


$$\underset{\begin{array}{c} Labelled\;Peptides\;\\ Concentration\;\\ in\;interstitial\;space \end{array}}{\underbrace{\frac{\partial C_{int}^{*}}{\partial t}}}=\underset{\begin{array}{c} Diffusion\;\\ Mechanism \end{array}}{\underbrace{D_{PSMA}\nabla^{2}C_{int}^{*}}}-\underset{\begin{array}{c} Convection\;\\ Mechanism \end{array}}{\underbrace{v_{i}\nabla C_{int}^{*}}}+\underset{\begin{array}{c} Disassociation\\ Rate\;from\;\\ Receptors \end{array}}{\underbrace{k_{off}C_{int}^{*}}}-\underset{\begin{array}{c} physical\\ decay \end{array}}{\underbrace{\lambda_{phy}C_{int}^{*}}}\;\;+\underset{Binding\;to\;Receptors}{\underbrace{\left(R-C_{bound}^{*}-C_{bound}\right)k_{on}C_{int}^{*}}}+\;(\underset{\begin{array}{c} Source\;Term\\ \left(Capillaries\right) \end{array}}{\underbrace{\phi_{B}^{*}}}-\underset{\begin{array}{c} Sink\;Term\\ \left(\begin{array}{c} Lymphatic\;\\ Vessles \end{array}\right) \end{array}}{\underbrace{\phi_{L}^{*}}})$$ (1)

$$\underset{\begin{array}{c} Unlabelled\;Peptides\;\\ Concentration\;\\ in\;interstitial\;space \end{array}}{\underbrace{\frac{\partial C_{int}}{\partial t}}}=\underset{\begin{array}{c} Diffusion\;\\ Mechanism \end{array}}{\underbrace{D_{PSMA}\nabla^{2}C_{int}}}-\underset{\begin{array}{c} Convection\;\\ Mechanism \end{array}}{\underbrace{v_{i}\nabla C_{int}}}+\underset{\begin{array}{c} Disassociation\\ Rate\;from\;\\ Receptors \end{array}}{\underbrace{k_{off}C_{int}}}+\underset{\begin{array}{c} physical\\ decay \end{array}}{\underbrace{\lambda_{phy}C_{int}^{*}}}\;\;+\underset{Binding\;to\;Receptors}{\underbrace{\left(R-C_{bound}^{*}-C_{bound}\right)k_{on}C_{int}}}\;+\;(\underset{\begin{array}{c} Source\;Term\\ \left(Capillaries\right) \end{array}}{\underbrace{\phi_{B}}}-\underset{\begin{array}{c} Sink\;Term\\ \left(\begin{array}{c} Lymphatic\;\\ Vessles \end{array}\right) \end{array}}{\underbrace{\phi_{L}}})$$ (2)

In equation 1 and 2, $D_{PSMA}$ represents the diffusivity of PSMA ligand within the interstitial fluid, which is dependent on the physicochemical characteristics of the radiopharmaceutical. The disassociation term incorporates $k_{off}$, which represents the unbinding of peptides from receptors and entering to interstitial space. The parameter $\lambda_{phy}$ denotes the physical decay of ^177^Lu (also known as natural decay of ^177^Lu) and both the term which contains this parameter and the competition between labelled and unlabeled peptides which indicated in fifth term couples system of labelled and unlabeled equations together. Finally, transvascular transport of radiopharmaceuticals into extracellular space (source) and their drainage from interstitial space by lymphatic system (sink) calculated as follows:

Source Term (microvessels):


$$\phi_{B}^{*}=\Psi_{B}\left(1-\sigma_{f}\right)C_{v}^{*}+\frac{PS}{V}(C_{v}^{*}-C_{int}^{*})\frac{Pe}{e^{Pe}-1}$$ (3)

$$\phi_{B}=\Psi_{B}\left(1-\sigma_{f}\right)C_{v}+\frac{PS}{V}(C_{v}-C_{int})\frac{Pe}{e^{Pe}-1}$$ (4)

Where $\sigma_{f}$ is the coefficient of reflection, P is capillary permeability, and $\frac{S}{V}$ is surface area per unit volume. Pe is the trans-capillary Peclet number which defined as $Pe=\;\frac{\Psi_{B}\left(1-\sigma_{f}\right)V}{PS}$.

Sink term (Lymphatic vessels):


$$\phi_{L}^{*}=\Psi_{L}C_{v}^{*}$$ (5)

$$\phi_{L}=\Psi_{L}C_{v}$$ (6)

Concentration in vascular space as second sub-compartment of tumor denoted by $C_{v}^{*}$ and$\;C_{v}$, determined using whole-body PBPK model. The transport of radiopharmaceuticals between vascular and interstitial vascular space due to concentration difference and convection through vessel pores modeled using equations (6–9).

In this investigation, laminar flow characteristics are assumed for the blood flow in the microvascular network [106]. Determination of intravascular pressure and velocity relies on the pressure in the parent vessels. The microvascular walls are considered leaky, permitting the coupling of vascular space blood pressure with interstitial fluid pressure (IFP) according to Starling’s law. The condition of the microvascular walls is assessed as per previous studies [107,108].


$$v_{t}=L_{p}\left(P_{B}-P_{i}-\sigma \left(\pi_{b}-\pi_{i}\right)\right)$$ (7)

Equation 7 represents the transvascular fluid velocity from the walls, denoted as $\;v_{t}$. Within this equation, the hydraulic conductivity of the capillary walls within the domain is denoted as $L_{p}=2.7\times 10^{-12}(m/Pa\cdot s)$ [107,108]. Additionally the intravascular blood pressure, designated as, $P_{B}$, the average osmotic reflection coefficient for plasma proteins, σ=0.82 [109–111], the osmotic pressure of blood $\pi_{b}=2666\;(Pa)$ [107,112], and the osmotic pressure of interstitial fluid $\pi_{i}=2000\;(Pa)$ [100] are included.

Biological tissues, characterized by their porous properties, necessitate the application of Darcy’s law to elucidate fluid flow phenomena within them. This law facilitates the characterization of fluid flow within the interstitial spaces, incorporating source and sink terms resulting from the activity of the microvascular and lymphatic drainage systems intertwined within the tissue. Mathematically, this can be expressed as follows [107,108].


$$v_{i}=-\kappa \nabla P_{i}$$ (8)

$$\nabla .v_{i}=\underset{\begin{array}{c} Source\;\\ Term \end{array}}{\underbrace{v_{t}}}-\underset{\begin{array}{c} Sink\;\\ Term \end{array}}{\underbrace{v_{l}}}$$ (9)

Equation 8 employs the symbol $v_{i}$ to represent the interstitial fluid velocity (IFV). The coefficient $\kappa =6.4\times 10^{-14}(m^{2})$ [113] denotes the interstitial hydraulic conductivity, defined mathematically as the ratio of tissue permeability to the dynamic viscosity of the fluid. Equation 9 introduces variable $v_{l}$, termed the sink term, indicating fluid remo val by the lymphatic system. However, due to the restricted functionality of the lymphatic system within tumors, it remains unaccounted for in the current investigation.

The third part of tumor which is considered is receptors on cell membrane. As prostate cancer cells overexpress PSMA receptors on their membrane, they are known as biomarker for prostate cancer patient and used as a target for ^177^Lu-PSMA radiopharmaceuticals. The binding of both labeled and unlabeled peptides captured using a nonlinear PSMA-specific association model, where both type of peptides compete with each other in order to bind to free receptors (R). By modeling this phenomenon, in addition to capturing physical decay, two system of equations (labeled and unlabeled peptides) are coupled together. After the association process, they either internalized into cancerous cells or they disassociate from receptors and enter to interstitial space again.


$$\underset{\begin{array}{c} Labelled\;Peptides\;\\ Concentration\;\\ in\;interstitial\;space \end{array}}{\underbrace{\frac{\partial C_{bound}^{*}}{\partial t}}}=\underset{Binding\;to\;Receptors}{\underbrace{\left(R-C_{bound}^{*}-C_{bound}\right)k_{on}C_{int}^{*}}}-\underset{\begin{array}{c} Disassociation\\ Rate\;from\;\\ Receptors \end{array}}{\underbrace{k_{off}C_{bound}^{*}}}\;\;-\underset{\begin{array}{c} physical\\ decay \end{array}}{\underbrace{\lambda_{phy}C_{bound}^{*}}}-\underset{\begin{array}{c} Internalization\;\\ Rate\;into\;cells \end{array}}{\underbrace{k_{int}C_{bound}^{*}}}$$ (10)

$$\underset{\begin{array}{c} Unlabelled\;Peptides\;\\ Concentration\;\\ in\;interstitial\;space \end{array}}{\underbrace{\frac{\partial C_{bound}}{\partial t}}}=\underset{Binding\;to\;Receptors}{\underbrace{\left(R-C_{bound}^{*}-C_{bound}\right)k_{on}C_{int}}}+\underset{\begin{array}{c} Disassociation\\ Rate\;from\;\\ Receptors \end{array}}{\underbrace{k_{off}C_{bound}}}\;\;+\underset{\begin{array}{c} physical\\ decay \end{array}}{\underbrace{\lambda_{phy}C_{bound}^{*}}}-\underset{\begin{array}{c} Internalization\;\\ Rate\;into\;cells \end{array}}{\underbrace{{\;k}_{int}C_{bound}}}$$ (11)

In line with several studies [8,20–23,42–45,52,55,95,97], we made the assumption that once peptides are internalized into the intracellular space, they cannot exit the cells and only diminish through biological degradation processes, with the degraded material being directly cleared from the body. This assumption is based on the consideration that the impact of active pumps expelling radiopharmaceuticals from cells is negligible, given the lack of detailed and consistent information about their specific role in radiopharmaceutical transport.


$$\underset{\begin{array}{c} Labelled\;Peptides\;\\ Concentration\;\\ in\;intracellular\;space \end{array}}{\underbrace{\frac{\partial C_{intern}^{*}}{\partial t}}}=\;\underset{\begin{array}{c} Internalization\\ Rate\;from\;\\ Receptors \end{array}}{\underbrace{k_{int}C_{intern}^{*}}}-\underset{\begin{array}{c} physical\\ decay \end{array}}{\underbrace{\lambda_{phy}C_{intern}^{*}}}-\underset{degradation}{\underbrace{\lambda_{\deg}C_{intern}^{*}}}$$ (12)

$$\underset{\begin{array}{c} Unlabelled\;Peptides\;\\ Concentration\;\\ in\;intracellular\;space \end{array}}{\underbrace{\frac{\partial C_{intern}}{\partial t}}}=\underset{\begin{array}{c} Internalization\\ Rate\;from\;\\ Receptors \end{array}}{\underbrace{k_{int}C_{bound}}}+\underset{\begin{array}{c} physical\\ decay \end{array}}{\underbrace{\lambda_{phy}C_{bound}^{*}}}-\underset{degradation}{\underbrace{\lambda_{\deg}C_{intern}}}$$ (13)

Moreover, a proportion of the amount of radiopharmaceuticals within muscle compartment considered in calculation of delivered dose to tumor. More detail about tumor vascular compartment and muscle provided in section 2.1.2.

#### 2.1.2. Physiologically based pharmacokinetic modeling.

Towards realistic calculation of the concentrations of radiopharmaceuticals in tumor microvessels, a PBPK model consist of 19 compartments was developed and used (See S1 Text for details). The model is personalized for 4 different metastatic castration-resistant prostate cancer (mCRPC) patients using data acquired by Kletting et. al [42]. The individual parameters were estimated by fitting our PBPK model to the extracted data via nonlinear regression and nonlinear least squares optimization to personalize the model (Tables 2 and C-F and Figs A-D in S2 Text). Then, the temporal density of peptides in arteries, veins, and 17 other organs throughout the treatment is calculated. PSMA-positive organs which are prostate, GI, spleen, liver, salivary gland, and tumor consist of 4 sub-compartments includes vascular space, interstitial space, receptors on the cell membrane, and intracellular space. Compartment of kidney which is other PSMA-positive organ designed by five distinct sub-compartments, in which in addition to four mentioned ones, has an unspecific binding element as well. The amount of radiopharmaceuticals in vascular space of tumor used as input to CDR equation in order to simulate the spatiotemporal distribution and also comparison of absorbed dose calculated by PBPK and CDR equations. Kidney with 8 Gy [114] and salivary glands with 7.5 Gy [42] absorbed dose tolerance considered as OAR in each treatment cycle.

**Table 2:** Measured and estimated parameters of 4 patients for PBPK Model.

Patient	Age [year]	BSA ${[m}^{2}]$	TER $\left[\frac{ml}{\min}\right]$	Measured Volume [ml]		Receptor Density $\left[\frac{nmol}{Liter}\right]$			Release Rate $\times 10^{-4}$ $\left[\frac{1}{\min}\right]$			Tumor Background [unity]	SG Perfusion Rate $\left[\frac{ml}{gr*min}\right]$
				SG	Kidney	Primary Tumor	Kidney	SG	Tumor	Kidney	SG		
P1	69	1.9	201	21	311	57	14	38	1.5	2.9	4.2	0.0064	0.074
P2	78	1.8	136	17	394	282	22	108	0.88	2.4	3.5	0.053	0.53
P3	54	2.1	252	52	268	52	24	41	2	2.3	3.3	0.0016	0.16
P4	53	2.0	176	29	296	136	46	80	2	3.1	4.8	0.018	0.16

The PBPK model provides a mechanistic framework to simulate the biodistribution of both labeled and unlabeled peptides throughout the body. In this model (Fig 1A), the body is subdivided into interconnected compartments that represent various tissues and organs. These compartments are linked primarily through blood flow, which is simplified into arterial and venous compartments. This structure enables the model to capture both the rapid distribution of the peptide via the vascular system and the subsequent exchange between blood and tissue compartments via transcapillary extravasation. Rather than modeling the heart as an explicit pump with complex dynamics, the model abstracts the circulatory system into two main compartments: the arterial and venous pools. Oxygenated blood exiting the lungs is assumed to flow directly into the arterial compartment. This approximation reflects a common approach in PBPK modeling [8,20–23,28,46,47], where the arterial compartment serves as the delivery vehicle for oxygenated blood and, in this context, for the peptide. By doing so, the model avoids unnecessary complexity while still capturing the essential role of blood flow in distributing the peptide throughout the body.

In our previously published PBPK model [8], the distribution of peptides is governed primarily by arterial delivery and venous return, which serve as the systemic pathways connecting all tissues. The arterial compartment equation describes the dynamic balance of peptide concentration in arterial blood.


$$\frac{d}{dt}P_{\;ART}=-\sum \frac{F_{i}}{V_{ART}}\cdot P_{\;i,v}+\frac{F}{V_{LU,v}}\cdot P_{\;LU,v}+\lambda_{phy}\cdot P_{ART}^{*}$$ (14)

$$\frac{d}{dt}P_{ART}^{*}=-\sum \frac{F_{i}}{V_{ART}}\cdot P_{\;i,v}+\frac{F}{V_{LU,v}}\cdot P_{\;LU,v}-\lambda_{phy}\cdot P_{ART}^{*}$$ (15)

In this equation, $P_{\;ART}$ and $P_{ART}^{*}$ represent the concentrations of unlabeled and labeled peptides, respectively, in the arterial compartment. $\frac{F_{i}}{V_{ART}}.P_{\;i,v}$ and $\frac{F_{i}}{V_{ART}}.P_{i,v}^{*}$ captures the outflow from arterial blood into the vascular spaces of all tissues, except the lungs. This term represents oxygenated arterial blood delivering peptide into tissues. The term $\frac{F}{V_{LU,v}}.P_{\;LU,v}$ and $\frac{F}{V_{LU,v}}.P_{LU,v}^{*}$ indicates the inflow of oxygenated blood (carrying peptide) from the lung’s vascular compartment into the arterial compartment. The radioactive decay rate $\lambda_{phy}$ accounts for the physical decay of labeled peptide.

The venous compartment equation captures the return of blood from various organs back toward the lungs.


$$\frac{d}{dt}P_{\;VEN}=-k_{\mathrm{Pr}}\cdot P_{\;VEN}+\sum (\frac{F_{i}}{V_{i}}P_{\;i,v}-\frac{F_{M}}{V_{M}}P_{\;M,v}-\frac{F_{GI}}{V_{GI}}P_{\;GI,v})+\frac{F_{S}+F_{GI}}{V_{L}}P_{\;L,v}+\lambda_{phy}\cdot P_{\;VEN}^{*}$$ (16)

$$\frac{d}{dt}P_{VEN}^{*}=-k_{\mathrm{Pr}}\cdot P_{VEN}^{*}+\sum (\frac{F_{i}}{V_{i}}P_{i,v}^{*}\;-\frac{F_{M}}{V_{M}}P_{M,v}^{*}-\frac{F_{GI}}{V_{GI}}P_{GI,v}^{*})+\frac{F_{S}+F_{GI}}{V_{L}}P_{L,v}^{*}-\lambda_{phy}\cdot P_{\;VEN}^{*}$$ (17)

$P_{\;VEN}$ and $P_{VEN}^{*}$ represent the unlabeled and labeled peptide concentrations, respectively, in venous blood. The summation terms $\sum (\frac{F_{i}}{V_{i}}P_{\;i,v}-\frac{F_{M}}{V_{M}}P_{\;S,v}-\frac{F_{GI}}{V_{GI}}P_{\;GI,v})$ and $\sum (\frac{F_{i}}{V_{i}}P_{i,v}^{*}\;-\frac{F_{M}}{V_{M}}P_{S,v}^{*}-\frac{F_{GI}}{V_{GI}}P_{GI,v}^{*})$ describes peptide flow returning from each organ’s vascular space into the venous blood, thus representing deoxygenated blood returning from tissues. This terms explicitly excludes GI and spleen because, physiologically, blood outflow from these organs is not returned directly to systemic veins but instead enters the liver. The GI and spleen flows, therefore, appear separately as $\frac{F_{S}+F_{GI}}{V_{L}}P_{\;L,v}$ and $\frac{F_{S}+F_{GI}}{V_{L}}P_{L,v}^{*}$ as combined positive inflow from the liver compartment. The term $k_{\mathrm{Pr}}$ describes clearance from the venous compartment, associated with binding to serum proteins. As the fraction of bound peptide to proteins is small compared to the total amount and to reduce complexity, only the veins were connected to serum proteins compartment.


$$\frac{d}{dt}PRP=k_{PR}\cdot P_{\;VEN}+\lambda_{phy}\cdot PRP^{*}$$ (18)

$$\frac{d}{dt}PRP^{*}=k_{PR}\cdot P_{VEN}^{*}-\lambda_{phy}\cdot PRP^{*}$$ (19)

The vascular space of each organ is the primary compartment through which peptides circulate and enter the organ’s interstitial space. The movement of free peptide between the vascular and interstitial compartments occurs via transcapillary extravasation, modeled using a permeability-surface area product $PS_{i}$. The dynamics of free peptide in the vascular compartments of general tissues (excluding kidneys and lungs) are described by:


$$\frac{d}{dt}P_{\;i,v}=PS_{i}\left(\frac{P_{\;i,int}}{V_{i,int}}-\frac{P_{\;i,v}}{V_{i,v}}\right)+F_{i}\left(\frac{P_{\;ART}}{V_{ART}}-\frac{P_{\;i,v}}{V_{i,v}}\right)+\lambda_{phy}\cdot P_{\;i,v}^{*}$$ (20)

$$\frac{d}{dt}{P_{\;i,v}}^{*}=\;PS_{i}\left(\frac{P_{i,int}^{*}\;}{V_{i,int}}-\frac{P_{i,v}^{*}\;}{V_{i,v}}\right)+F_{i}\left(\frac{P_{ART}^{*}}{V_{ART}}-\frac{P_{i,v}^{*}}{V_{i,v}}\right)-\lambda_{phy}\cdot P_{\;i,v}^{*}$$ (21)

$P_{\;i,v}$ and ${P_{\;i,v}}^{*}$ represent unlabeled and labeled peptide concentrations in the vascular compartment of organ i. Transcapillary extravasation is driven by $PS_{i}$ describing diffusion of peptide between the vascular and interstitial spaces.

The lungs have a unique role as the site of blood oxygenation. Thus, the vascular equation for the lungs includes venous inflow rather than arterial inflow.


$$\frac{d}{dt}P_{\;LU,v}=PS_{LU}\left(\frac{P_{\;LU,int}}{V_{LU,int}}-\frac{P_{\;LU,v}}{V_{LU,v}}\right)+F\left(\frac{P_{\;VEN}}{V_{VEN}}-\frac{P_{\;LU,v}}{V_{LU,v}}\right)+\lambda_{phy}\cdot P_{\;LU,v}^{*}$$ (22)

$$\frac{d}{dt}{P_{\;LU,v}}^{*}=\;PS_{LU}\left(\frac{{P_{\;LU,int}}^{*}}{V_{LU,int}}-\frac{{P_{\;LU,v}}^{*}}{V_{LU,v}}\right)+F\left(\frac{P_{\;VEN}}{V_{VEN}}-\frac{{P_{\;LU,v}}^{*}}{V_{LU,v}}\right)-\lambda_{phy}\cdot P_{\;LU,v}^{*}$$ (23)

The kidneys serve specialized roles in filtration and excretion; thus, their vascular compartment equation differs significantly.


$$\frac{d}{dt}P_{K,v}=-\frac{P_{K,v}}{V_{K,v}}\cdot (F_{fil}+F_{K})+\frac{F_{K}}{V_{ART}}\cdot P_{\;ART}+\frac{P_{\;intra,K}}{V_{intra,K}}\cdot (F_{fil}-F_{ex})+\lambda_{phy}\cdot P_{K,v}^{*}$$ (24)

$$\frac{d}{dt}P_{K,v}^{*}=-\frac{P_{K,v}^{*}}{V_{K,v}}\cdot (F_{fil}+F_{K})+\frac{F_{K}}{V_{ART}}\cdot P_{ART}^{*}+\frac{P_{intra,K}^{*}\;}{V_{intra,K}}\cdot (F_{fil}-F_{ex})-\lambda_{phy}\cdot P_{K,v}^{*}$$ (25)

$F_{fil}$ describes the filtration rate of peptide from the vascular compartment into the interstitial and intracellular spaces. $F_{ex}$ is the excretion rate of peptide out of the kidney cells, reflecting renal clearance mechanisms, which enhanced by amino acid administration.

Free peptides in the interstitial compartments represent peptides that have extravasated from the vascular space and may undergo receptor binding, internalization, or remain free. This compartment is modeled differently for PSMA-negative organs (without receptor binding) and PSMA-positive organs (with receptor binding). In PSMA-negative tissues (organs without significant peptide-specific receptor binding), the dynamics are simpler. Peptide exchange between the vascular and interstitial compartments is described solely by transcapillary extravasation. For the brain, the permeability-surface area product $PS_{i}=0$ due to the blood-brain barrier, meaning no significant interstitial transport occurs.


$$\frac{d}{dt}P_{\;i,int}=PS_{i}\left(\frac{P_{\;i,v}}{V_{i,v}}-\frac{P_{\;i,int}}{V_{i,int}}\right)+\lambda_{phy}\cdot P_{\;i,int}^{*}$$ (26)

$$\frac{d}{dt}P_{\;i,int}^{*}=PS_{i}\left(\frac{P_{\;i,v}^{*}}{V_{i,v}}-\frac{P_{\;i,int}^{*}}{V_{i,int}}\right)-\lambda_{phy}\cdot P_{\;i,int}^{*}$$ (27)

$P_{\;i,int}$ and $P_{\;i,int}^{*}$ represent unlabeled and labeled peptide concentrations in the interstitial space.

In PSMA-positive tissues (organs expressing PSMA receptors), the peptide can bind to available PSMA receptors. Thus, the equation also incorporates receptor kinetics


$$\frac{d}{dt}P_{\;i,int}=-k_{on}\cdot P_{\;i,int}\cdot \frac{R_{i}}{V_{i,int}}+k_{off}\cdot RP_{i}+PS_{i}\left(\frac{P_{\;i,v}}{V_{i,v}}-\frac{P_{\;i,int}}{V_{i,int}}\right)+\lambda_{phy}\cdot P_{\;i,int}^{*}$$ (28)

$$\frac{d}{dt}P_{\;i,int}^{*}=-k_{on}\cdot P_{\;i,int}^{*}\cdot \frac{R_{i}}{V_{i,int}}+k_{off}\cdot R{P_{\;i}}^{*}+PS_{i}\left(\frac{P_{\;i,v}^{*}}{V_{i,v}}-\frac{P_{\;i,int}^{*}}{V_{i,int}}\right)-\lambda_{phy}\cdot P_{\;i,int}^{*}$$ (29)

$k_{on}$ and $k_{off}$ are kinetic rate constants for receptor binding and dissociation. $R_{i}$ represents the concentration of free PSMA receptors available for binding. $RP_{i}$ and $R{P_{\;i}}^{*}$ represent unlabeled and labeled peptide bound to receptors on cell surfaces.

The interstitial compartment in kidneys includes receptor-binding kinetics as well as filtration-specific terms.


$$\frac{d}{dt}P_{\;K,int}=-k_{on}\cdot P_{\;K,int}\cdot \frac{R_{K}}{V_{K,int}}+k_{off}\cdot RP_{K}+F_{fil}\left(\frac{P_{\;K,v}}{V_{K,v}}-\frac{P_{\;K,int}}{V_{K,int}}\right)+\lambda_{phy}\cdot P_{\;K,int}^{*}$$ (30)

$$\frac{d}{dt}P_{\;K,int}^{*}=-k_{on}\cdot P_{\;K,int}^{*}\cdot \frac{R_{K}}{V_{K,int}}+k_{off}\cdot RP_{K}^{*}+F_{fil}\left(\frac{P_{\;K,v}^{*}}{V_{K,v}}-\frac{P_{\;K,int}^{*}}{V_{K,int}}\right)-\lambda_{phy}\cdot P_{\;K,int}^{*}$$ (31)

The kidney compartment additionally accounts for unspecific intracellular uptake and clearance. This unspecific process is independent of receptor-mediated binding.


$$\frac{d}{dt}P_{\;intra,K}=\frac{P_{\;int,K}}{V_{int,\;K}}\cdot (F_{fil}-F_{ex})-\frac{P_{\;intra,K}}{V_{intra,K}}\cdot (F_{fil}-F_{ex})+\lambda_{phy}\cdot P_{intra,K}^{*}$$ (32)

$$\frac{d}{dt}P_{intra,K}^{*}=\frac{P_{int,K}^{*}\;}{V_{int,\;K}}\cdot (F_{fil}-F_{ex})-\frac{P_{intra,K}^{*}}{V_{intra,K}}\cdot (F_{fil}-F_{ex})-\lambda_{phy}\cdot P_{intra,K}^{*}$$ (33)

$P_{\;intra,K}$ and $P_{intra,K}^{*}$ represent the unlabeled and labeled peptide concentrations in kidney cells due to unspecific uptake.

For PSMA-positive tissues the PBPK model explicitly includes receptor-mediated binding and internalization of peptides. These dynamics are described by two sets of equations: one for receptor binding at the cell surface, and the other for internalization into cells.

The receptor-bound peptide dynamics are modeled by:


$$\frac{d}{dt}RP_{\;i}=k_{on}\cdot P_{\;i,int}\cdot \frac{R_{i}}{V_{i,int}}-(k_{off}+\lambda_{int,i})\cdot RP_{\;i}+\lambda_{phy}\cdot RP_{\;i}^{*}$$ (34)

$$\frac{d}{dt}R{P_{\;i}}^{*}=k_{on}\cdot {P_{\;i,int}}^{*}\cdot \frac{R_{i}}{V_{i,int}}-(k_{off}+\lambda_{int,i})\cdot R{P_{\;i}}^{*}-\lambda_{phy}\cdot RP_{\;i}^{*}$$ (35)

With the receptor site constraint


$$R_{0,\;i}=R_{i}+RP_{\;i}+RP_{\;i}^{*}$$ (36)

$RP_{\;i}$ and $RP_{\;i}^{*}$ represent the unlabeled and labeled peptides bound to PSMA receptors at the cell surface. $R_{i}$ is the concentration of free receptors available for binding, and $R_{0,\;i}$ is the total receptor density (bound + free receptors), a fixed physiological parameter. $\lambda_{int,i}$ denotes the internalization rate constant, reflecting peptide-bound receptor internalization into the cell.

Once the peptide binds to PSMA receptors on the cell surface, it undergoes internalization into the cellular compartment, represented by:


$$\frac{d}{dt}{P_{intern}}_{\;i}=\lambda_{int,i}\cdot RP_{\;i}-\lambda_{release,\;i}\cdot {P_{intern}}_{\;i}+\lambda_{phy}\cdot {{P^{*}}_{intern}}_{\;i}$$ (37)

$$\frac{d}{dt}{{P^{*}}_{intern}}_{\;i}=\lambda_{int,i}\cdot R{P^{*}}_{\;i}-\lambda_{release,\;i}\cdot {{P^{*}}_{intern}}_{\;i}-\lambda_{phy}\cdot {{P^{*}}_{intern}}_{\;i}$$ (38)

$P_{\;i,intern}$ and ${P_{\;i,intern}}^{*}$ represent unlabeled and labeled internalized peptide within cells. $\lambda_{release,\;i}$ represents the degradation (clearance) rate of peptide and radionuclide from inside the cell.

Due to the emission range of ^177^Lu [115], we included a 2mm layer of surrounding host tissue (muscle) in our calculation of the tumor absorbed dose to account for background correction. The influence of peptides in the muscle on the tumor was calculated using equation 39 as follows:


$$C_{musle,\;emission\;range}^{*}=\;c_{BG}.\left(C_{mus,v}^{*}+C_{mus,int}^{*}+PRP^{*}.\left(\frac{V_{mus,v}}{V_{p}+V_{tumor},v}\right)\right)$$ (39)

The background correction of labeled peptides in the muscle is denoted by $c_{BG}$ (Table 1). The concentration of labeled peptides in vascular and extracellular space of muscles represented by $C_{mus,v}^{*}\;$and$\;C_{mus,int}^{*}$, respectively. A proportion of peptides that bound to proteins of blood flow, designated by$\;PRP^{*}$, also considered in absorbed dose calculation which is influenced by the volume of capillaries in tumor ($V_{tumor},v$) and muscle ($V_{mus,v}$), and the volume of total body serum ($V_{p}$). (See S1 Text for details)

### 2.2. Amino acid administration

To block un-specific uptake and thus increase the ratio of tumor-to-kidney absorbed dose, co-administration of amino acids have been undertaken [43]. Pre-administration of amino acids before RPT can significantly mitigate toxicity risks. Certain amino acids, notably lysine and arginine, compete with radiopharmaceuticals for reabsorption in the renal tubules [42,43,97]. This competitive inhibition diminishes radiopharmaceutical uptake by the kidneys, thus reducing radiation exposure to all organs while enhancing the clearance of both labeled and unlabeled peptides from the body [52]. Consequently, AAI emerges as a valuable tool for molecular imaging and RPT, especially in cases where the kidneys are OAR for potential radiation damage. Conversely, the accelerated clearance rate resulting from amino acid infusion can decrease the concentration of radiopharmaceuticals circulating in the body, thereby impacting the absorbed dose of tumors.

The kidneys were explicitly modeled (Fig A in S1 Text) as they are one of critical organs due to PSMA receptor expression and unspecific uptake (which is not entirely blocked by the administration of amino acids). Typically, ligand clearance rates from kidney, excretion rate ($f_{ex}$), (Fig A and Table A in S1 Text) are around 80%, but amino acid infusion elevates this rate to 96% [97]. The objective is to optimize the dosage of injected radiopharmaceuticals for each patient, aiming to enhance treatment efficacy by increasing the absorbed dose to the tumor while ensuring that critical toxicity thresholds in healthy organs are not surpassed. Initially, the kidney is designated as the OAR, and the effect of AAI on the tumor is evaluated. Subsequently, the salivary gland is considered as the OAR. The tumor volume is assumed to be 2ml for all patients, with a geometry previously described. Ultimately, the concentrations of radiopharmaceuticals in tumor vasculature, both with and without AAI, are calculated using the PBPK model. These calculated concentrations used as inputs for the CDR equations, enabling the determination of tumor absorbed doses.

### 2.3. Absorbed dose

The absorbed dose equivalent was calculated using equation 40, as per the MIRD formulation.


$$D_{i}(T)=\int_{0}^{T}\overset{\cdot }{D_{i}}(t)dt=A_{0}.\sum {\tilde{a}}_{i}(T).S_{i\leftarrow i}$$ (40)

With the absorbed dose $D_{i}(T)$ to organ i (where T = 30000 min), absorbed dose rate ${\dot{D}}_{i}(t)$, the injected activity $A_{0}$, the time-integrated activity coefficient ${\tilde{a}}_{i}(T)$, the dose factor organ i to organ *I* ($S_{i\leftarrow i}$*).* The S-values for each tumor lesion and OARs were determined based on the data of OLINDA/EXM for ^177^Lu for spheres [116]. For the tumors, kidneys and salivary glands, the self-dose and a segment of the labeled peptides that bound to serum proteins were taken into account. Tumor absorbed doses were also consider background correction which is incorporating of a portion of labeled peptides in muscle.


$${\overset{\cdot }{D}}_{i}(t)=A_{i}(t)\cdot S_{i\leftarrow i}=A_{0}\cdot a_{i}(t)\cdot S_{i\leftarrow i}$$ (41)

### 2.4. Statistical analysis and sensitivity analysis

To address the concern regarding the necessity of the model complexity (coupling PBPK and CDR equations) and the potential for use of just a macroscopic approach (only PBPK model) with fewer parameters, we implemented a statistical analysis using the Wilcoxon Signed-Rank Test [117]. The Wilcoxon Signed-Rank Test was chosen due to its suitability for paired data and its non-parametric nature, which does not assume normality of the data. This test was employed to compare the absorbed dose calculated by the PBPK model and CDR equations, focusing on data from 12 points (absorbed dose due to presence of radiopharmaceuticals in tumor interstitial space, tumor cell receptors, and internalized within tumor cells) for four patients. Moreover, to evaluate the effect of AAI on tumor absorbed dose, we analyzed the statistical significance of tumor absorbed dose with and without AAI, irrespective of whether the kidneys or salivary glands were designated as the OAR, using the Wilcoxon Signed-Rank Test.

To conduct a sensitivity analysis, we used normalized sensitivity indices (SI) to quantify the relative impact of parameter variations on the absorbed dose. The SI for a parameter Pi was calculated using the formula: SI = (ΔDi/Di)/ (ΔPi/Pi), where ΔDi is the change in the output (absorbed dose), Di is the baseline output value, ΔPi is the change in the parameter value, and Pi is the baseline parameter value. We systematically varied each parameter across four scaling ranges—100Pi → 10Pi, 10Pi →  Pi, Pi → 0.1Pi, and 0.1Pi → 0.01Pi —while keeping all other parameters constant. In this analysis, the tumor serum flow density (f_tumor), permeability surface area product per unit mass (k_tumor), association rate (k_on), dissociation rate (k_off), internalization rate (lambda_int), physical decay (lambda_phy), PSMA binding site density (RD), and release rate from tumor cells (lambda_release) was varied across four orders of magnitude: 100, 10, 0.1, and 0.01, to assess its effect on the tumor absorbed dose. The corresponding normalized sensitivity indices expressed as mean ± standard deviation. The analysis provided insights into the sensitivity of the system by identifying parameter ranges that elicited significant changes in output.

### 2.5. Solution strategy and assumptions

**Fig 2:**
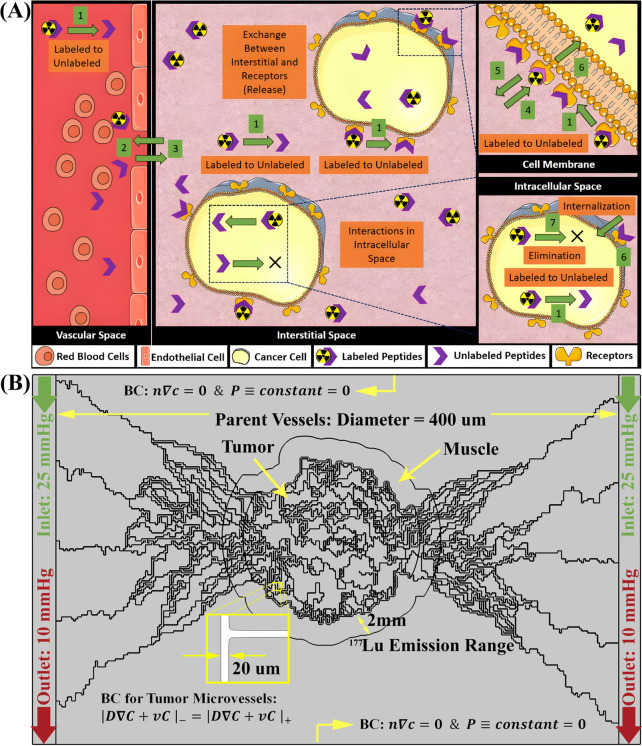
Multi‑Compartment PBPK model and spatial simulation domain for radiopharmaceutical transport in tumor tissue. (A) Solution domain and microvascular network distribution and Schematic of the Multi-Compartment Model for tumor in radiopharmaceutical therapy. This diagram represents a multi-compartmental physiologically based pharmacokinetic model detailing the distribution and interaction radiopharmaceuticals within four distinct physiological spaces: vascular, interstitial, cell membrane, and intracellular compartments. The transient mass transfer simulation incorporates convective contributions and applies the multi-compartment model to track solute dynamics across the biophysical domains. Key Steps: 1: Physical decay of radionuclide; 2 & 3 Exchange of radiopharmaceuticals between the vascular and interstitial compartments; 4 & 5: Binding and subsequent unbinding of the free drug to receptors on cell membrane; 6: Internalization of peptides that bound to receptors into intracellular space; 7: clearance of radiopharmaceuticals. Image created using Servier Medical Art (https://smart.servier.com/), licensed under CC BY 4.0. **(B)** Illustration of Solution Domain with Microvascular Network Distribution and Boundary Conditions. The simulation focuses on a tumor with an approximate diameter of 16 mm, embedded in a domain that also includes adjacent normal tissue. The microvascular network comprises vessels with diameters around 20 μm, branching from larger parent vessels of about 400 μm in diameter [118]. Vascular architecture is generated based on the sprouting angiogenesis model originally formulated by Anderson and Chaplain [57,58], with vessel density increasing in response to local concentrations of tumor angiogenic factors. This leads to a complex capillary network with enhanced branching and loop formation in the tumor region. The domain extends 2 mm beyond the tumor margin to capture the full therapeutic range of ^177^Lu, with emphasis placed on modeling both intratumoral and peritumoral transport phenomena.

Investigating a tumor measuring approximately 16 mm in diameter, this study considered a microvasculature that possess a diameter of 20 micrometers [118], while the parent vessels measure 400 micrometers in diameter. The angiogenesis model employed is according to the sprouting angiogenesis model initially proposed by Anderson and Chaplain [57,58]. This mathematical framework forecasts the formation of microvasculature by tracing the movement of endothelial cells located at the tip of the microvascular sprout, branching and anastomosis, culminating in the establishment of a microvascular network. The concentration of tumor angiogenic factors correlates with heightened microvascular density in the tumor area, characterized by an augmented number of branches and loops. While normal host tissue is also considered, the primary emphasis of this investigation centers on the tumor tissue and the surrounding region, extending 2mm from the tumor boundary, which corresponds to the emission range of ^177^Lu (refer to Fig 2).

The problem-solving approach comprises three primary components: a steady-state solution and a time-dependent solution. The steady-state solution addresses intravascular and interstitial fluid flow, characterized by the application of constant pressure boundary conditions at the inlet and outlet of the parent vessels (25 and 10 mmHg, respectively). Subsequently, the PBPK model is employed to ascertain the temporal distribution of peptides in organs and the vascular space of the tumor, serving as input for the CDR equations. Following the steady-state solution, the time-dependent analysis of the mass transfer equations is conducted to incorporate the influence of convection in solute transport. This involves the transportation of solutes within the biophysical compartments based on a multi-compartment model (Fig 2). To solve the coupled nonlinear set of governing equations and boundary conditions, the finite element method is employed. The simulation utilizes the commercial finite element software COMSOL Multiphysics 6.1 (COMSOL, Inc., Burlington, MA, USA). For numerical computation, a segregated approach is adopted with a time-step of 1 minute and a relative tolerance of 0.0001. The drug delivery analysis spans a time period of 30000 minutes. To ensure the stability and accuracy of the numerical method, a mesh refinement study was conducted. The final selected mesh consisted of 6,371,314 elements, determined to balance computational efficiency and solution accuracy. Convergence of the solution was verified by comparing results across different mesh densities, confirming that further refinement produced negligible changes in the solution.

The findings of this investigation are based on several crucial assumptions that have been considered. These fundamental assumptions are outlined as follows.

- In this modeling analysis, it is assumed that the parameters governing drug transport and the geometrical characteristics of the study system remain constant because of the short time period of investigation.
- To compute absorbed dose for tumor peptides in vascular space, interstitial space, receptors on the cell membrane, intracellular space, and a proportion of peptides in host tissue considered as mentioned in equations.
- An important limitation of this study is the two-dimensional nature of the tumor model, which was chosen to manage computational cost and equation complexity. Although a three-dimensional approach would offer a more comprehensive representation of tumor behavior and radiopharmaceutical transport, the 2D model effectively captures critical biological phenomena [57,58], such as endothelial cell migration, capillary formation, and the distribution of therapeutic agents in cross-sections [55,56].
- Tumor growth and angiogenesis are inherently dynamic processes, characterized by continuous changes over time. However, due to the complexity of modeling radiopharmaceutical transport, we have adopted a simplifying assumption: the tumor size is treated as constant, and the microvasculature is considered fixed over the 21-day study period. This approach allows us to isolate and focus specifically on the mechanisms of radiopharmaceutical transport without the added variability introduced by concurrent changes in tumor morphology and vascular dynamics.
- The model assumes a stochastic framework to capture the randomness of angiogenesis while effectively representing tumor heterogeneity. It does not account for variations in vessel diameters or regional differences such as necrotic cores, hypoxic zones, or proliferative areas, presuming a relatively homogeneous tumor tissue composition. This approach, while insightful for general dynamics, may require refinement for application to specific tumor types.

## 3. Results

In this section, we thoroughly examine the research outcomes within the framework of relevant mathematical equations and underlying assumptions. Subsequently, we showcase the synthesized microvasculature and validate the model using optical frequency domain imaging. Following this, we estimate fluid flow within both vascular and interstitial spaces, aiming to discern the extent of the convective mechanism’s influence on solute transit within the biophysical environment. Next, we evaluate the effects of amino acids using a PBPK model, and employ the resulting data to simulate responses using CDR equations.

### 3.1. Fluid flow

**Fig 3:**
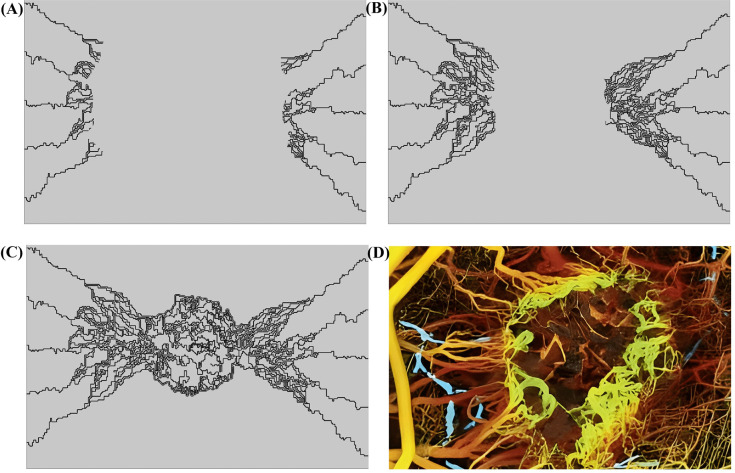
The modeling of angiogenesis process over a span of 30 days is depicted and compared qualitatively with actual microvasculature surrounding and within tumors. The models demonstrate that newly formed blood vessels near and within the tumor exhibit significant branching and looping, consistent with in vivo observations. Comparing angiogenesis models that correspond with in vivo studies highlights that newly formed blood vessels near and within the tumor exhibit significant branching and looping [67]. The timeline includes snapshots at specific intervals: **(A)** Day 5, **(B)** Day 25, and **(C)** Day 30, showing the progression of vascular development in the model. Additionally, (D) represents tumor vasculature captured through optical frequency domain imaging [119], serving as a reference for comparison.

To explore the dynamics of fluid movement in blood vessels and the spaces between cells, our study focuses on a model microvascular network initiated by two parent vessels placed at opposite ends of the designated area (see Fig 2B). This setup is exposed to a concentration gradient of tumor angiogenesis factors (TAFs) across the region, which triggers the formation of new blood vessels from the original ones through a process called sprouting angiogenesis. These emerging vessels then go through various transformations; branching, anastomosis, and grow towards the tumor. As these new microvessels develop, they multiply and organize into complex networks of microvascular loops within the tumor environment, as illustrated in Fig 3. This figure provides a visual comparison that highlights the similarities between our simulation outcomes and the actual biological patterns observed in tumor angiogenesis. In line with real-life biological findings, our models reveal an increase in microvascular branching, particularly around and inside the tumor boundary, corresponding to the areas with higher TAF concentrations. This branching effect is attributed to the action of TAFs, which encourage the sprouting of new endothelial cell tips (tECs) and the formation of more vessel branches.

The dynamics of interstitial fluid, characterized by IFP and IFV, are crucial in the progression of cancer and the development of blood vessels in solid tumors and adjacent tissues. A significant feature of solid tumors is the elevation of IFP, which hinders transvascular exchange through convective transport mechanisms. This heightened IFP poses challenges in the therapeutic management of tumors by potentially causing fluid to exit the tumor, leading to the potential displacement of radiopharmaceuticals. The increase in IFP can be attributed to various pathophysiological conditions such as irregularities in the tumor’s blood vessels, dysfunctional lymphatic drainage, alterations in the tumor microenvironment, and the dense accumulation of tumor cells within a confined space. This pressure gradient results in outward radial convection, which contrasts with the inward diffusion of substances. Since PBPK models do not inherently incorporate velocity fields, they are limited to considering diffusion-only transvascular exchange between the vascular and interstitial spaces. However, in order to assess the impact of IFP and IFV, CDR equations are coupled with PBPK models following the simulation of pressure distribution.

**Fig 4:**
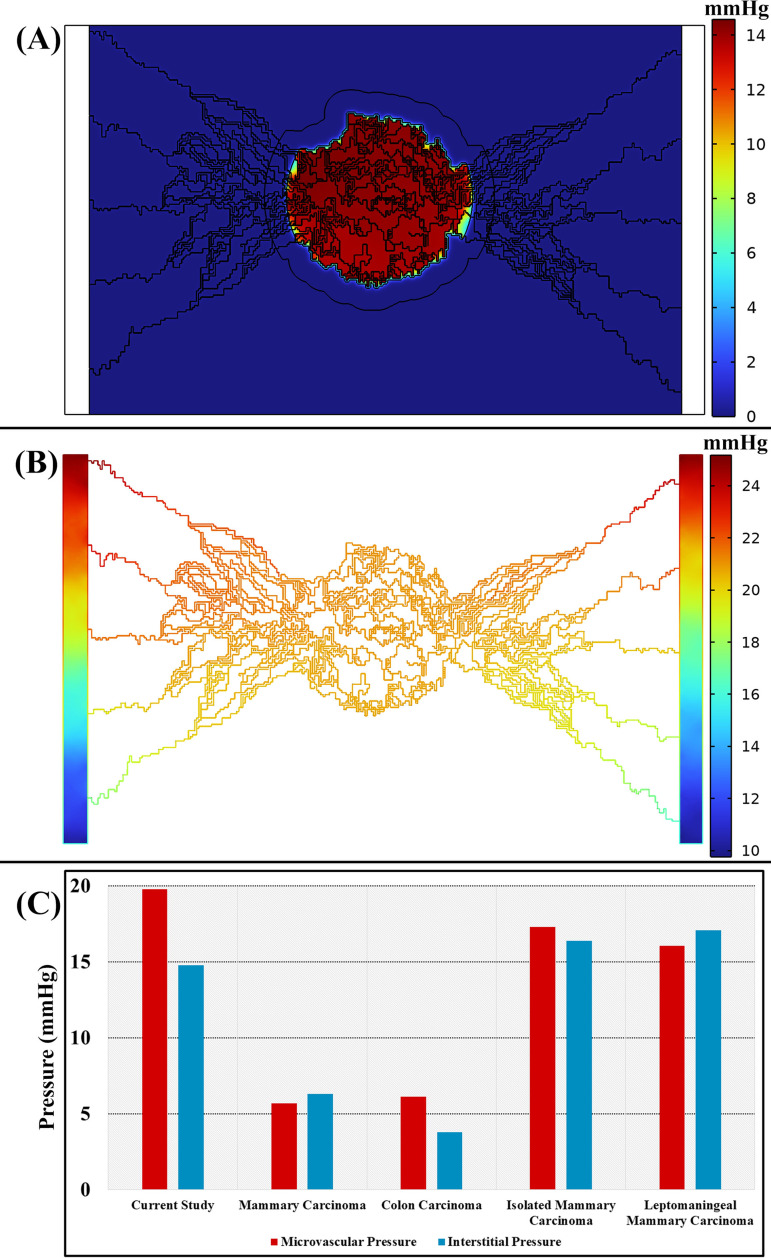
Interstitial fluid pressure (IFP) and intravascular fluid pressure (IVP) validation; (A) Simulated distribution of IFP and (B) IVP within the tumor microenvironment. These simulations illustrate the elevated internal pressures characteristic of solid tumors. Notably, when an inlet pressure of 25 mmHg is applied, the corresponding microvascular pressure reaches 19.8 mmHg, while the interstitial fluid pressure rises to 14.8 mmHg, indicating a significant internal buildup of pressure within the tumor tissue. **(C)** An analysis correlating intravascular and interstitial pressure across various tumors in realistic models suggests that both pressures are elevated with a minimal disparity between them [122]. This trend is attributed to structural abnormalities in the tumor vasculature, dysfunctional lymphatic drainage, and the dense packing of tumor cells. Elevated IFP impedes convective transvascular transport and can hinder the delivery and retention of therapeutic agents by promoting outward fluid convection, potentially facilitating drug efflux from the tumor. While this study does not account for lymphatic drainage, monitoring IFP and IFV remains vital for understanding drug delivery dynamics and improving therapeutic strategies in solid tumors.

Monitoring IFP and IFV can offer crucial insights that could inform the development of effective RPT. For instance, in Fig 4A and 4B, the distribution of IFP is depicted under an inlet pressure of 25 mmHg. In this scenario, the IFP within the tumor microvasculature registers at 19.8 mmHg, resulting in a significantly elevated IFP of 14.8 mmHg. Comparing the microvascular pressure and IFP across various tumor tissues reveals a marked increase in internal tumor pressure, closely resembling the pressure observed within blood vessels [120,121]. This heightened pressure can expel radiopharmaceuticals from the tumor tissue, facilitating their diffusion back into the bloodstream. The observed elevation in IFP is often attributed to the enhanced permeability of the tumor’s blood vessels, while an inefficient lymphatic drainage system within the tumor hampers the removal of accumulated fluids and substances. It is important to note that our study does not take into account the tumor’s lymphatic drainage system. The difference between tissue pressure and vascular pressure plays a pivotal role in influencing the transfer of radiopharmaceuticals through the walls of blood vessels, as this transfer hinges on the convective transport component. In contrast, IFV reaches its minimum level within the tumor mass, with its peak values occurring exclusively at the interface where the tumor intersects with healthy tissue. It is at this boundary where the pressure gradient promotes motion and augments flow (Fig 4A).

### 3.2. Spatiotemporal distribution of radiopharmaceuticals in tumor

**Fig 5:**
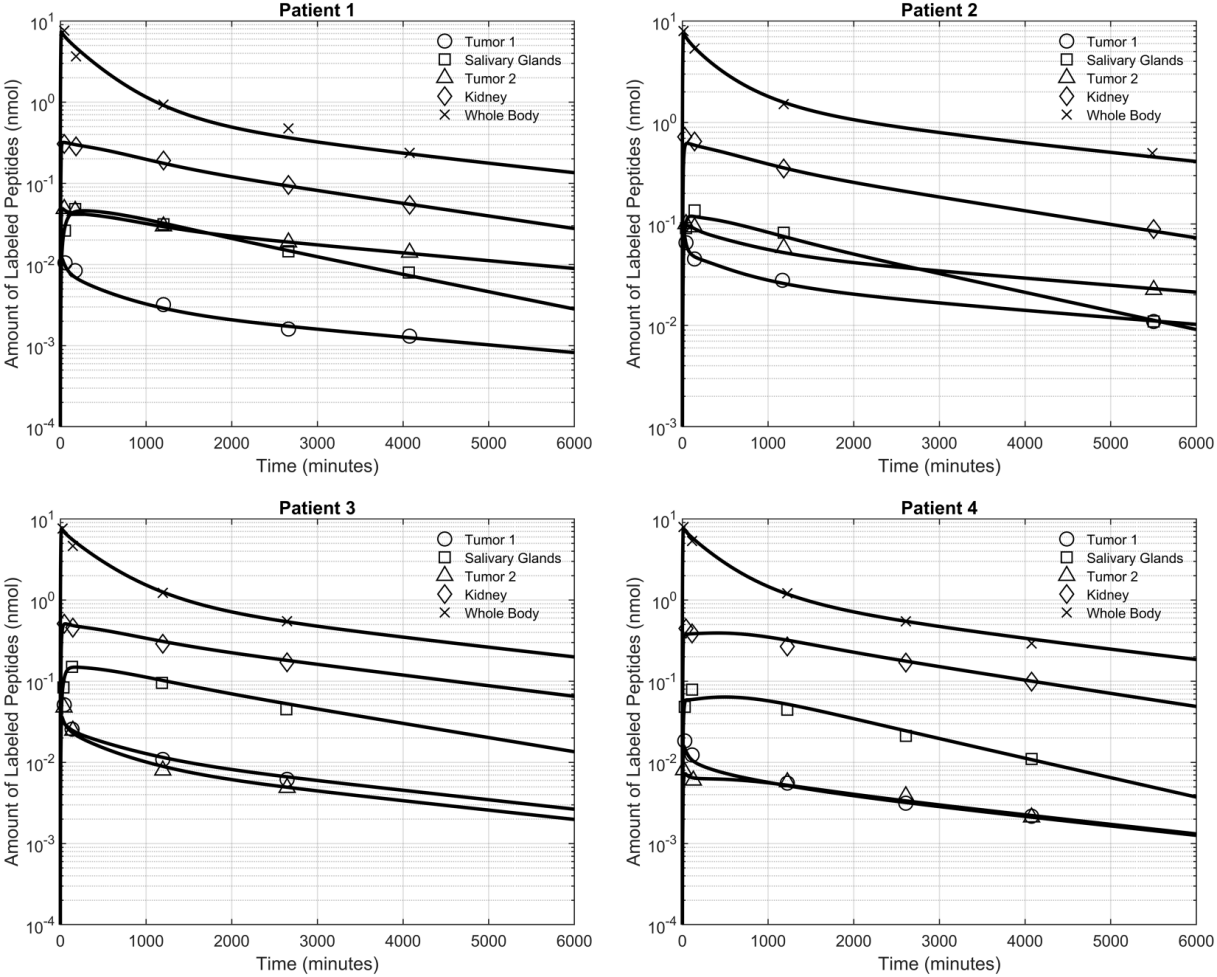
Validation of PBPK model and parameter estimation. By employing post-treatment data obtained from γ-camera images for four patients [42], the designed PBPK model is fitted to the acquired data, facilitating the customization of RPT for personalized treatment strategies. Details of the fitting procedures—including optimization techniques and convergence criteria—as well as the statistical outcomes of the parameter estimation are provided in Figs A-D and Tables C-F in S2 Text.

Fitting of the developed PBPK model to data collected from γ-camera imaging leads to the determination of individual parameters for four patients, thus generating a personalized virtual representation of each patient for precise RPT implementation. The disparity between the fitted time activity curve (TAC) and the γ-camera imaging data points for tumors and organs-at-risk was assessed through metrics including mean squared error (MSE), sum of squared errors (SSE), Akaike information criterion (AIC), and Bayesian information criterion (BIC), resulting in average values of $2.4\times 10^{-11}$, $1.53\times 10^{-10}$, −439, −430, and a Log-Likelihood of 230  among all patients, respectively. Furthermore, the performance of the developed PBPK model was visually compared with data from Kletting et al. [42] for all four patients (Fig 5). However, PBPK models, by their very nature, offer only temporal concentration profiles of radiopharmaceuticals in organs, thus making it challenging to incorporate the effects of IFP and IFV on RPT. Consequently, alternative methodologies are required. As discussed earlier, the distribution of IFP is delineated by the microvasculature generated, allowing for the simulation of spatiotemporal distributions through the application of CDR equations.

The injection of ^177^Lu-PSMA presents an opportunity for theranostics, enabling simultaneous treatment and imaging. PSMA ligands target receptors on the surface of cancer cells, with beta emissions from ^177^Lu targeting these cancer cells. Additionally, the gamma emissions from ^177^Lu facilitate γ-camera scans, aiding in the localization of tumor lesions within the body. In nuclear medicine, various factors can degrade image quality. Some of these are inherent to the imaging device, such as spatial resolution, energy resolution, non-uniformity, or distortions. Other factors depend on the patient and organ localization. For instance, larger patients can increase the influence of scattered photons, while organs deep within the body may be obscured by surrounding tissues, leading to increased background registrations. Patient and organ movements can also degrade image quality. To mitigate inaccuracies in molecular imaging, multiple corrections are commonly implemented. These corrections include attenuation [123–127], scatter [126–128], partial volume [129–131], among others. By implementing these corrections, the resulting image can better approximate the distribution of radiopharmaceuticals, moving closer to representing reality. It is important to acknowledge that despite these corrections, the image remains an approximation and does not fully capture the true distribution of radiopharmaceuticals within the body. However, in this study, we did not rely on imaging-based corrections. Instead, we employed mathematical modeling and simulations to capture the true distribution of radiopharmaceuticals. Transport phenomenon equations in biological tissue represent a potent tool for elucidating the intricate distribution within tissues.

**Fig 6:**
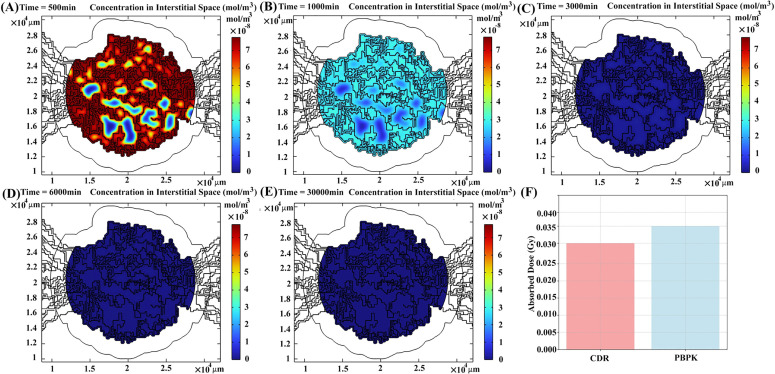
The spatiotemporal distribution of radiopharmaceuticals in tumor interstitial space at (A) 500 min, (B) 1000 min, (C) 3000 min, (D) 6000 min, and (E) 30000 min post-treatment for patient 1. **(F)** Different absorbed doses (due to contribution of peptides in interstitial space) calculated by PBPK vs. CDR models.

Fig 6 illustrates the distribution of labeled peptides within the heterogeneous interstitial space of tumors at various time intervals post-injection: 500, 1000, 3000, 6000, and 30000 minutes. The concentration of radiopharmaceuticals in vascular compartment, derived through the use of the PBPK model, serves as input for the CDR equations to simulate spatiotemporal distribution in other compartments.

As observed, the concentration of labeled radiopharmaceuticals within the interstitial space of tumors diminishes over time due to several factors. These include binding to receptors on cancer cells, returning to the vascular space due to IFP, or physical decay. Initially, the concentration is highest in the vicinity of microvessels (Fig 6A and 6B). However, as radiopharmaceuticals are cleared from the interstitial space to the vascular space, regions farther from microvessels accumulate higher concentrations compared to those nearer to microvessels (Fig 6C and 6D). Ultimately, the labeled peptide is either cleared from the tumor or undergoes decay (Fig 6E).

The tumor absorbed dose is computed based on concentrations of labeled peptides in the interstitial space derived separately from the PBPK model and CDR equations (Fig 6F). The CDR model indicates approximately a 7% ± 4.1% (mean ± SD) lower absorbed dose, with the primary contributing factor being the influence of IFP distribution within the tumor (Table B in S3 Text). Elevated concentrations in the vascular space prompt the inward transport of radiopharmaceuticals. However, the presence of high IFP within the tumor acts as a repulsive force on peptides, compelling them to retreat back to the microvasculature. Moreover, the heterogeneous distribution of microvessels also plays a role, resulting in a smaller quantity of peptides calculated using the CDR equation. Regions characterized by a higher density of microvasculature not only exhibit increased IFP but also harbor higher concentrations, thereby facilitating the possibility of retrograde flux from the interstitial space to the vascular space.

**Fig 7:**
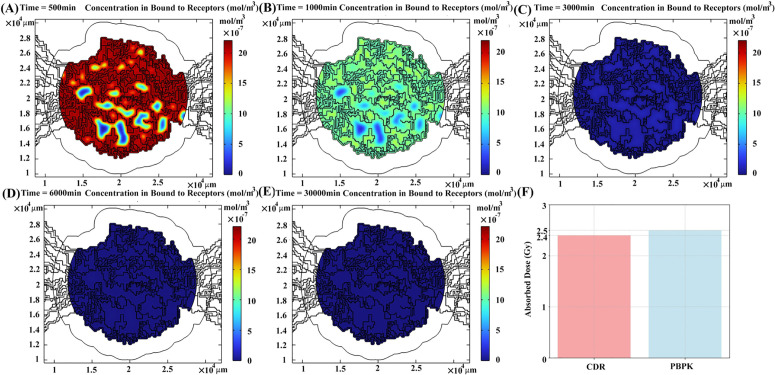
The spatiotemporal distribution of radiopharmaceuticals that bound to receptors at (A) 500 min, (B) 1000 min, (C) 3000 min, (D) 6000 min, and (E) 30000 min post-treatment for patient 1. **(F)** Different absorbed doses (due to the contribution of peptides bound to receptors) calculated by PBPK vs. CDR models.

Radiopharmaceuticals within the interstitial space exhibit a propensity to bind to receptors on cancer cells, initiating a competitive interaction between labeled and unlabeled peptides for these PSMA receptors (Fig 7). Consequently, two systems of equations are considered, one for labeled peptides and another for unlabeled peptides, which are linked through this competition and physical decay process. A closer examination of the distribution of radiopharmaceuticals bound to receptors reveals that cancer cells near microvasculature exhibit a higher association with PSMA ligands compared to other cells. This phenomenon arises from the elevated concentration of peptides in these regions of the interstitial space, facilitating greater opportunities for association. The distribution pattern of radiopharmaceuticals bound to tumor receptors mirrors their distribution within the interstitial space, with the highest concentrations observed at the onset of treatment and in close proximity to microvasculature (Fig 7A and 7B). Over time, the density of peptides in regions distant from microvasculature exhibits a higher concentration compared to other regions. This phenomenon is attributed to the elevated concentration of peptides in those interstitial space regions (Fig 7C and 7D). Ultimately, the majority of radionuclides are either cleared or undergo decay within the tumor (Fig 7E). The absorbed dose resulting from labeled peptides bound to receptors is calculated using concentrations obtained from PBPK models and CDR equations, revealing a 3.3% ± 1.4% (mean ± SD) difference (Fig 7F and Table B in S3 Text). This discrepancy arises because CDR equations indicate a lower concentration in the interstitial space compared to the PBPK model.

**Fig 8:**
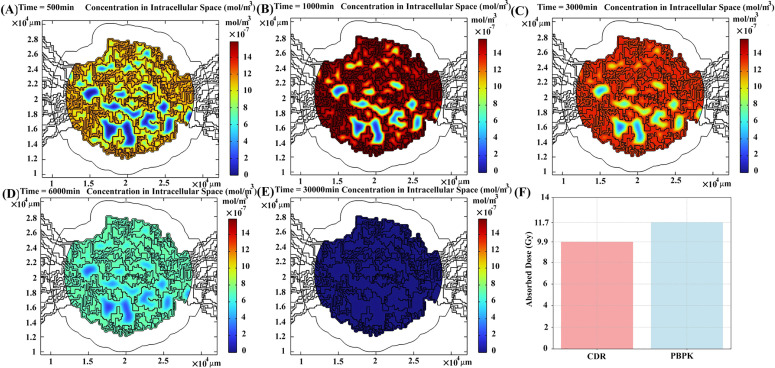
The spatiotemporal distribution of radiopharmaceuticals that internalized into tumor cells at (A) 500 min, (B) 1000 min, (C) 3000 min, (D) 6000 min, and (E) 30000 min post-treatment for patient 1. **(F)** Different absorbed doses (due to the contribution of peptides internalized into cells) calculated by PBPK vs. CDR models.

Peptides bound to receptors undergo either dissociation from receptors or internalization into cancer cells, with a significant portion of the absorbed dose in tumors attributable to internalized radiopharmaceuticals. In contrast to peptides in the vascular space and interstitial space, the internalization of radiopharmaceuticals into cancer cells is a process that requires more time. Consequently, the concentration initially increases (Fig 8A and 8B) and subsequently decreases over time due to physical decay and degradation of radiopharmaceuticals within cancer cells. Once internalized, radiopharmaceuticals do not return to the interstitial space from within cancer cells but remain within the cells until they undergo degradation. Consequently, their density consistently remains higher in the vicinity of microvasculature throughout the treatment process (Fig 8C and 8D). A comparison of the absorbed dose calculated by the PBPK model and CDR equation reveals around 11% ± 2.6% (mean ± SD) difference (Fig 8F and Table B in S3 Text).

Comparing our results (Figs 6F-8F and Table B in S3 Text) with those derived from the PBPK model presented by Kletting et al. [42] demonstrates the validity of our model in the context of personalized RPT. This comparison highlights the consistency of our findings with existing literature while showcasing the added value of incorporating tumor-specific parameters, such as IFP distribution and microvascular heterogeneity, and flux dynamics to enhance the precision and applicability of our approach.

The Wilcoxon Signed-Rank Test allowed us to compare the absorbed dose calculated by the PBPK model and CDR equations, focusing on data from 12 points (absorbed dose due to presence of radiopharmaceuticals in tumor interstitial space, tumor cell receptors, and internalized within tumor cells) for four patients, and assess whether the absorbed dose results from the two approaches were statistically similar (Table B in S3 Text). The analysis yielded a p-value of 0.0004, leading to the rejection of the null hypothesis and confirming that the two sets are significantly different. This result highlights the importance of the model’s complexity in accurately capturing the critical processes governing radiopharmaceutical distribution and uptake in the tumor microenvironment (Table B in S3 Text).

**Fig 9:**
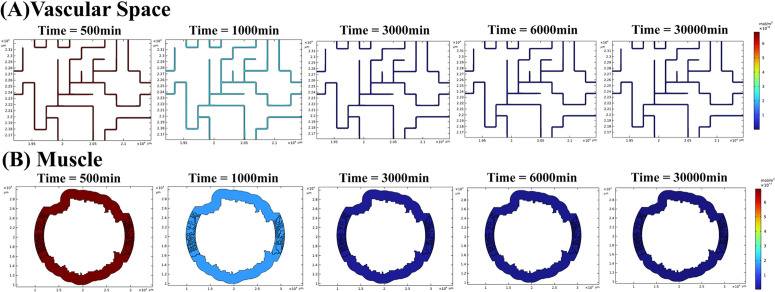
The spatiotemporal distribution of radiopharmaceuticals is shown for (A) the tumor vascular space and (B) the amount of radiopharmaceuticals in muscle that affect tumor cells, for patient 1, at different time points during treatment.

The two final compartments that significantly influence tumor absorbed dose are the vascular space and the presence of radiopharmaceuticals in the muscle surrounding the tumor, which is considered the host organ for the tumor. As previously discussed, the concentrations in these two spaces are estimated using a personalized PBPK model for each patient. It is assumed that the radiopharmaceuticals have a uniform distribution in these regions (Fig 9). The vascular compartment encompasses the concentration of labeled peptides within the tumor microvasculature as well as peptides that bind to serum proteins. On the other hand, the muscle compartment accounts for the background effect, which implies that labeled peptides present within a 2mm radius of the tumor may impact cancer cells. According to our findings and the simulated distribution of radiopharmaceuticals, cancer cells situated near the tumor microvasculature are more effectively targeted compared to cells located farther away. Additionally, cells at the tumor margin are influenced by radiopharmaceuticals present in host organs, alongside the labeled peptides within the tumors themselves. As a result, the presence of radiopharmaceuticals in host organs could potentially have a beneficial impact on cancer treatment, provided that these organs are not considered at risk and that the radiation toxicity does not surpass its critical threshold. This suggests that leveraging the distribution of radiopharmaceuticals in host organs could enhance therapeutic outcomes, particularly if the radiation dosage remains within safe limits for surrounding tissues.

### 3.3. Effect of amino acid administration

**Fig 10:**
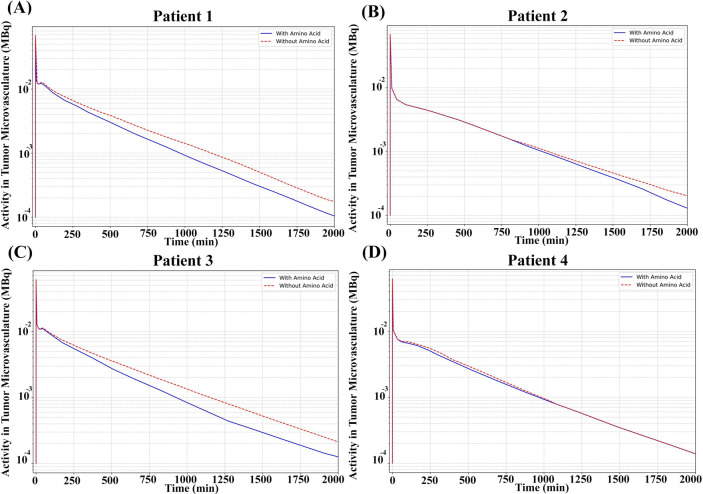
Comparison of radiopharmaceutical concentration in tumor vascular space, with and without amino acid administration, across 4 mCRPC patients. The figures show a decrease in the concentration of radiopharmaceuticals in the tumor vasculature following the administration of amino acids.

Through the use of PBPK modeling, the influence of AAI on clearance rates elucidated, and the concentrations of radiopharmaceuticals in the tumor vasculature for all four patients, with and without AAI determined (Fig 10). The reduction in peptide concentration in tumor microvasculature due to enhanced clearance rates of radiopharmaceuticals varies depending on each patient’s physiological characteristics. This prompts the question: why is AAI advantageous? What if the kidney were not the OAR, and other organs were the dose-limiting factors? Essentially, amino acids facilitate the clearance of a larger proportion of administered radiopharmaceuticals; however, an alternative approach could involve administering a lower dose of therapeutics instead of injecting amino acids.

To address the aforementioned inquiries, personalized RPT is implemented using the PBPK model, considering both the kidneys and salivary glands as OAR. Following the determination of the maximum safe activity to inject (as illustrated in Table 3), the PBPK model results are input to the CDR equation to compute the absorbed dose in the tumor. Administration of amino acids before treatment serves to reduce toxicity in the kidneys by competing with peptides for reabsorption, thereby increasing the clearance rate. Through personalized RPT and the utilization of the PBPK model, it becomes feasible to determine the maximum safe injected activity. Amino acid infusion creates an opportunity to administer higher doses of radiopharmaceuticals, consequently augmenting the absorbed dose in tumors. To analyze this effect, the kidney is initially assumed to be the dose-limiting organ, with a tolerated absorbed dose of 8 Gy during one cycle of RPT for all patients.

**Table 3:** Impact of amino acid administration on RPT Efficacy. Kidney and salivary gland organs are individually designated as organs-at-risk, determining the maximum safe injected activity. Subsequently, the absorbed dose to tumors is computed. Left column indicates the maximum possible activity that can be injected and the tumor absorbed dose when the kidney is the organ at risk, with and without amino acid administration. Right column indicates the maximum possible activity that can be injected and the tumor absorbed dose when the salivary gland is the organ at risk, with and without amino acid administration.

Patient	OAR = Kidney							OAR = Salivary Glands					
	Injected Activity (GBq)				Tumor Absorbed Dose (Gy)			Injected Activity (GBq)			Tumor Absorbed Dose (Gy)		
	With AAI	No AAI	Diff (RE%)		With AAI	No AAI	Diff (RE%)	With AAI	No AAI	Diff	With AAI	No AAI	Diff (RE%)
1	23.3	21.7	1.6 (7.3%)		75.6	70.6	5 (7.1%)	10	7.5	2.5 (33.3%)	44	43.8	0.2 (0.4%)
2	11.9	8.6	3.3 (38.4%)		151.2	140.5	10.7 (7.6%)	1.6	1.5	0.1 (6.6%)	31.9	31.6	0.3 (0.9%)
3	20.7	14	6.7 (47.8%)		10.9	9.3	1.6 (17.2%)	5.9	4.8	1.1 (22.9%)	5.8	5.6	0.2 (3.6%)
4	5.9	4.9	1 (20.4%)		52	50.8	1.2 (2.4%)	3.5	3.07	0.43 (14.0%)	35.8	35.6	0.2 (0.6%)
Average	15.45	12.3	3.15 (28.5%)		72.43	67.8	4.63 (8.6%)	5.25	4.22	1.03 (19.2%)	29.38	29.15	0.22 (1.4%)
SD	6.95	6.32	2.22 (15.7%)		51.03	47.45	3.81 (5.4%)	3.14	2.23	0.92 (9.9%)	14.29	14.29	0.04 (1.3%)
Min	5.9	4.9	1 (20.4%)		10.9	9.3	1.2 (2.4%)	1.6	1.5	0.1 (6.6%)	5.8	5.6	0.2 (3.6%)
Max	23.3	21.7	6.7 (47.8%)		151.2	140.5	10.7 (7.6%)	10	7.5	2.5 (33.3%)	44	43.8	0.3 (0.9%)
**P-value for Activity**				0.007 < 0.05									
**P-value for Absorbed Dose**				0.007 < 0.05									

With AAI: With amino acid infusion.

No AAI: Without amino acid infusion.

Diff: Difference – GBq for activity and Gy for absorbed dose (Relative Error (%)).

P-value for Activity: There is a significant difference in the administered activity with and without amino acid infusion.

P-value for Absorbed Dose: There is a significant difference in the absorbed dose with and without amino acid infusion.

Table 3 illustrates that considering kidney as OAR, the administered activity increased from 12.3 ± 6.32 MBq (mean ± SD) without AAI to 15.45 ± 6.95 MBq with AAI, showing an increase of 3.15 ± 2.22 MBq (28.5% ± 15.7%). Therefore, it is possible to increase the injected activity between 1 to 6.7 GBq for patients 1–4, while maintaining the kidney absorbed dose at its critical value. Consequently, the tumor absorbed dose increased from 67.8 ± 47.45 Gy without AAI to 72.43 ± 51.03 Gy with AAI, showing an increase of 4.63 ± 3.81 Gy (8.6% ± 5.4%). Therefore, the absorbed dose to tumors markedly increases between 1.2 to 10.7 Gy, representing a significant enhancement in RPT efficacy. The degree of increase in tumor absorbed dose varies among patients, contingent upon their specific physiological characteristics.

Due to the effect of amino acids in increasing the clearance rate, there is a reduction in absorbed dose to other organs, including the salivary gland, which is a positive outcome. However, a negative consequence is the decrease in the delivered dose to tumors. By considering the salivary gland as an OAR, it becomes feasible to augment the injected radiopharmaceuticals through amino acid infusion. Utilizing the PBPK model, the administered activity is increased up to a certain amount to achieve a critical absorbed dose to the salivary glands (7.5 Gy for each treatment cycle) based on personalized RPT.

For patients 1–4, and considering salivary glands as the OAR, the administered activity increased from 4.22 ± 2.23 MBq without AAI to 5.25 ± 3.14 MBq with AAI, with an increase of 1.03 ± 0.92 MBq (19.2% ± 9.9%), ranging between 0.1 to 2.5 GBq. The tumor absorbed dose showed a minimal increase from 29.15 ± 14.29 Gy without AAI to 29.38 ± 14.29 Gy with AAI, representing an increase of 0.22 ± 0.04 Gy (1.4% ± 1.3%), showing a mere elevation of approximately 0.2 Gy for all patients. These findings demonstrate that AAI consistently enhances administered activity and tumor absorbed dose, with more pronounced effects when kidneys are the OAR. Consequently, without amino acid infusion, it’s feasible to achieve nearly the same tumor absorbed dose by injecting a smaller amount of radiopharmaceuticals. These findings underscore the importance of determining OAR in personalized RPT to avoid wastage of radiopharmaceuticals and to select the most effective treatment strategy.

Given that only four data points were available for each condition, it is not reliable to perform statistical significance analysis for individual columns. Instead, a combined analysis was performed using the Wilcoxon Signed-Rank Test, comparing conditions with AAI to those without AAI, regardless of whether kidneys or salivary glands were the OAR. Results showed statistical significance for both administered activity and tumor absorbed dose, with P-values of 0.007 (< 0.05) for both parameters. This indicates that AAI significantly impacts administered activity and absorbed dose overall. Finally, our results, when compared with those from the PBPK model proposed by Kletting et al. [42], validate the effectiveness of our model in the realm of personalized RPT. This comparison underscores the alignment of our findings with established literature.

### 3.4. Sensitivity analysis

Among evaluated parameters, the range between 0.1 and 0.01 exhibited the highest normalized sensitivity indices (SIs) for f_tumor, k_tumor, and lambda_phy, indicating a heightened impact on the absorbed dose in this interval (Fig A in S3 Text). The mean ± standard deviation of the SIs for f_tumor, k_tumor, and lambda_phy were 0.771 ± 0.070, 0.752 ± 0.062, and 0.996 ± 0.001, respectively. These findings emphasize the critical influence of these parameters, particularly at lower scaling ranges, and underscore the need for precise estimation and control of these variables to optimize the modeling and understanding of radiopharmaceutical dynamics. Additionally, the range between 1 and 0.1 demonstrated the highest normalized SIs for the association rate (k_on) and PSMA binding site density (RD), with mean ± standard deviation values of 0.751 ± 0.018 and 0.891 ± 0.046, respectively. Conversely, the range between 10 and 1 exhibited the maximum SIs for the dissociation rate (k_off), internalization rate (lambda_int), and release rate from tumor cells (lambda_release). The mean ± standard deviation values for these parameters were -2.312 ± 0.168, 0.661 ± 0.197, and -1.849 ± 0.227, respectively. Finally, the range between 100 and 10 exhibited the smallest normalized SIs for most parameters, except for the dissociation rate (k_off) and release rate from tumor cells (lambda_release).

## 4. Discussion and conclusion

Radiopharmaceutical therapies stand out as one of the most promising treatment strategies in the oncological landscape. Their effectiveness lies in the ability to deliver targeted radiation directly to cancerous cells, minimizing damage to healthy tissue [132]. Such precision makes RPTs a preferred choice for various types of cancers, offering a potent tool in the fight against this debilitating disease. A key advancement in RPT lies in its potential for personalization [11,133]. Customizing treatment for each patient enables us to optimize the absorbed doses of tumors, thereby maximizing therapeutic efficacy while minimizing adverse effects on healthy tissues. This personalized approach represents a significant leap forward in cancer treatment, offering patients an effective therapeutic regimen. However, as we strive to enhance the efficacy of RPT, it is crucial to monitor the doses absorbed by healthy organs to prevent unintended toxicity. Effective management of dose-limiting healthy organs is essential to mitigate potential side effects and ensure patient safety throughout the treatment process [134,135]. Simultaneous imaging and therapy, known as theranostics, present a promising avenue in RPT. By integrating diagnostic imaging with therapeutic intervention, theranostic approaches enable real-time visualization of treatment response, facilitating timely adjustments and personalized interventions tailored to each patient’s unique needs [136,137].

To further refine RPT, researchers are turning to pharmacokinetic modeling, particularly PBPK models [95,96]. These models offer a sophisticated tool for estimating optimal injection amounts and treatment techniques, maximizing therapeutic efficacy while minimizing toxicity. However, PBPK models have inherent limitations, particularly in their temporal nature [138]. To address this challenge, CDR equations emerge as a promising solution. By incorporating CDR equations, researchers can better simulate the dynamic interplay of radiopharmaceutical distribution within the tumor microenvironment, offering a more comprehensive understanding of treatment dynamics [139–141]. Central to the application of CDR equations is the simulation of tumor microenvironment, including factors such as IFP distribution. Modeling the tumor microenvironment is a complex endeavor, often relying on mathematical simulations based on angiogenesis processes to generate realistic microvasculature networks [57,58,119]. Incorporating mathematical modeling of tumor microenvironment into a multiscale framework provides a powerful platform for analyzing RPT, its effects, and the various parameters influencing treatment outcomes. This integrated approach enables researchers to gain insights into the complex interactions between radiopharmaceuticals, tumor biology, and patient-specific factors, ultimately driving the development of more effective treatment strategies.

The incorporation of amino acid infusion represents a significant advancement in RPT, offering effects of reduced toxicity in vital organs, such as the kidney, while simultaneously enhancing the clearance rate of radiopharmaceuticals from the body [42,43,52,97]. This approach addresses a critical challenge in cancer treatment by minimizing the risk of adverse effects on healthy tissues while maximizing the therapeutic efficacy of the radiopharmaceuticals. By utilizing the previously outlined multiscale framework, we systematically assessed how amino acid infusion influences personalized RPT. This involved simulating the spatiotemporal distribution of radiopharmaceuticals within the tumor and examining the impact of IFP on RPT efficacy. While IFP and tumor heterogeneity are not routinely measured in clinical settings, we aim to investigate their effects on RPT using mathematical modeling. By simulating these factors within a computational framework, we seek to gain insights into their potential impact on treatment outcomes. Although these variables are not typically part of standard clinical practice, our study highlights their importance in optimizing personalized RPT, and future work may explore how these factors could be incorporated into clinical decision-making.

To achieve this, we employed a synthetic microvasculature model, allowing us to replicate the intricate dynamics of blood flow and drug dispersion within the tumor microenvironment with high precision and accuracy. This comprehensive approach allows for the exploration of how amino acid supplementation influences the distribution, clearance, and therapeutic efficacy of radiopharmaceuticals within the tumor microenvironment and throughout the body. In this study, we utilized data from five gamma camera images obtained from four patients with mCRPC to create virtual patient models [42]. These virtual patient models serve as powerful tools for simulating the response of diverse patient populations to RPT and amino acid infusion, enabling researchers to explore a wide range of treatment scenarios and identify optimal strategies for maximizing treatment efficacy.

Overall, the results reveal small differences between the calculated absorbed dose using concentrations derived from the PBPK model and those obtained from the CDR equations. These differences arise from the influence of IFP, which repels radiopharmaceuticals back to the microvasculature—a phenomenon not accounted for in the PBPK model. Furthermore, the impact of AAI is analyzed. Administering amino acids prior to treatment reduces kidney toxicity by competing with peptides for reabsorption, thereby enhancing clearance rates. By integrating personalized RPT and PBPK modeling, we can ascertain the maximum safe injected activity. This infusion presents an opportunity to administer higher radiopharmaceutical doses, thereby increasing tumor absorbed doses. Initially assuming the kidney as the dose-limiting organ, amino acid infusion allows for injected activity increases of 1 to 6.7 GBq while maintaining kidney absorbed doses at critical levels, leading to significant tumor absorbed dose enhancements of 1.2 to 10.7 Gy. Despite the positive effect of amino acids on reducing absorbed doses in organs like the salivary gland, there is a drawback of decreased tumor dose delivery. Considering the salivary gland as an OAR enables augmented radiopharmaceutical injections through amino acid infusion. PBPK modeling facilitates determining the administered activity needed to achieve critical kidney absorbed doses in personalized RPT, resulting in injected activity increases of 0.1 to 2.5 GBq for patients 1–4. This modest increase minimally impacts tumor absorbed doses, emphasizing the necessity of determining OARs in personalized RPT to optimize treatment efficacy and avoid radiopharmaceutical wastage. The administration of amino acids thus emerges as a promising technique when the kidney is under threat, showcasing potential benefits in managing renal function. However, it should be approached cautiously when the salivary gland is at risk, as it may not yield favorable outcomes. In such cases, it would be beneficial to implement additional protective techniques, such as the salivary gland cooling method, to safeguard the gland and minimize the risk of damage [142].

Despite the promising results, several limitations and assumptions need to be addressed in future work. The sample size of this study, limited to four patients, was constrained by the availability of patient data. Expanding the study to include more patient data, particularly from individuals with other tumor types and organs-at-risk, can provide broader insights and improve the generalizability of the findings. Furthermore, while this research employs a multiscale framework, it is possible to use only a whole-body PBPK model for practical clinical applications. This study primarily aims to enhance our understanding of the complexities and interactions within the tumor microenvironment.

The vascular network in this study is modeled in 2D, a methodological choice validated by our prior works [55,56] and supported by literature as accurately reflecting the structural characteristics of real tumor vasculature [143,144]. This approach balances computational efficiency with biological realism, allowing for the representation of key processes such as angiogenesis, nutrient diffusion, and therapeutic transport within a computationally tractable framework. Secomb et al. [145,146] emphasize that 2D models enable faster simulations and broader parameter exploration, making them particularly valuable for investigating fundamental biological mechanisms. Similarly, Anderson and Chaplain [57,58] demonstrated that 2D models can effectively capture critical phenomena like endothelial cell migration and capillary formation under specific conditions, supporting their use for analyzing general patterns of vascular growth and tumor progression. The 2D framework also significantly reduces the computational burden of simulating radiopharmaceutical distribution, while still providing meaningful insights into the cross-sectional dynamics of therapeutic transport. However, this simplification comes with limitations: the model does not capture the full three-dimensional complexity of tumor vasculature, including variations in vascular architecture and spatial heterogeneity, which may restrict its applicability to certain tumor types. These trade-offs highlight the need for further refinement when adapting the model to more complex tumor environments.

Given the significant structural heterogeneity exhibited by real tumors, we employed an inherently stochastic model to capture the randomness inherent in angiogenesis processes. However, we recognize that the current model may not be universally applicable to all tumor types without further refinement. For specific tumor types, additional considerations or modifications may be required to ensure its relevance and accuracy. For example, the model does not account for the heterogeneous distribution of vessel diameters, which could impact oxygen and nutrient delivery within the tumor microenvironment. Additionally, it assumes a relatively homogeneous tissue composition throughout the tumor, which limits its ability to represent the biological diversity of different regions, including necrotic cores, hypoxic zones, and areas of rapid proliferation. Although the model currently uses a 2D representation, it successfully captures the chaotic, multi-scale heterogeneity of tumor vasculature. Moreover, the methodology is adaptable to any vascular geometry, allowing for potential extension to both 2D and 3D configurations.

In our study, we modeled a stochastic microvascular network to simulate the angiogenic process, focusing on vessel sprouting over a 30-day period preceding the initiation of treatment. This approach captures the inherent randomness and variability of tumor angiogenesis, which is crucial for reflecting the biological heterogeneity observed in real tumors. We acknowledge that different simulations of vessel sprouting could influence the outcomes of radiopharmaceutical (RP) transport to the tumor. However, in this study, a single simulation of the angiogenic process was considered per patient, as our primary objective was to establish a foundational understanding of RP transport dynamics under a stochastic vascular framework. Incorporating multiple stochastic simulations per patient would allow for a more comprehensive analysis of how variations in vascular structure impact RP delivery and distribution within the tumor. We propose conducting multiple stochastic simulations per patient as an essential avenue for future research. This would help quantify the sensitivity of RP transport outcomes to variations in angiogenic patterns.

Finally, while this study provides valuable insights into radiopharmaceutical transport mechanisms, several aspects warrant further exploration in future research. First, although we conducted a local sensitivity analysis by varying one parameter at a time while keeping others constant, a more comprehensive global sensitivity analysis could provide a deeper understanding of the relative influence of all model parameters. This approach was beyond the scope of the current study but represents a promising avenue for future investigations. Second, future research could investigate tumor growth and changes in vasculature during treatment to enhance the understanding of their impact on radiopharmaceutical transport. In this study, we chose to focus on isolating and examining the mechanisms of radiopharmaceutical transport without the additional variability introduced by dynamic changes in tumor morphology and vascular architecture. Future studies could integrate these aspects to capture the interplay between tumor progression, vascular remodeling, and radiopharmaceutical distribution.

## Supporting information

S1 Text Fig A: Classification of compartments and their sub-compartments. (a) PSMA-positive organs, excluding kidney: Blood inflow: Arterial supply for all organs except liver. Liver: Triple inflow sources: arterial, splenic, and gastrointestinal. (b) Kidney: (c) PSMA-negative organs, excluding brain: Blood outflow: Venous drainage for all organs, except spleen and GI tract which drain into the liver. Note: Lung experiences venous inflow and arterial outflow. (d) Brain. Microsoft Office PowerPoint was used to create this figure. Image created using Servier Medical Art (https://smart.servier.com/), licensed under CC BY 4.0. Table A: All parameters and variables used in PBPK model.
(DOCX)

S2 TextFig A: The results of fitting the designed PBPK model to data obtained from γ-camera imaging for patient 1.Fig B: The results of fitting the designed PBPK model to data obtained from γ-camera imaging for patient 2. Fig C: The results of fitting the designed PBPK model to data obtained from γ-camera imaging for patient 3. Fig D: The results of fitting the designed PBPK model to data obtained from γ-camera imaging for patient 4. Table A: Parameters that used to calculate absorbed dose for OARs and tumors. Table B: S value for tumors and salivary glands based on their volumes. Table C: Estimated parameters obtained from fitting process for patient 1. Table D: Estimated parameters obtained from fitting process for patient 2. Table E: Estimated parameters obtained from fitting process for patient 3. Table F: Estimated parameters obtained from fitting process for patient 4.(DOCX)

S3 Text Fig A: Box plot showing the Normalized Sensitivity Indices (SI) Across Parameters and Scaling Ranges. The parameters—tumor serum flow density (f_tumor), permeability surface area product per unit mass (k_tumor), association rate (k_on), dissociation rate (k_off), internalization rate (lambda_int), physical decay (lambda_phy), PSMA binding site density (RD), and release rate from tumor cells (lambda_release)—were varied across four orders of magnitude (100, 10, 0.1, and 0.01). The mean and median of relative errors are displayed by red dash line and black solid line, respectively. Table A: The parameters used for computational results of the mathematical model. Table B: Absorbed dose due to presence of radiopharmaceuticals in tumor interstitial space, tumor cell receptors, and internalized within tumor cells) for four patients.
(DOCX)

S1 File Contains the COMSOL Multiphysics (.mph) file used to simulate the spatio-temporal distribution of the radiopharmaceutical described in the manuscript. The model includes geometry, parameters, and solver settings. Please note: this file requires COMSOL Multiphysics software (version 6.1 or later) to open and run.
(RAR)
