# Supplementary material for: Multi-scale computational modeling towards efficacy in radiopharmaceutical therapies while minimizing side effects: Modeling of amino acid infusion
Source: PLoS Comput Biol. 2025 Jul 16;21(7):e1013247. doi: 10.1371/journal.pcbi.1013247 (PMC12327665; doi:10.1371/journal.pcbi.1013247)
Supplement: S1 Text — (a) PSMA-positive organs, excluding kidney: Blood inflow: Arterial supply for all organs except liver. Liver: Triple inflow sources: arterial, splenic, and gastrointestinal. (b) Kidney: (c) PSMA-negative organs, excluding brain: Blood outflow: Venous drainage for all organs, except spleen and GI tract which drain into the liver. Note: Lung experiences venous inflow and arterial outflow. (d) Brain. Microsoft Office PowerPoint was used to create this figure. Image created using Servier Medical Art (https://smart.servier.com/), licensed under CC BY 4.0. Table A: All parameters and variables used in PBPK model. (DOCX) [file pcbi.1013247.s001.docx]

## PBPK Model equations

The following equations describe the transport of labeled (indexed with *) and unlabeled peptides via blood flow, extravasation, binding, internalization, degradation and release, excretion, and radioactive decay for virtual patients designed via PBPK model for mCRPC patients (Figure A in S1 Text). For therapy, the peptide was intravenously injected as a 10 min infusion. The variables of the model are defined in Table A in S1 Text.

### **Bound and internalized peptide:**

Tumor, salivary glands, kidneys, liver, spleen, GI and prostate:

Constraint for total PSMA binding sites R_0,i_

$R_{0, i}=R_{i}+RP_{i}+RP_{i}^{*}$ (S1)

Internalized peptide

$\begin{matrix} \frac{d}{dt}{P_{\mathrm{intern}}}_{i}=\lambda_{int,i}\cdot RP_{i}-\lambda_{release, i}\cdot{P_{\mathrm{intern}}}_{i}+\lambda_{phy}\cdot{{P^{*}}_{\mathrm{intern}}}_{i} \\ \frac{d}{dt}{{P^{*}}_{\mathrm{intern}}}_{i}=\lambda_{int,i}\cdot R{P^{*}}_{i}-\lambda_{release, i}\cdot{{P^{*}}_{\mathrm{intern}}}_{i}-\lambda_{phy}\cdot{{P^{*}}_{\mathrm{intern}}}_{i} \end{matrix}$ (S2)

Bound peptide on cell surface

$\begin{matrix} \frac{d}{dt}RP_{i}=k_{on}\cdot P_{i,int}\cdot\frac{R_{i}}{V_{i,int}}-(k_{off}+\lambda_{int,i})\cdot RP_{i}+\lambda_{phy}\cdot RP_{i}^{*} \\ \frac{d}{dt}R{P_{i}}^{*}=k_{on}\cdot{P_{i,int}}^{*}\cdot\frac{R_{i}}{V_{i,int}}-(k_{off}+\lambda_{int,i})\cdot R{P_{i}}^{*}-\lambda_{phy}\cdot RP_{i}^{*} \end{matrix}$ (S3)

### **Free peptide, vascular:**

Transcapillary extravasation is described by the permeability surface product (PS_i_) and the vascular (V_i,v_) and interstitial volumes (V_i,int_) of the pertaining tissue. Convection from the vascular to the interstitial space is neglected as the used peptide represents a rather small molecule [1].

All tissues except kidneys and lungs

$\begin{matrix} \frac{d}{dt}P_{i,v}=PS_{i}\left( \frac{P_{i,int}}{V_{i,int}}-\frac{P_{i,v}}{V_{i,v}} \right)+F_{i}\left( \frac{P_{A\mathrm{RT}}}{V_{ART}}-\frac{P_{i,v}}{V_{i,v}} \right)+\lambda_{phy}\cdot P_{i,v}^{*} \\ \frac{d}{dt}{P_{i,v}}^{*}= PS_{i}\left( \frac{{P_{i,int}^{*}}}{V_{i,int}}-\frac{{P_{i,v}^{*}}}{V_{i,v}} \right)+F_{i}\left( \frac{P_{A\mathrm{RT}}^{*}}{V_{ART}}-\frac{P_{i,v}^{*}}{V_{i,v}} \right)-\lambda_{phy}\cdot P_{i,v}^{*} \end{matrix}$ (S4)

For brain PS = 0

Lungs

$\begin{matrix} \frac{d}{dt}P_{LU,v}=PS_{LU}\left( \frac{P_{LU,int}}{V_{LU,int}}-\frac{P_{LU,v}}{V_{LU,v}} \right)+F\left( \frac{P_{V\mathrm{EN}}}{V_{VEN}}-\frac{P_{LU,v}}{V_{LU,v}} \right)+\lambda_{phy}\cdot P_{LU,v}^{*} \\ \frac{d}{dt}{P_{LU,v}}^{*}= PS_{LU}\left( \frac{{P_{LU,int}}^{*}}{V_{LU,int}}-\frac{{P_{LU,v}}^{*}}{V_{LU,v}} \right)+F\left( \frac{P_{V\mathrm{EN}}}{V_{VEN}}-\frac{{P_{LU,v}}^{*}}{V_{LU,v}} \right)-\lambda_{phy}\cdot P_{LU,v}^{*} \end{matrix}$ (S5)

Kidneys

$\begin{matrix} \frac{d}{dt}P_{K,v}=-\frac{P_{K,v}}{V_{K,v}}\cdot(F_{fil}+F_{K})+\frac{F_{K}}{V_{ART}}\cdot P_{A\mathrm{RT}}+\frac{P_{intra,K}}{V_{intra,K}}\cdot(F_{fil}-F_{ex})+\lambda_{phy}\cdot P_{K,v}^{*} \\ \frac{d}{dt}P_{K,v}^{*}=-\frac{P_{K,v}^{*}}{V_{K,v}}\cdot(F_{fil}+F_{K})+\frac{F_{K}}{V_{ART}}\cdot P_{A\mathrm{RT}}^{*}+\frac{{P_{intra,K}^{*}}}{V_{intra,K}}\cdot(F_{fil}-F_{ex})-\lambda_{phy}\cdot P_{K,v}^{*} \end{matrix}$ (S6)

Veins

$\begin{matrix} \frac{d}{dt}P_{V\mathrm{EN}}=-k_{\Pr}\cdot P_{V\mathrm{EN}}+\sum\frac{F_{i}}{V_{i}}P_{i,v}-\frac{F_{M}}{V_{M}}P_{M,v}-\frac{F_{GI}}{V_{GI}}P_{GI,v}+\frac{F_{M}+F_{GI}}{V_{L}}P_{L,v}+\lambda_{phy}\cdot P_{V\mathrm{EN}}^{*} \\ \frac{d}{dt}P_{VEN}^{*}=-k_{\Pr}\cdot P_{VEN}^{*}+\sum\frac{F_{i}}{V_{i}}{P_{i,v}^{*}}-\frac{F_{M}}{V_{M}}P_{M,v}^{*}-\frac{F_{GI}}{V_{GI}}P_{GI,v}^{*}+\frac{F_{M}+F_{GI}}{V_{L}}P_{L,v}^{*}-\lambda_{phy}\cdot P_{V\mathrm{EN}}^{*} \end{matrix}$ (S7)

Arteries

$\begin{matrix} \frac{d}{dt}P_{A\mathrm{RT}}=-\sum\frac{F_{i}}{V_{ART}}\cdot P_{i,v}+\frac{F}{V_{LU,v}}\cdot P_{LU,v}+\lambda_{phy}\cdot P_{ART}^{*} \\ \frac{d}{dt}P_{ART}^{*}=-\sum\frac{F_{i}}{V_{ART}}\cdot P_{i,v}+\frac{F}{V_{LU,v}}\cdot P_{LU,v}-\lambda_{phy}\cdot P_{ART}^{*} \end{matrix}$ (S8)

### **Free peptide, interstitial spaces:**

Kidneys:

$\begin{matrix} \frac{d}{dt}P_{K,int}=-k_{on}\cdot P_{K,int}\cdot\frac{R_{K}}{V_{K,int}}+k_{off}\cdot RP_{K}+F_{fil}\left( \frac{P_{K,v}}{V_{K,v}}-\frac{P_{K,int}}{V_{K,int}} \right)+\lambda_{phy}\cdot P_{K,int}^{*} \\ \frac{d}{dt}P_{K,int}^{*}=-k_{on}\cdot P_{K,int}^{*}\cdot\frac{R_{K}}{V_{K,int}}+k_{off}\cdot RP_{K}^{*}+F_{fil}\left( \frac{P_{K,v}^{*}}{V_{K,v}}-\frac{P_{K,int}^{*}}{V_{K,int}} \right)-\lambda_{phy}\cdot P_{K,int}^{*} \end{matrix}$ (S9)

Muscle, red marrow, skin, lungs, adipose tissue, heart, bone, rest and brain (PS = 0):

$\begin{matrix} \frac{d}{dt}P_{i,int}=PS_{i}\left( \frac{P_{i,v}}{V_{i,v}}-\frac{P_{i,int}}{V_{i,int}} \right)+\lambda_{phy}\cdot P_{i,int}^{*} \\ \frac{d}{dt}P_{i,int}^{*}=PS_{i}\left( \frac{P_{i,v}^{*}}{V_{i,v}}-\frac{P_{i,int}^{*}}{V_{i,int}} \right)-\lambda_{phy}\cdot P_{i,int}^{*} \end{matrix}$ (S10)

Tumor, salivary glands, kidneys, liver, spleen, GI and prostate:

$\begin{matrix} \frac{d}{dt}P_{i,int}=-k_{on}\cdot P_{i,int}\cdot\frac{R_{i}}{V_{i,int}}+k_{off}\cdot RP_{i}+PS_{i}\left( \frac{P_{i,v}}{V_{i,v}}-\frac{P_{i,int}}{V_{i,int}} \right)+\lambda_{phy}\cdot P_{i,int}^{*} \\ \frac{d}{dt}P_{i,int}^{*}=-k_{on}\cdot P_{i,int}^{*}\cdot\frac{R_{i}}{V_{i,int}}+k_{off}\cdot R{P_{i}}^{*}+PS_{i}\left( \frac{P_{i,v}^{*}}{V_{i,v}}-\frac{P_{i,int}^{*}}{V_{i,int}} \right)-\lambda_{phy}\cdot P_{i,int}^{*} \end{matrix}$ (S11)

### **Further equations:**

Peptide in kidney cells (unspecific)

$\begin{matrix} \frac{d}{dt}P_{intra,K}=\frac{P_{int,K}}{V_{int, K}}\cdot(F_{fil}-F_{ex})-\frac{P_{intra,K}}{V_{intra,K}}\cdot(F_{fil}-F_{ex})+\lambda_{phy}\cdot P_{intra,K}^{*} \\ \frac{d}{dt}P_{intra,K}^{*}=\frac{{P_{int,K}^{*}}}{V_{int, K}}\cdot(F_{fil}-F_{ex})-\frac{P_{intra,K}^{*}}{V_{intra,K}}\cdot(F_{fil}-F_{ex})-\lambda_{phy}\cdot P_{intra,K}^{*} \end{matrix}$ (S12)

Bound to protein

$\begin{matrix} \frac{d}{dt}PRP=k_{PR}\cdot P_{V\mathrm{EN}}+\lambda_{phy}\cdot PRP^{*} \\ \frac{d}{dt}PRP^{*}=k_{PR}\cdot P_{VEN}^{*}-\lambda_{phy}\cdot PRP^{*} \end{matrix}$ (S13)

## Mechanism and Structure of PBPK Model

Nine compartments are allocated for PSMA-positive organs including salivary glands, liver, spleen, GI, prostate, two tumor lesions were explicitly modeled by two compartments and all other tumor lesions were merged into tumor REST compartment (Figure A in S1 text). All these compartments consist of 4 sub-compartments which modeled the vascular and interstitial space of organs, the process of binding peptide to receptors and internalization, but kidney modeled with 5 sub-compartments (Figure A in S1 text). For the kidney which is another PSMA-positive organ, unspecific binding and all mechanisms pertaining to clearance were also included. All unspecific binding mechanisms were illustrated with the fractional flow of peptide into and out of the cells back to the serum compartment. Amino acids were administered to block unspecific uptake which substantially decreases the nonspecific uptake.

The model also contains 9 other compartments to represent adipose, lungs, bone, Red marrow, skin, heart, muscle and one for rest of the body as PSMA-negative organs with 2 sub-compartments (Figure A in S1 text). Another PSMA-negative organ is brain that only has 1 sub-compartment (Figure A in S1 text). 2 compartments are dedicated arteries and veins and one compartment contains peptide bound to serum protein. As the fraction of bound peptide to proteins is small compared to the total amount and to reduce complexity, only the veins were connected to this compartment.

Due to the fact that some parameters of the model have an unknown value, the pharmacokinetic information of OARs and tumors are needed to estimate these parameters. By determining some points (4 or 5) on the TAC of kidney, salivary glands, tumors and whole body which are obtained by imaging, and by fitting the model to these data, the desired parameters can be estimated. Since the radionuclide used is ^177^Lu, imaging has been done at one cycle of treatment (0.5 h, 2h, 1D, 2D and 3D after infusion) using $\gamma$-cameras. Also, λ_K, release_ assumed to be equal to λ_K, NT_ which is release rate of all normal tissues. Also no changes in tumor perfusion, receptor expression and tumor volume was considered during therapy.

Additionally, the model assumed that the released portion or free ^177^Lu were directly cleared from the body. The individually measured glomerular filtration rates scaled to PSMA molecule size because the macromolecular sieving coefficient of 51Cr-EDTA is smaller than for PSMA. Internalization rates of all organs assumed to be 0.001 min^-1^. All parameters are presented in supplementary materials (Table A in S1 Text).

| 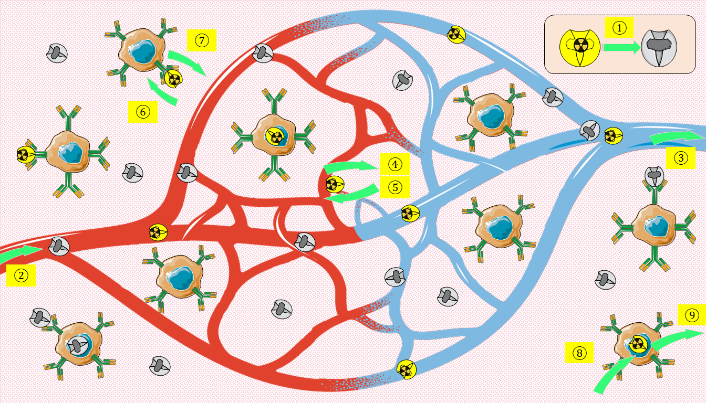  (a) | | | | | | 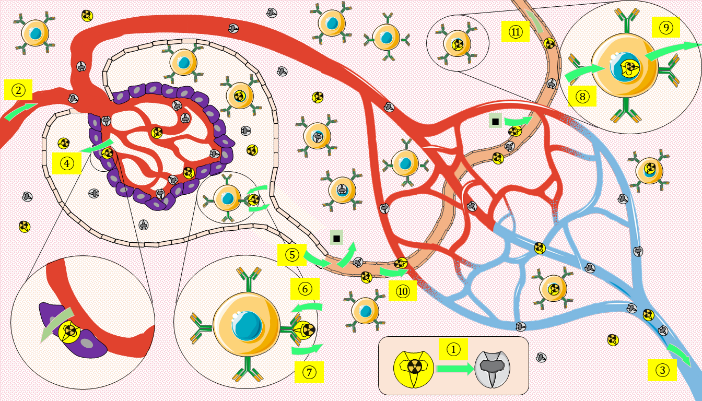  (b) | | | | |
| --- | --- | --- | --- | --- | --- | --- | --- | --- | --- | --- |
| 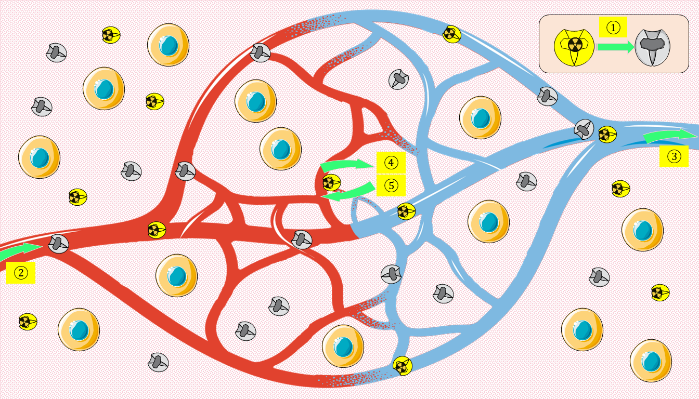  (c) | | | | | | 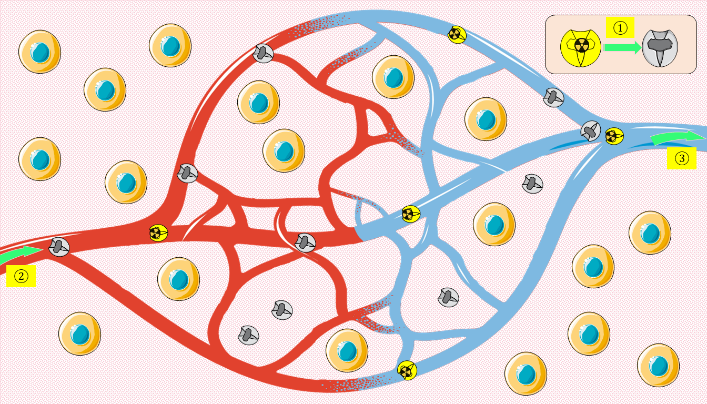  (d) | | | | |
| **symbol** | 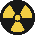 |  |  |  |  |  |  |  |  |  |
| **definition** | Labeled  peptides | Unlabeled  peptides | PSMA  Reseptors | Cells | Glomerular   basement cells | Veins  (Vascular Space) | Arteries  (Vascular Spcace) | Bowman’s spcae  (Interstital Spcace) | Interstital Spcace | Intratubular space |

Figure A in S1 Text: Classification of compartments and their sub-compartments. (a) PSMA-positive organs, excluding kidney: Blood inflow: Arterial supply for all organs except liver. Liver: Triple inflow sources: arterial, splenic, and gastrointestinal. (b) Kidney: (c) PSMA-negative organs, excluding brain: Blood outflow: Venous drainage for all organs, except spleen and GI tract which drain into the liver. Note: Lung experiences venous inflow and arterial outflow. (d) Brain. Microsoft Office PowerPoint was used to create this figure. Image created using Servier Medical Art (https://smart.servier.com/), licensed under CC BY 4.0.

Physical decay of radionuclide convert labeled peptides to unlabeled peptides in all compartments and sub-compartments with a rate of: 1) $\lambda_{phy}$

For all organs, except lungs and liver, flow enters into the vascular space of organ from arteries with this rate: 2) $\frac{F_{i}}{V_{art}}$

For liver flow enters to vascular space from arteries, spleen and GI: 2) $\frac{F_{liver}}{V_{art}}+\frac{F_{spleen}}{V_{spleen,v}}+\frac{F_{GI}}{V_{GI,v}}$

But for lungs in enters from veins: 2) $\frac{F_{total}}{V_{veins}}$

For all organs, except lungs, liver, spleen and GI, flow exit from vascular space to veins with this rate: 3) $\frac{F_{i,v}}{V_{i,v}}$

For spleen and GI flow exit from vascular space to liver with a rate of: 3) $\frac{F_{i,v}}{V_{i,v}}$

For liver flow exit from vascular space to veins with the rate of: 3) $\frac{(F_{liver}+F_{spleen+}F_{GI})}{V_{liver,v}}$

But for lungs flow exit from vascular space to arteries with this rate: 3) $\frac{F_{total}}{V_{lungs,v}}$

After entering the vascular space, peptides diffuse into interstitial space. It also back into vascular space, for all organs (except kidney) entering and exiting rate is as follows respectively: 4) $\frac{{PS}_{i,v}}{V_{i,v}}$ 5) $\frac{{PS}_{i,v}}{V_{i,int}}$

For kidney, after entering the flow into vascular space of bowman’s capsule, peptides is filtered passing through endothelial cells, basement membrane, and podocytes cells into bowman’s space (extraglomerular which is interstitial space) with the rate of: 4) $\frac{GFR.\varphi}{V_{kidney,v}}$. Therefore, after entering of peptides into extraglomerular space the peptides flow into intratubular space with the rate of: 5) $\frac{GFR.\varphi.(1-f_{ex})}{V_{kidney,cell}}$. Peptides in interstitial space does not back directly to vascular space.

Peptides transport between intratubular space and extravascular space of the kidney (represented with
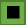
 in Figure A in S1 Text).

In brain peptides are in vascular space and do not diffuse into interstitial space.

In PSMA positive organs, peptides which are in interstitial space, bind to PSMA receptors with a non-linear rate: 6) $k_{on,nonlinear}=\frac{k_{on}}{V_{i,int}}.(R_{0,i}-{RP}_{i}-{RP}_{i}^{*})$. Peptides also disacociated from receptors and back to interstitial space with a rate of: 7) $k_{off}$

Peptides that bound to receptors, internalized into cells with a rate of: 8) $\lambda_{i, internal}$. They also release from cell by: 9) $\lambda_{i, release}$

Kidney cells also have unspecific uptake with a rate of 10) $\frac{GFR.\varphi.(1-f_{ex})}{V_{kidney,int}}$ back to vascular space. They also cleared from body through intratubular space with a rate of: 11) $\frac{GFR.\varphi.f_{ex}}{V_{kidney,int}}$

Table A in S1 Text: All parameters and variables used in PBPK model.

| **Variable** | | **Value** | **Unit** | **Source** |
| --- | --- | --- | --- | --- |
| k_on_ | association rate | 0.046 | l·nmol^-1^·min^-1^ | [2]^a^ |
| K_D_ | dissociation constant | 1 or 8 | nmol·l^-1^ | [2]^a^[3] |
| k_off_ | dissociation rate | K_D_· k_on_ | min^-1^ |  |
| λ_phy_ | physical decay ^177^Lu | 7.15·10^-5^ | min^-1^ |  |
| BW | body weight | measured | kg |  |
| BH | body height | measured | cm |  |
| H | hematocrit | measured | unity |  |
| F | flow total serum | V_P_ ·1.23/min^b^ | l·min^-1^ | [4] |
| V_P_ | volume of total body serum | 2.8·(1- H)·BSA·(l·m^-2^) | l | [5] |
| BSA | body surface area | 0.007184·BH^0.725^·BW^0.425^ | m^2^ | [5] |
| ρ | assumed density for all organs and tumor | 1 ml ≙ 1 g |  |  |
|  |  |  |  |  |
| **Tumor** | | | | |
| V_TU,total_ | total volume of tumor 1 and 2 | measured | l |  |
| V_TU, int_ | interstitial space of tumor | v_TU,int_·V_TU,total_ | l |  |
| V_TU, v_ | vascular space of tumor | v_TU,v_·V_TU,total_ | l |  |
| V_TU,Rest, int_ | interstitial space of tumor remainder | v_TU,int_·R_TU,Rest, 0_/[R_TU,Rest, 0_] | l |  |
| V_TU,Rest, v_ | vascular space of tumor remainder | v_TU,v_·R_TU,Rest, 0_/[R_TU,Rest, 0_] | l |  |
| v_TU,int_ | interstitial space fraction of total tumor | 0.38 | unity | [6] |
| v_TU,v_ | vascular (serum) fraction of total tumor | 0.05·(1-H) | unity | [7] |
| F_TU_ | serum flow tumor | f_TU_·V_TU,total_ | l·min^-1^ |  |
| F_TU,Rest_ | serum flow tumor remainder | f_TU_·R_TU,Rest, 0_/[R_TU,Rest, 0_] | ml·min^-1^ |  |
| f_TU_ | serum flow density tumor | 0.5 | ml·min^-1^·g^-1^ | [7, 8] |
| PS_TU_ | permeability surface area product tumor | k_TU_·V_TU,total_ | ml·min^-1^ |  |
| PS_TU,Rest_ | permeability surface area product tumor remainder | k_TU_·R_TU,Rest, 0_/[R_TU,Rest, 0_] | ml·min^-1^ |  |
| k_TU_ | permeability surface area product per unit mass (scaled for molecule size of PSMA I&T) | 0.6 (maximal value from (6)) | ml·min^-1^·g^-1^ | [7] |
| [R_TU,0_] | PSMA binding site density | fitted | nmol·l^-1^ |  |
| [R_TU,Rest, 0_] | PSMA binding site density tumor remainder | 266 | nmol·l^-1^ | [9] |
| R_TU,0_ | PSMA binding sites number | [R_TU,0_]·V_TU,total_ | nmol |  |
| R_TU,Rest, 0_ | PSMA binding sites number tumor remainder ^c^ | P1-5: 13 ; 2.6 ;13 ; 2.6 ;13 | nmol |  |
| λ_TU,int_ | internalisation rate tumor | 0.001 | min^-1^ | [10] |
| λ_TU,release_ | release rate tumor | fitted | min^-1^ |  |
| λ_TU,Rest,int_ | internalisation rate tumor remainder | 0.001 | min^-1^ | [10] |
| λ_TU,Rest,release_ | release rate tumor remainder | (λ_TU,1,release_ + λ_TU,2,release_)/2 | min^-1^ |  |
|  |  |  |  |  |
| **Liver , spleen and kidneys** | | | | |
| V_L,total_ | volume total liver | 1.91 | l | [11] |
| V_S,total_ | volume total spleen | 0.183 | l |  |
| V_K,total_ | volume total kidneys | measured | l |  |
| V_i,v_ | vascular (serum) volume organ liver, spleen, kidneys | V_i,total_·v_i,v_ | l |  |
| V_i,int_ | interstitial volume liver, spleen, kidneys | V_i,total_·v_i,int_ | l |  |
| V_K, intra_ | volume intracellular kidneys | (V_K,total_ - V_K,int_ - V_K,v_)·2/3^d^ | l |  |
| v_L,v_ | vascular (serum) fraction liver | 0.085 | unity | [12] |
| v_S,v_ | vascular (serum) fraction spleen | 0.12 | unity | [12] |
| v_K,v_ | vascular (serum) fraction kidneys | 0.055 | unity | [12] |
| v_L,int_ | interstitial fraction liver | 0.2 | unity | [12] |
| v_S,int_ | interstitial fraction spleen | 0.2 | unity | [12] |
| v_K,int_ | interstitial fraction kidneys | 0.15 | unity | [12] |
| F_L_ | serum flow liver arterial | 0.065·F | l·min^-1^ | [4] |
| F_S_ | serum flow spleen | 0.03·F | l·min^-1^ | [4] |
| F_K_ | serum flow kidneys | 0.19·F | l·min^-1^ | [4] |
| φ | ratio of sieving coefficients | Θ_PSMA I&T_ / Θ_Cr-51-EDTA_= 0.66 | unity | [13] |
| GFR | glomerular filtration rate with ^51^Cr-EDTA | TER_measured_/3·20 l /15 l | l·min^-1^ | [14] |
| TER | tubular extraction rate ^99m^Tc-MAG3 | measured | l·min^-1^ |  |
| F_fil_ | filtration | GFR·φ ^e^ | l·min^-1^ |  |
| F_ex_ | excretion | F_fil_·f_ex_ | l·min^-1^ |  |
| f_ex_ | excretion fraction | 0.96 | unity | [15] |
| k_L_ | permeability surface area product per unit mass for liver | k_MUS_·100 | ml·min^-1^·g^-1^ | [16] |
| k_S_ | permeability surface area product per unit  mass for spleen | k_L_ (due to similar capillary structure) | ml·min^-1^·g^-1^ |  |
| [R_L,0_] | binding site density liver | [R_PRO,0_]·0.05 | nmol·l^-1^ | [17] |
| [R_S,0_] | binding site density spleen | [R_PRO,0_]·0.02 | nmol·l^-1^ | [17] |
| [R_K,0_] | binding site density kidneys | fitted | nmol·l^-1^ |  |
| λ_L, int_ | internalization rate PSMA liver | λ_TU, int_ | min^-1^ | [18] ^f^ |
| λ_S, int_ | internalization rate PSMA spleen | λ_TU, int_ | min^-1^ | [18] ^f^ |
| λ_K, int_ | internalization rate PSMA kidneys | λ_TU, int_ | min^-1^ | [18] ^f^ |
| λ_L,release_ | release rate liver | λ_K,release_ | min^-1^ | [15, 19] |
| λ_S,release_ | release rate spleen | λ_K,release_ | min^-1^ | [15] |
| λ_K,release_ | release rate kidneys | fitted | min^-1^ |  |
|  |  |  |  |  |
| **Other organs** | | | | |
|  |  |  |  |  |
| V_PRO,total_ | volume total prostate | 0.016·BW/71 | l | [20] |
| V_LU,total_ | volume total lungs | 1·BW/71 | l | [20] |
| V_SAL,total_ | volume total salivary glands  = left plus right parotid gland | measured | l |  |
| V_MUS,total_ | volume total muscles | 30.078·BW/71 | l | [20] |
| V_GI,total_ | volume total GI + pancreas | (0.385+0.548+0.104+0.15)·BW/71 | l | [20] |
| V_SKIN,total_ | volume total skin | 3.408·BW/71 | l | [20] |
| V_ADI,total_ | volume total adipose tissue | 13.465·BW/71 | l |  |
| V_RM,total_ | volume total red marrow | 1.1·BW/71 | l | [20] |
| V_BONE,total_ | volume total bone without red marrow | 10.165·BW/71 - V_RM,total_ | l | [20] |
| V_HRT,total_ | volume total heart | 0.341·BW/71 | l | [20] |
| V_BR,total_ | volume total brain | 1.45·BW/71 | l | [20] |
| V_BW_ | volume of total body based on BW | 1 ml ≙ 1 g | l |  |
| V_REST,total_ | volume of rest body  i = all organs except tumor | $V_{BW}-\sum_{i} V_{i,total}$ | l |  |
| V_PRO,v_ | vascular volume prostate | 0.004 ·(1-H)·V_PRO,total_ | l | [6] |
| V_LU,v_ | vascular (serum) volume lungs | 0.105·V_P_ | l | [4] |
| V_SAL,v_ | vascular (serum) volume salivary glands | 0.03·(1-H)·V_SAL,total_ | l | [21] |
| V_MUS,v_ | vascular (serum) volume muscles | 0.14·V_P_ | l | [4] |
| V_GI,v_ | vascular (serum) volume GI+ pancreas | 0.076·V_P_ | l | [4] |
| V_SKIN,v_ | vascular(serum) volume skin | 0.03·V_P_ | l | [4] |
| V_ADI,v_ | vascular(serum) volume adipose tissue | 0.05·V_P_ | l | [4] |
| V_RM,v_ | vascular(serum) volume red marrow | 0.04·V_P_ | l | [4] |
| V_BONE,v_ | vascular volume bone without red marrow | 0.07·V_P_ -V_RM_ | l | [4] |
| V_HRT,v_ | vascular (serum) volume heart (supply) | 0.01·V_P_ | l | [4] |
| V_BR,v_ | vascular(serum) volume brain | 0.012·V_P_ | l | [4] |
| V_REST,v_ | serum volume rest i = all organs except tumor | $V_{P}-\sum_{i} V_{i,v}$ | l |  |
| V_ART_ | arterial serum plus ½ serum content of heart | 0.06·V_P_ + 0.045·V_P_ | l | [4] |
| V_VENES_ | venous serum plus ½ serum content of heart | 0.18·V_P_ + 0.045·V_P_ | l | [4] |
| V_PRO,int_ | interstitial fraction prostate | 0.25·V_PRO,total_ | l | [6] |
| V_LU,int_ | interstitial fraction lungs | V_LU,v_·α_LU_ | l |  |
| V_SAL,int_ | interstitial fraction salivary glands | 0.23·V_SAL,total_ | l | [21] |
| V_MUS,int_ | interstitial fraction muscles | V_MUS,v_·α_MUS_ | l |  |
| V_GI,int_ | interstitial fraction GI+ pancreas | V_GI,v_·α_GI_ | l |  |
| V_SKIN,int_ | interstitial fraction skin | V_SKIN,v_·α_SKIN_ | l |  |
| V_ADI,int_ | interstitial fraction adipose tissue | V_ADI,v_·α_ADI_ | l |  |
| V_RM,int_ | interstitial fraction red marrow | V_RM,v_·α_RM_ | l |  |
| V_BONE,int_ | interstitial fraction bone without red marrow | V_BONE,v_·α_BONE_ | l |  |
| V_HRT,int_ | interstitial fraction heart | V_HRT,v_·α_HRT_ | l |  |
| V_REST,int_ | volume of rest body | V_REST,v_·α_REST_ | l |  |
| α_MUS_ | ratio of interstitial to vascular volume average man | V_MUS,int_/V_MUS,v_ = 5.9 | unity | [12] |
| α_GI_ | ratio of interstitial to vascular volume average man | V_GI,int_/ V_GI,v_ = 8.8 | unity | [12] |
| α_SKIN_ | ratio of interstitial to vascular volume average man | V_SKIN,int_/ V_SKIN,v_ = 8.9 | unity | [12] |
| α_ADI_ | ratio of interstitial to vascular volume average man | V_ADI,int_/ V_ADI,v_ = 15. 5 | unity | [12] |
| α_RM_ | ratio of interstitial to vascular volume average man | V_RM,int_/ V_RM,v_ = 3.7 | unity | [12] |
| α_HRT_ | ratio of interstitial to vascular volume average man | V_HRT,int_/ V_HRT,v_ = 3.7 | unity | [12] |
| α_LU_ | ratio of interstitial to vascular volume average man | V_LU,int_/ V_LU,v_ = 5.5 | unity | [12] |
| α_BONE_ | ratio of interstitial to vascular volume average man | V_BONE,int_/ V_BONE,v_ = 8.4 | unity | [12] |
| α_REST_ | ratio of interstitial to vascular volume average man | V_REST,int_/ V_REST,v_ = 4.1 | unity | [12] |
|  |  |  |  |  |
| f_PRO_ | serum flow density prostate | 0.18·(1-H) | ml·min^-1^·g^-1^ | [6] |
| F_PRO_ | total serum flow to prostate | f_PRO_·V_PRO,total_ | ml·min^-1^ |  |
| f_SAL_ | serum flow density salivary glands | Fitted | ml·min^-1^·g^-1^ |  |
| F_SAL_ | total serum flow to salivary glands | f_SAL_·V_SAL,total_ | ml·min^-1^ |  |
| F_LU_ | total serum flow lungs | F | ml·min^-1^ | [4] |
| F_MUS_ | total serum flow to muscle | 0.17·F | ml·min^-1^ | [4] |
| F_GI_ | total serum flow to GI+ pancreas | 0.16·F | ml·min^-1^ | [4] |
| F_SKIN_ | total serum flow to skin | 0.05·F | ml·min^-1^ | [4] |
| F_ADI_ | total serum flow to adipose | 0.05·F | ml·min^-1^ | [4] |
| F_RM_ | total serum flow to red marrow (RM) | 0.03·F | ml·min^-1^ | [4] |
| F_BONE_ | total serum flow to bone (without RM) | 0.05·F | ml·min^-1^ | [4] |
| F_HRT_ | total serum flow to heart | 0.04·F | ml·min^-1^ | [4] |
| F_BR_ | total serum flow to brain | 0.12·F | ml·min^-1^ | [4] |
| F_REST_ | i = all organs except tumor | $F-\sum_{i} F_{i}$ | ml·min^-1^ |  |
| F_TOTAL_ |  | $F+F_{TU,1}+F_{TU,2}+F_{TU,REST}$ | ml·min^-1^ |  |
| PS_i_ | permeability surface area product | k_i_·V_i, total_ | ml·min^-1^ |  |
| k_PRO_ | permeability surface area product per unit mass (scaled for molecule size of PSMA I&T) for prostate | 0.1 | ml·min^-1^·g^-1^ | [6] |
| k_LU_ | permeability surface area product per unit mass for lungs | k_MUS_·100 | ml·min^-1^·g^-1^ | [16] |
| k_SAL_ | permeability surface area product per unit mass for salivary glands | k_MUS_·100 | ml·min^-1^·g^-1^ | [22] for submand. glands |
| k_MUS_ | permeability surface area product per unit mass for muscle | 0.02 | ml·min^-1^·g^-1^ | [16] |
| k_GI_ | permeability surface area product per unit mass for GI and pancreas | 0.02  (assumed to similar to muscle) | ml·min^-1^·g^-1^ |  |
| k_SKIN_ | permeability surface area product per unit mass for skin | 0.02  (assumed to similar to muscle) | ml·min^-1^·g^-1^ |  |
| k_ADI_ | permeability surface area product per unit mass for adipose | 0.02  (assumed to similar to muscle) | ml·min^-1^·g^-1^ |  |
| k_RM_ | permeability surface area product per unit mass for red marrow | k_L_(assumed to similar to liver) | ml·min^-1^·g^-1^ |  |
| k_HRT_ | permeability surface area product per unit mass for heart | 0.02  (assumed to similar to muscle) | ml·min^-1^·g^-1^ |  |
| k_BONE_ | permeability surface area product per unit mass for bone | 0.02  (assumed to similar to muscle) | ml·min^-1^·g^-1^ |  |
| k_REST_ | permeability surface area product per unit mass for rest | 0.02  (assumed to similar to muscle) | ml·min^-1^·g^-1^ |  |
| [R_PRO,0_] | binding site density prostate | [R_TU,Rest,0_] ·0.1 | nmol l^-1^ | [23] |
| [R_SAL,0_] | binding site density salivary glands | fitted | nmol l^-1^ |  |
| [R_GI,0_] | binding site density GI + pancreas | [R_PRO,0_]·0.06 | nmol l^-1^ | [17] |
| λ_NT,int_ | internalization rate for normal tissue | λ_TU,int_ | min^-1^ | [18]^f^ |
| λ_NT,release_ | degradation and release normal tissue (except salivary glands) | λ_K,release_ | min^-1^ |  |
| λ_SAL,release_ | degradation and release salivary glands | fitted | min^-1^ |  |
| R | binding sites free |  | nmol |  |
| R_i_,_0_ | binding sites total number of organ i | [R_i_,_0_]·V_i,total_ | nmol |  |
| [R_i_,_0_] | binding site density of organ i |  | nmol l^-1^ |  |
| RP_i_ | peptide bound |  | nmol |  |
| PRP | peptide bound to serum protein |  | nmol |  |
| k_PR_ | binding rate peptide to serum | 4.7·10^-4^ | min^-1^ | [10] |
| P_intern_ | peptide internalized |  | nmol |  |
| P_i,v_ | peptide free vascular |  | nmol |  |
| P_i,int_ | peptide free interstitial |  | nmol |  |
| P_K,intra_ | peptide interacellular kidneys |  | nmol |  |
| P_inj_ | injected amount of unlabeled peptide | P1-5: 139; 91; 81; 67;294 | nmol |  |
| P*_inj_ | injected amount of labeled peptide | P1-5: 8.4;7.5; 7.5;7.5;7.8 | nmol |  |

^a^Mean values from all measured (Biacore) ligands.

^b^For the average normal adult (blood) F = 6500 ml/min and V = 5300 ml. Therefore, a factor of 1.23 was assigned to account for the changes in total serum flow due to volume changes.

^c^Using the assumption of 266 nmol·l^-1^ binding site density [9], 10^12^ cells per liter and 10 ml or 50 ml addition tumor volume.

^d^It is assumed that 2/3 of the total intracellular volume of the kidneys is represented by the proximal tubular cells

^e^Scaling of GFR due to different molecular sizes

^f^Antunes et al. do not report values for ^177^Lu. However, for the investigated ligands labeled with ^111^In, kidney and tumor cells showed similar internalization in many cases.

**References**

1. Rippe B, Haraldsson B. Fluid and protein fluxes across small and large pores in the microvasculature. Application of two-pore equations. acta Physiol Scand. 1987;131(3):411-28.

2. Winter G, Drescher A, Baur B, Solbach C, Reske SN, Beer AJ. Comparative analysis of chelator-modified peptides for imaging of prostate carcinoma. Annual Congress EANM. 2014;(P200).

3. Weineisen M, Schottelius M, Simecek J, Baum RP, Yildiz A, Beykan S, et al. 68Ga- and 177Lu-Labeled PSMA I&T: Optimization of a PSMA-Targeted Theranostic Concept and First Proof-of-Concept Human Studies. J Nucl Med. 2015;56(8):1169-76. Epub 2015/06/20. doi: 10.2967/jnumed.115.158550. PubMed PMID: 26089548.

4. Leggett RW, Williams LR. A proposed blood circulation model for reference man. Health Phys. 1995;69(2):187-201.

5. Buchmann I, Kull T, Glatting G, Bunjes D, Hale G, Kotzerke J, et al. A comparison of the biodistribution and biokinetics of 99mTc-anti-CD66 mAb BW 250/183 and 99mTc-anti-CD45 mAb YTH 24.5 with regard to suitability for myeloablative radioimmunotherapy. Eur J Nucl Med Mol Imaging. 2003;30(5):667-73. PubMed PMID: 12599012.

6. Buckley DL, Roberts C, Parker GJ, Logue JP, Hutchinson CE. Prostate cancer: evaluation of vascular characteristics with dynamic contrast-enhanced T1-weighted MR imaging--initial experience. Radiology. 2004;233(3):709-15. Epub 2004/10/23. doi: 10.1148/radiol.2333032098. PubMed PMID: 15498903.

7. Luczynska E, Heinze-Paluchowska S, Blecharz P, Jereczek-Fossa B, Petralia G, Bellomi M, et al. Correlation between CT perfusion and clinico-pathological features in prostate cancer: a prospective study. Med Sci Monit. 2015;21:153-62. Epub 2015/01/15. doi: 10.12659/MSM.891401. PubMed PMID: 25582437; PubMed Central PMCID: PMC4301468.

8. Franiel T, Lüdemann L, Rudolph B, Rehbein H, Stephan C, Taupitz M, et al. Prostate MR imaging: tissue characterization with pharmacokinetic volume and blood flow parameters and correlation with histologic parameters. Radiology. 2009;252(1):101-8.

9. Wang X, Ma D, Olson WC, Heston WD. In vitro and in vivo responses of advanced prostate tumors to PSMA ADC, an auristatin-conjugated antibody to prostate-specific membrane antigen. Mol Cancer Ther. 2011;10(9):1728-39. Epub 2011/07/14. doi: 10.1158/1535-7163.MCT-11-0191. PubMed PMID: 21750220.

10. Kletting P, Kull T, Maass C, Malik N, Luster M, Beer A, et al. Optimized peptide amount and activity for Y-90-labeled DOTATATE therapy. J Nucl Med. 2015. Epub 2015/12/19. doi: 10.2967/jnumed.115.164699. PubMed PMID: 26678617.

11. Stabin MG, Sparks RB, Crowe E. OLINDA/EXM: The Second-Generation Personal Computer Software for Internal Dose Assessment in Nuclear Medicine. J Nucl Med. 2005;46(6):1023-7.

12. Shah DK, Betts AM. Towards a platform PBPK model to characterize the plasma and tissue disposition of monoclonal antibodies in preclinical species and human. J Pharmacokinet Pharmacodyn. 2012;39(1):67-86. Epub 2011/12/07. doi: 10.1007/s10928-011-9232-2. PubMed PMID: 22143261.

13. Schmidt MM, Wittrup KD. A modeling analysis of the effects of molecular size and binding affinity on tumor targeting. Mol Cancer Ther. 2009;8(10):2861-71. PubMed PMID: 19825804.

14. Fresco GF, DiGiorgio F, Curti GL. Simultaneous estimation of glomerular filtration rate and renal plasma flow. J Nucl Med. 1995;36(9):1701-6. Epub 1995/09/01. PubMed PMID: 7658234.

15. Kletting P, Muller B, Erentok B, Schmaljohann J, Behrendt FF, Reske SN, et al. Differences in predicted and actually absorbed doses in peptide receptor radionuclide therapy. Med Phys. 2012;39(9):5708-17. Epub 2012/09/11. doi: 10.1118/1.4747266. PubMed PMID: 22957636.

16. Groothuis DR. The blood-brain and blood-tumor barriers: a review of strategies for increasing drug delivery. Neuro-oncology. 2000;2(1):45-59. Epub 2001/04/17. PubMed PMID: 11302254; PubMed Central PMCID: PMC1920694.

17. O'Keefe DS, Bacich DJ, Heston WD. Comparative analysis of prostate-specific membrane antigen (PSMA) versus a prostate-specific membrane antigen-like gene. Prostate. 2004;58(2):200-10. Epub 2004/01/13. doi: 10.1002/pros.10319. PubMed PMID: 14716746.

18. Antunes P, Ginj M, Zhang H, Waser B, Baum RP, Reubi JC, et al. Are radiogallium-labelled DOTA-conjugated somatostatin analogues superior to those labelled with other radiometals? Eur J Nucl Med Mol Imaging. 2007;34(7):982-93. PubMed PMID: 17225119.

19. Velikyan I, Sundin A, Eriksson B, Lundqvist H, Sorensen J, Bergstrom M, et al. In vivo binding of [68Ga]-DOTATOC to somatostatin receptors in neuroendocrine tumours--impact of peptide mass. Nucl Med Biol. 2010;37(3):265-75. PubMed PMID: 20346866.

20. Snyder WS, Cook MJ, Nasset ES, Karhausen RS, Howells GP. Report of the Task Group on Reference Man. ICRP publication 23. Oxford: Elsevier; 1975.

21. Berggreen E, Wiig H. Lowering of interstitial fluid pressure in rat submandibular gland: a novel mechanism in saliva secretion. American journal of physiology Heart and circulatory physiology. 2006;290(4):H1460-8. Epub 2005/11/15. doi: 10.1152/ajpheart.00887.2005. PubMed PMID: 16284231.

22. Clough G, Smaje LH. Exchange area and surface properties of the microvasculature of the rabbit submandibular gland following duct ligation. The Journal of physiology. 1984;354:445-56. Epub 1984/09/01. PubMed PMID: 6481642; PubMed Central PMCID: PMC1193423.

23. Ben Jemaa A, Bouraoui Y, Sallami S, Banasr A, Ben Rais N, Ouertani L, et al. Co-expression and impact of prostate specific membrane antigen and prostate specific antigen in prostatic pathologies. J Exp Clin Cancer Res. 2010;29:171. PubMed PMID: 21189143.

**Supporting Information Legends**

Figure A in S1 Text: Classification of compartments and their sub-compartments. (a) PSMA-positive organs, excluding kidney: Blood inflow: Arterial supply for all organs except liver. Liver: Triple inflow sources: arterial, splenic, and gastrointestinal. (b) Kidney: (c) PSMA-negative organs, excluding brain: Blood outflow: Venous drainage for all organs, except spleen and GI tract which drain into the liver. Note: Lung experiences venous inflow and arterial outflow. (d) Brain. Microsoft Office PowerPoint was used to create this figure. Image created using Servier Medical Art (https://smart.servier.com/), licensed under CC BY 4.0.

Table A in S1 Text: All parameters and variables used in PBPK model.
