# Supplementary material for: Multi-scale computational modeling towards efficacy in radiopharmaceutical therapies while minimizing side effects: Modeling of amino acid infusion
Source: PLoS Comput Biol. 2025 Jul 16;21(7):e1013247. doi: 10.1371/journal.pcbi.1013247 (PMC12327665; doi:10.1371/journal.pcbi.1013247)
Supplement: S2 Text — Fig B: The results of fitting the designed PBPK model to data obtained from γ-camera imaging for patient 2. Fig C: The results of fitting the designed PBPK model to data obtained from γ-camera imaging for patient 3. Fig D: The results of fitting the designed PBPK model to data obtained from γ-camera imaging for patient 4. Table A: Parameters that used to calculate absorbed dose for OARs and tumors. Table B: S value for tumors and salivary glands based on their volumes. Table C: Estimated parameters obtained from fitting process for patient 1. Table D: Estimated parameters obtained from fitting process for patient 2. Table E: Estimated parameters obtained from fitting process for patient 3. Table F: Estimated parameters obtained from fitting process for patient 4. (DOCX) [file pcbi.1013247.s002.docx]

Table A in S2 Text: Parameters that used to calculate absorbed dose for OARs and tumors

| **Variable** |  | **Value** | **Unit** | **Source** |
| --- | --- | --- | --- | --- |
| S_K🡨K_ | Dose factor kidneys to kidneys phantom | 4.82·10^-6^ | Gy·min^-1^·MBq^-1^ | [1] |
| S_SAL🡨SAL_ | Dose factor kidneys to kidneys phantom | Table C-F in S2 Text | Gy·min^-1^·MBq^-1^ | [1] |
| S_TU🡨TU_ | Dose factor tumor to tumor phantom | Table C-F in S2 Text | Gy·min^-1^·MBq^-1^ | [2] |
| S_RM🡨RM_ | Dose factor red marrow to red marrow phantom (are scaled using BW) | 7.14·10^-7^ | Gy·min^-1^·MBq^-1^ | [1] |
| S_RM🡨REM_ | Dose factor remainder to red marrow  corrected according to Hindorf et al. | 4.83·10^-9^ | Gy·min^-1^·MBq^-1^ | [1] [3] |
| *A_i_* | activity of organ i |  | MBq |  |
| *A_REM_* | activity of remainder (tumor is part of the remainder) | A_Total_ -A_SAL_-A_K_-A_RM_ | MBq |  |
| *A_inj_* | injected activity |  | MBq |  |
| *ã_i_* | time-integrated activity coefficient of organ i |  | h |  |
| *a_i_* | fraction of administered activity of organ i |  | unity |  |
| *D_i_* | dose to organ i |  | Gy |  |
| *Ḋ_i_* | dose rate to organ i |  | Gy·min^-1^ |  |
| *T* | Integration time | 30000 | min |  |

Table B in S2 Text: S value for tumors and salivary glands based on their volumes

| **Volume [ml]** | **S-values [Gy∙min^-1^∙MBq^-1^]** |
| --- | --- |
| 0.5 | 2.77·10^-3^ |
| 1 | 1.40·10^-3^ |
| 1.5 | 9.00·10^-4^ |
| 2 | 7.02·10^-4^ |
| 3 | 4.50·10^-4^ |
| 4 | 3.52·10^-4^ |
| 13 | 1.10·10^-4^ |
| 17 | 8.30·10^-5^ |
| 21 | 6.90·10^-5^ |
| 29 | 4.80·10^-5^ |
| 34 | 4.30·10^-5^ |
| 52 | 2.67·10^-5^ |
| 54 | 2.65·10^-5^ |

### **Biodistribution Simulations**

The amount of labeled peptides that were taken into consideration for the calculation of absorbed doses for the kidneys, salivary glands, tumor 1, and tumor 2 are computed as follows:

| $P_{k}^{*}=P_{k,v}^{*}+P_{k,int}^{*}+RP_{k}^{*}+P_{k,intern}^{*}+PRP^{*}.\left( \frac{V_{k,v}}{V_{p}+V_{tumor 1,v}+V_{tumor 2,v}+V_{tumor rest,v}} \right)$ | (S14) |
| --- | --- |
| $P_{SG}^{*}=P_{SG,v}^{*}+P_{SG,int}^{*}+RP_{SG}^{*}+P_{SG,intern}^{*}+PRP^{*}.\left( \frac{V_{SG,v}}{V_{p}+V_{tumor 1,v}+V_{tumor 2,v}+V_{tumor rest,v}} \right)$ | (S15) |
| $P_{tu1}^{*}=P_{tu1,v}^{*}+P_{tu1,int}^{*}+RP_{tu1}^{*}+P_{tu1,intern}^{*}+PRP^{*}.\left( \frac{V_{tu1,v}}{V_{p}+V_{tumor 1,v}+V_{tumor 2,v}+V_{tumor rest,v}} \right)+c_{1}.\left( P_{mus,v}^{*}+P_{mus,int}^{*}+PRP^{*}.\left( \frac{V_{mus,v}}{V_{p}+V_{tumor 1,v}+V_{tumor 2,v}+V_{tumor rest,v}} \right) \right)$ | (S16) |
| $P_{tu2}^{*}=P_{tu2,v}^{*}+P_{tu2,int}^{*}+RP_{tu2}^{*}+P_{tu2,intern}^{*}+PRP^{*}.\left( \frac{V_{tu2,v}}{V_{p}+V_{tumor 1,v}+V_{tumor 2,v}+V_{tumor rest,v}} \right)+c_{2}.\left( P_{mus,v}^{*}+P_{mus,int}^{*}+PRP^{*}.\left( \frac{V_{mus,v}}{V_{p}+V_{tumor 1,v}+V_{tumor 2,v}+V_{tumor rest,v}} \right) \right)$ | (S17) |

It should be noted that the calculated AD is an estimate and the actual AD is likely to be higher, taking into account the integration of all metastatic lesions into the tumor residual compartment and the decrease in the "S" value with an increase in tumor volume.

**Parameter Estimation**

Since the $\gamma$-camera images obtained from patients during therapy are not accessible, we extracted 5 data points resulted from diagrams provided by Kletting et al. (Fig. 5) using the GetData Graph Digitizer software (available at getdata-graph-digitizer.com). The individual parameters were estimated by fitting our PBPK model to the extracted data via nonlinear regression and nonlinear least squares optimization to personalize the model. The method is based on minimizing sum of squares of the residuals between the model and the data using the Levenberg-Marquardt algorithm (Simbiology Model Analyzer). The difference between the fitted time activity curve TAC and the data points acquired from γ-camera imaging was assessed using Mean Squared Error (MSE), Sum of Squared Errors (SSE), Akaike Information Criterion (AIC), and Bayesian Information Criterion (BIC), calculated using equations S18-S21. MSE and SSE assess the proximity of model predictions to observed data, whereas AIC and BIC strike a balance between model fit and complexity, facilitating model selection. AIC and BIC penalize models with more parameters to prevent overfitting. Log-likelihood evaluates the likelihood of observed data given the model parameters, essential for parameter estimation. These metrics collectively inform the processes of model fitting, selection, and parameter estimation in Simbiology.

| $MSE= \frac{1}{N}\sum_{i}^{N} \left( y_{i}-\hat{y}_{i} \right)^{2}$ | (S18) |
| --- | --- |
| $SSE= \sum_{i}^{N} \left( y_{i}-\hat{y}_{i} \right)^{2}$ | (S19) |

Where N is number of measured data on Time activity curve, $y_{i}$ is estimated value, and $\hat{y}_{i}$ is acquired value from $\gamma$-camera.

| $AIC = N \times log\left( det\left( \frac{1}{N}\sum_{i}^{N} \varepsilon\left( t,\hat{\theta}_{N} \right)\left( \varepsilon\left( t,\hat{\theta}_{N} \right) \right)^{T} \right) \right)+2n_{p}+N\times\left( n_{y}\times\left( log\left( 2\pi\right)+1 \right) \right)$ | (S20) |
| --- | --- |
| $BIC = N \times log\left( det\left( \frac{1}{N}\sum_{i}^{N} \varepsilon\left( t,\hat{\theta}_{N} \right)\left( \varepsilon\left( t,\hat{\theta}_{N} \right) \right)^{T} \right) \right)+N\times\left( n_{y}\times\left( log\left( 2\pi\right)+1 \right) \right)+n_{p}\times log\left( N \right)$ | (S21) |

Where $N$ is the number of values in the estimation dataset, $\varepsilon(t)$ is a $ny$ $by$ $1$ vector of prediction errors, $\hat{\theta}_{N}$ represents the estimated parameters $n_{p}$ is the number of estimated parameters, and $n_{y}$ is the number of model outputs.

| 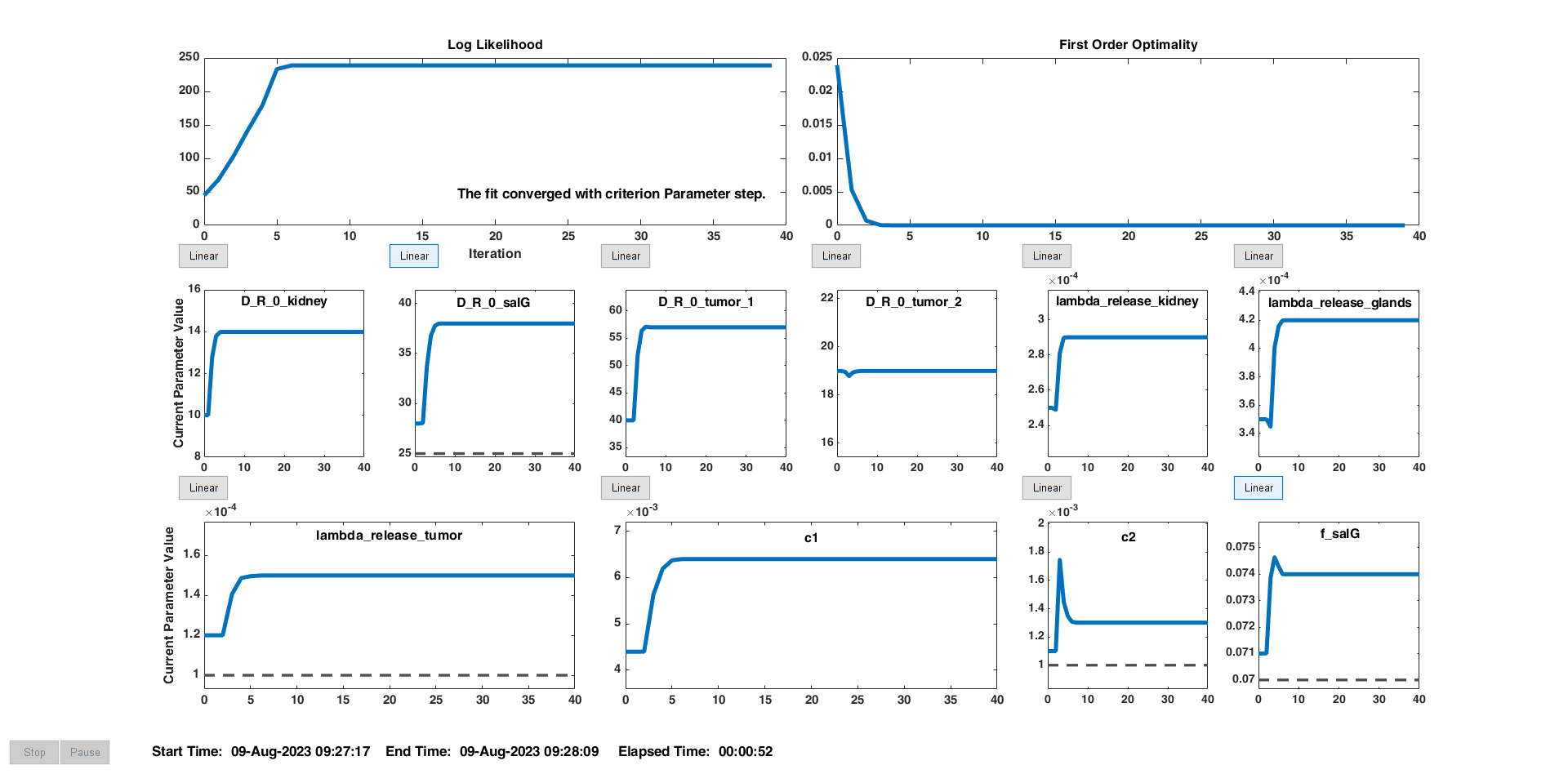  Figure A in S2 Text: The results of fitting the designed PBPK model to data obtained from $\gamma$-camera imaging for patient 1. |
| --- |
| Table C in S2 Text: Estimated parameters obtained from fitting process for patient 1.   \| **Estimated parameter** \| **'D_R_0_kidney'** \| **'D_R_0_salG'** \| **'D_R_0_tumor1'** \| **'D_R_0_tumor2'** \| **'lambda**  **Release**  **kidney'** \| **'lambda**  **Release**  **glands'** \| **'lambda**  **Release**  **tumor'** \| **'c1'** \| **'c2'** \| **'f_salG'** \| \| --- \| --- \| --- \| --- \| --- \| --- \| --- \| --- \| --- \| --- \| --- \| \| **Patient 2** \| **14** \| **38** \| **57** \| **19** \| **0.00029** \| **0.00042** \| **0.00015** \| **0.0064** \| **0.0013** \| **0.074** \| \| **Standard Error** \| **8.17E-5** \| **0.0038** \| **0.032** \| **0.0016** \| **8.57E-09** \| **9.18E-08** \| **5.15E-08** \| **1.87E-06** \| **3.61E-06** \| **7.7E-06** \| \| **LogLikelihood** \| \| \| \| \| **238.8** \| \| \| \| \| \| \| **MSE** \| \| \| \| \| **2.1E-11** \| \| \| \| \| \| \| **SSE** \| \| \| \| \| **4.96.30E-11** \| \| \| \| \| \| \| **AIC** \| \| \| \| \| **-457** \| \| \| \| \| \| \| **BIC** \| \| \| \| \| **-447** \| \| \| \| \| \| \| **Iterations** \| \| \| \| \| **39** \| \| \| \| \| \| |

| 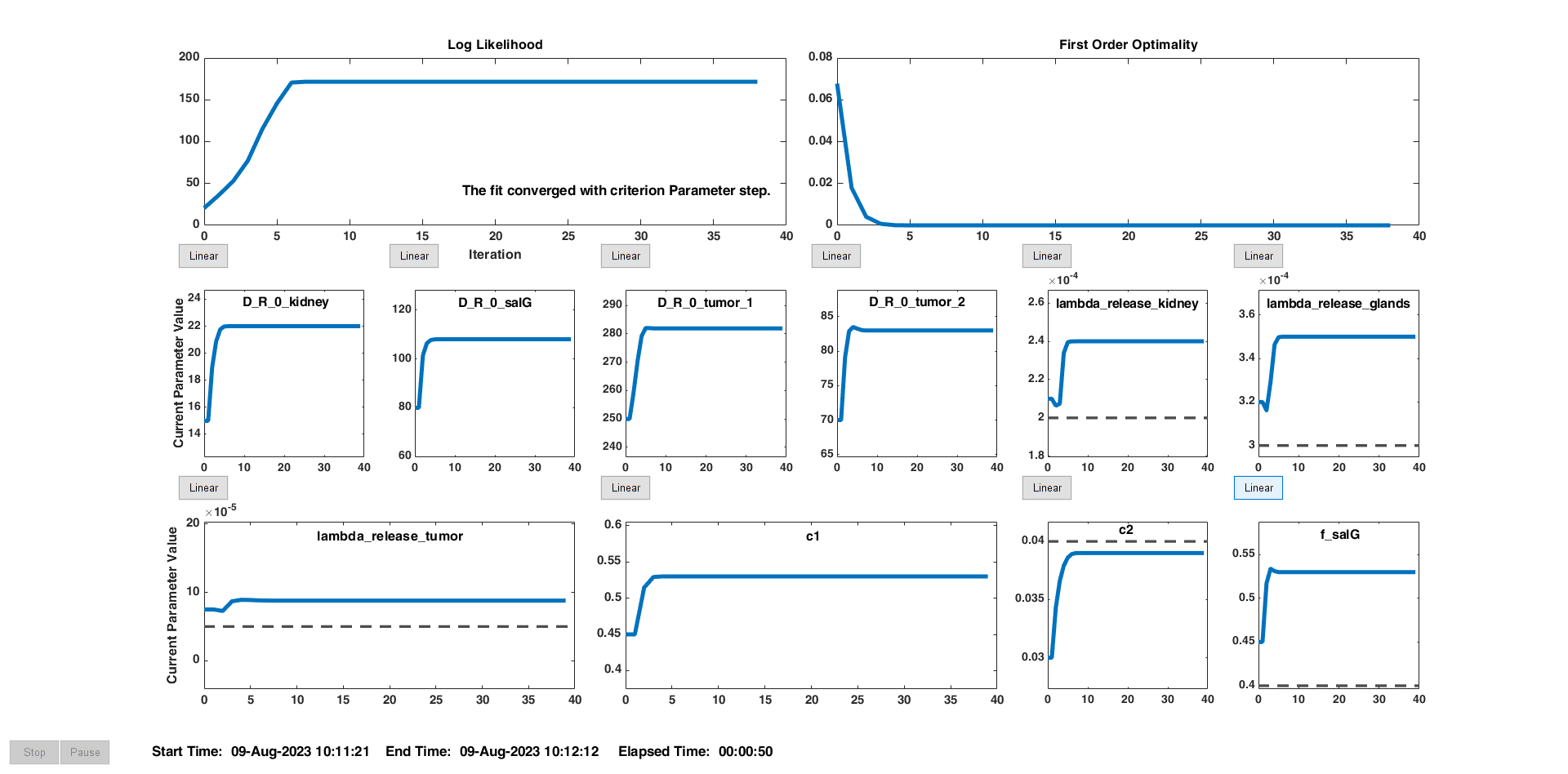  Figure B in S2 Text: The results of fitting the designed PBPK model to data obtained from $\gamma$-camera imaging for patient 2. |
| --- |
| Table D in S2 Text: Estimated parameters obtained from fitting process for patient 2.   \| **Estimated parameter** \| **'D_R_0_kidney'** \| **'D_R_0_salG'** \| **'D_R_0_tumor1'** \| **'D_R_0_tumor2'** \| **'lambda**  **Release**  **kidney'** \| **'lambda**  **Release**  **glands'** \| **'lambda**  **Release**  **tumor'** \| **'c1'** \| **'c2'** \| **'f_salG'** \| \| --- \| --- \| --- \| --- \| --- \| --- \| --- \| --- \| --- \| --- \| --- \| \| **Patient 3** \| **22** \| **108** \| **282** \| **83** \| **0.00024** \| **0.00035** \| **0.000088** \| **0.53** \| **0.039** \| **0.53** \| \| **Standard Error** \| **0.00027** \| **0.0083** \| **0.138** \| **0.011** \| **2.03E-08** \| **1.43E-07** \| **8.0E-08** \| **6.39E-06** \| **7.22E-06** \| **9.85E-05** \| \| **LogLikelihood** \| \| \| \| \| **171.6** \| \| \| \| \| \| \| **MSE** \| \| \| \| \| **7.51E-11** \| \| \| \| \| \| \| **SSE** \| \| \| \| \| **4.50E-10** \| \| \| \| \| \| \| **AIC** \| \| \| \| \| **-323** \| \| \| \| \| \| \| **BIC** \| \| \| \| \| **-315** \| \| \| \| \| \| \| **Iterations** \| \| \| \| \| **38** \| \| \| \| \| \| |

| 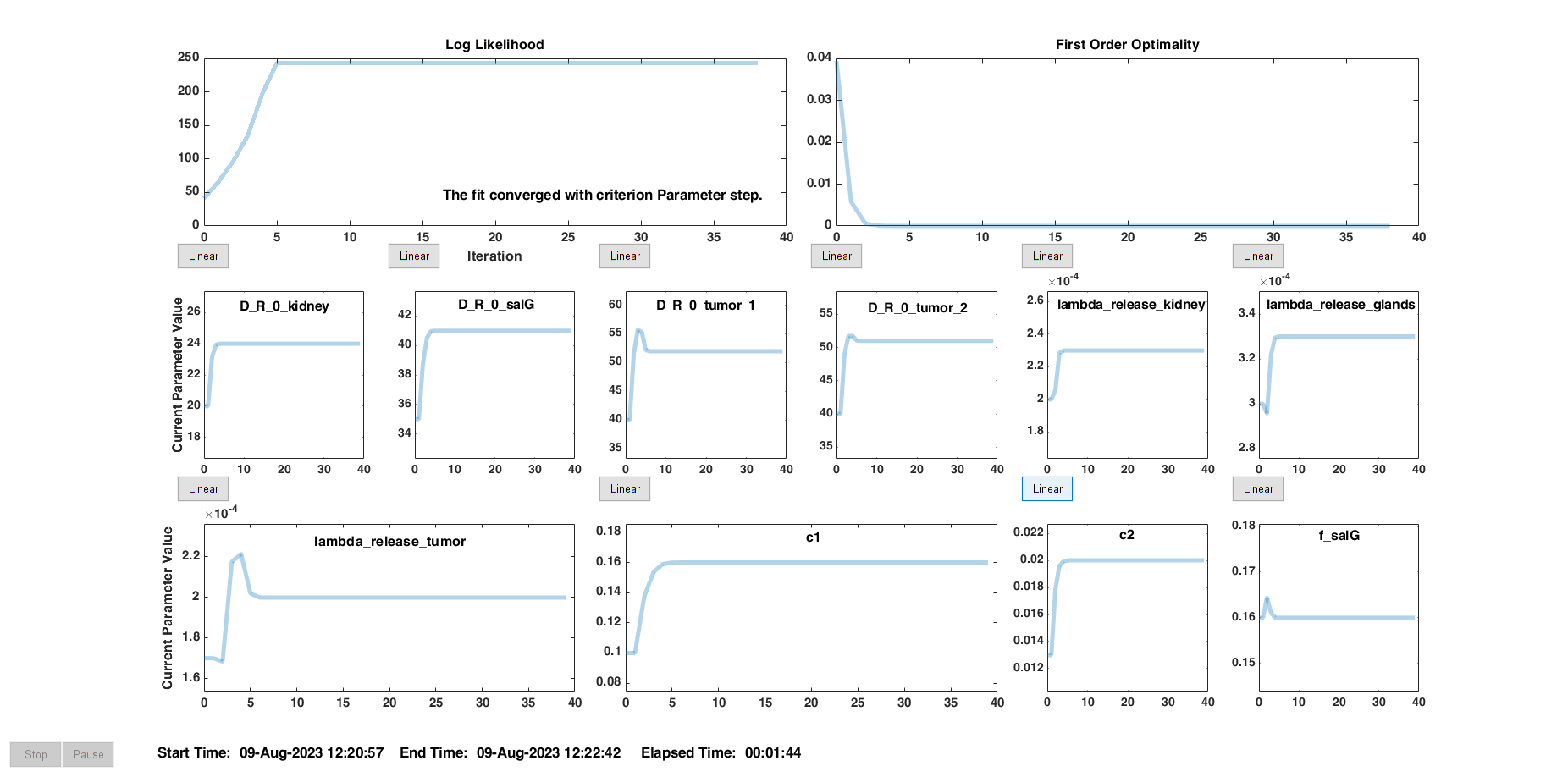  Figure C in S2 Text: The results of fitting the designed PBPK model to data obtained from $\gamma$-camera imaging for patient 3. |
| --- |
| Table E in S2 Text: Estimated parameters obtained from fitting process for patient 3.   \| **Estimated parameter** \| **'D_R_0_kidney'** \| **'D_R_0_salG'** \| **'D_R_0_tumor1'** \| **'D_R_0_tumor2'** \| **'lambda**  **Release**  **kidney'** \| **'lambda**  **Release**  **glands'** \| **'lambda**  **Release**  **tumor'** \| **'c1'** \| **'c2'** \| **'f_salG'** \| \| --- \| --- \| --- \| --- \| --- \| --- \| --- \| --- \| --- \| --- \| --- \| \| **Patient 4** \| **24** \| **41** \| **52** \| **51** \| **0.00023** \| **0.00033** \| **0.0002** \| **0.16** \| **0.02** \| **0.16** \| \| **Standard Error** \| **7.43E-5** \| **0.00068** \| **0.0068** \| **0.0085** \| **3.88E-09** \| **1.81E-8** \| **9.23E-8** \| **1.51E-6** \| **1.5E-6** \| **4.67E-6** \| \| **LogLikelihood** \| \| \| \| \| **243.3** \| \| \| \| \| \| \| **MSE** \| \| \| \| \| **3.19E-12** \| \| \| \| \| \| \| **SSE** \| \| \| \| \| **3.19E-11** \| \| \| \| \| \| \| **AIC** \| \| \| \| \| **-466** \| \| \| \| \| \| \| **BIC** \| \| \| \| \| **-456** \| \| \| \| \| \| \| **Iterations** \| \| \| \| \| **38** \| \| \| \| \| \| |

| 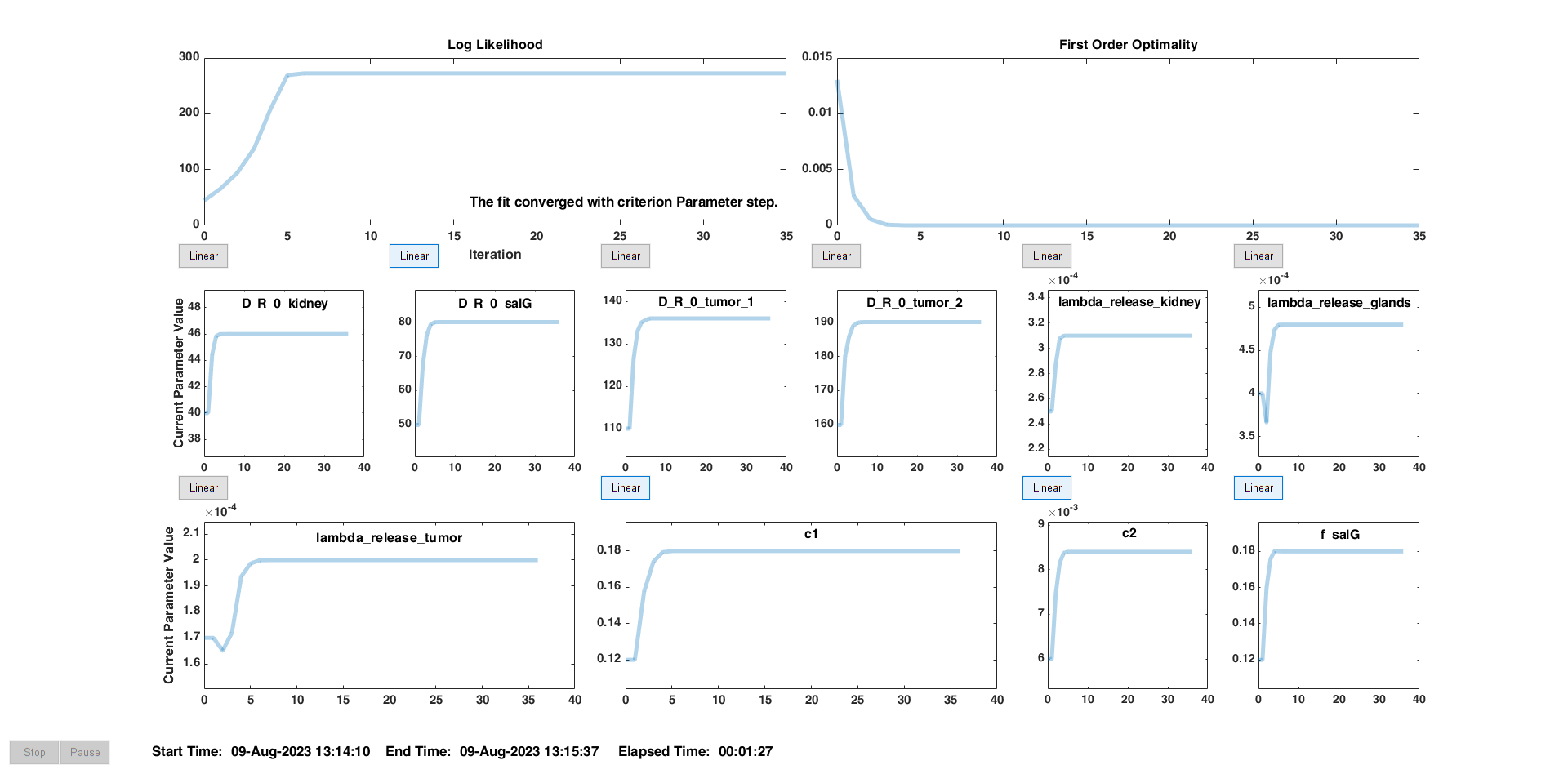  Figure D in S2 Text: The results of fitting the designed PBPK model to data obtained from $\gamma$-camera imaging for patient 4. |
| --- |
| Table F in S2 Text: Estimated parameters obtained from fitting process for patient 4.   \| **Estimated parameter** \| **'D_R_0_kidney'** \| **'D_R_0_salG'** \| **'D_R_0_tumor1'** \| **'D_R_0_tumor2'** \| **'lambda**  **Release**  **kidney'** \| **'lambda**  **Release**  **glands'** \| **'lambda**  **Release**  **tumor'** \| **'c1'** \| **'c2'** \| **'f_salG'** \| \| --- \| --- \| --- \| --- \| --- \| --- \| --- \| --- \| --- \| --- \| --- \| \| **Patient 5** \| **46** \| **80** \| **136** \| **190** \| **0.00031** \| **0.00048** \| **0.0002** \| **0.18** \| **0.0084** \| **0.18** \| \| **Standard Error** \| **3.81E-5** \| **5.16E-4** \| **0.0096** \| **0.013** \| **1.04E-9** \| **9.57E-9** \| **4.11E-8** \| **3.47E-7** \| **3.06E-6** \| **3.81E-6** \| \| **LogLikelihood** \| \| \| \| \| **272.4** \| \| \| \| \| \| \| **MSE** \| \| \| \| \| **1.72E-13** \| \| \| \| \| \| \| **SSE** \| \| \| \| \| **1.72E-12** \| \| \| \| \| \| \| **AIC** \| \| \| \| \| **-524** \| \| \| \| \| \| \| **BIC** \| \| \| \| \| **-514** \| \| \| \| \| \| \| **Iterations** \| \| \| \| \| **35** \| \| \| \| \| \| |

**References**

1. Stabin MG, Sparks RB, Crowe E. OLINDA/EXM: The Second-Generation Personal Computer Software for Internal Dose Assessment in Nuclear Medicine. J Nucl Med. 2005;46(6):1023-7.

2. Stabin MG, Siegel JA. Physical models and dose factors for use in internal dose assessment. Health Phys. 2003;85(3):294-310. PubMed PMID: 12938720.

3. Hindorf C, Glatting G, Chiesa C, Lindén O, Flux G. EANM Dosimetry Committee guidelines for bone marrow and whole-body dosimetry. Eur J Nucl Med Mol Imaging. 2010;37(6):1238-50.

**Supporting Information Legends**

Figure A in S2 Text: The results of fitting the designed PBPK model to data obtained from $\gamma$-camera imaging for patient 1.

Figure B in S2 Text: The results of fitting the designed PBPK model to data obtained from $\gamma$-camera imaging for patient 2.

Figure C in S2 Text: The results of fitting the designed PBPK model to data obtained from $\gamma$-camera imaging for patient 3.

Figure D in S2 Text: The results of fitting the designed PBPK model to data obtained from $\gamma$-camera imaging for patient 4.

Table A in S2 Text: Parameters that used to calculate absorbed dose for OARs and tumors

Table B in S2 Text: S value for tumors and salivary glands based on their volumes

Table C in S2 Text: Estimated parameters obtained from fitting process for patient 1.

Table D in S2 Text: Estimated parameters obtained from fitting process for patient 2.

Table E in S2 Text: Estimated parameters obtained from fitting process for patient 3.

Table F in S2 Text: Estimated parameters obtained from fitting process for patient 4.
