# Supplementary material for: Multi-scale computational modeling towards efficacy in radiopharmaceutical therapies while minimizing side effects: Modeling of amino acid infusion
Source: PLoS Comput Biol. 2025 Jul 16;21(7):e1013247. doi: 10.1371/journal.pcbi.1013247 (PMC12327665; doi:10.1371/journal.pcbi.1013247)
Supplement: S3 Text — The parameters—tumor serum flow density (f_tumor), permeability surface area product per unit mass (k_tumor), association rate (k_on), dissociation rate (k_off), internalization rate (lambda_int), physical decay (lambda_phy), PSMA binding site density (RD), and release rate from tumor cells (lambda_release)—were varied across four orders of magnitude (100, 10, 0.1, and 0.01). The mean and median of relative errors are displayed by red dash line and black solid line, respectively. Table A: The parameters used for computational results of the mathematical model. Table B: Absorbed dose due to presence of radiopharmaceuticals in tumor interstitial space, tumor cell receptors, and internalized within tumor cells) for four patients. (DOCX) [file pcbi.1013247.s003.docx]

**Angiogenesis**

**Computational Method for Modeling Angiogenesis**

We chose a computational area of 1×1 cm², structured into a grid of 400×400 nodes. The angiogenesis equations were made dimensionless, leading to a reformulated set of governing equations within a unit square domain [0,1]×[0,1]. These equations were then converted into a series of algebraic expressions through a finite difference method, as detailed in Equations S22-24. The analysis involves using stochastic discretized equations tailored for the study of tip endothelial cells (tEC). The graphical representation of the probability distribution for tEC movement around a central node is depicted in Fig. 1. Equations S25-29 specify the probabilities for tECs to either stay in place or move in one of four directions: right, left, up, or down. The uniform grid size in both x and y dimensions is denoted by h, and q represents the time increment. Parameters for the angiogenesis model were deduced from standard values for TAF ($c_{0}$) and fibronectin ($f_{0}$), EC density ($n_{0}$), length ($L$) and time ($\tau$) and are listed in Table S5. Non-dimensional parameters were introduced as $\tilde{c}=\frac{c}{c_{0}}$, $\tilde{f}=\frac{f}{f_{0}}$, $\tilde{n}=\frac{n}{n_{0}}$, and $\tilde{t}=\frac{t}{\tau}$ where $\tau=\frac{L^{2}}{D_{c}}$ ($D_{c}$ is the TAF diffusion coefficient). For clarity, the tilde ($\sim$) of the parameters is dropped. Solving discretized equations is done in an iterative process. Starting with the initial values for *n*, *f*, and $c$, a new value is obtained at each time step. This value is used as an updated value for the next step and the solution continues until a specified time. The process of solving the discretized equations involves iteration: starting with initial conditions for *n*, *f*, and $c$, each time step produces a new set of values which then serve as the basis for the next calculation, proceeding until reaching a predetermined time.

| $n_{i,j}^{q+1}=n_{i,j}^{q}P_{0}+n_{i+1,j}^{q}P_{1}+n_{i-1,j}^{q}P_{2}+n_{i,j+1}^{q}P_{3}+n_{i,j-1}^{q}P_{4}$ | (S22) |
| --- | --- |
| $f_{i,j}^{q+1}=f_{i,j}^{q}[1-\Delta t\gamma n_{i,j}^{q}]+\Delta t\beta n_{i,j}^{q}$ | (S23) |
| $c_{i,j}^{q+1}=c_{i,j}^{q}[1-\Delta t\eta n_{i,j}^{q}]$ | (S24) |

**Migration probabilities for a central tEC:**

| $P_{0}=1-\frac{4\Delta tD_{n}}{h^{2}}+[\frac{\Delta t\chi\left( 1+\alpha c_{i,j}^{q} \right)}{h^{2}}\left( c_{i+1,j}^{q}+c_{i-1,j}^{q}-4c_{i,j}^{q}+c_{i,j+1}^{q}+c_{i,j-1}^{q} \right)-\frac{\Delta t\phi}{h^{2}}\left( f_{i+1,j}^{q}+f_{i-1,j}^{q}-4f_{i,j}^{q}+f_{i,j+1}^{q}+f_{i,j-1}^{q} \right)]$ | (S25) |
| --- | --- |
| $P_{1}=\frac{\Delta tD_{n}}{h^{2}}-\frac{\Delta t}{4h^{2}}\left[ \chi\left( 1+\alpha c_{i+1,j}^{q} \right)\left( c_{i+1,j}^{q}-c_{i-1,j}^{q} \right)+\phi\left( f_{i+1,j}^{q}-f_{i-1,j}^{q} \right) \right]$ | (S26) |
| $P_{2}=\frac{\Delta tD_{n}}{h^{2}}+\frac{\Delta t}{4h^{2}}\left[ \chi\left( 1+\alpha c_{i-1,j}^{q} \right)\left( c_{i+1,j}^{q}-c_{i-1,j}^{q} \right)+\phi\left( f_{i+1,j}^{q}-f_{i-1,j}^{q} \right) \right]$ | (S27) |
| $P_{3}=\frac{\Delta tD_{n}}{h^{2}}-\frac{\Delta t}{4h^{2}}\left[ \chi\left( 1+\alpha c_{i,j+1}^{q} \right)\left( c_{i,j+1}^{q}-c_{i,j-1}^{q} \right)+\phi\left( f_{i,j+1}^{q}-f_{i,j-1}^{q} \right) \right]$ | (S28) |
| $P_{4}=\frac{\Delta tD_{n}}{h^{2}}+\frac{\Delta t}{4h^{2}}\left[ \chi\left( 1+\alpha c_{i,j-1}^{q} \right)\left( c_{i,j+1}^{q}-c_{i,j-1}^{q} \right)+\phi\left( f_{i,j+1}^{q}-f_{i,j-1}^{q} \right) \right]$ | (S29) |

Table A in S3 Text. The parameters used for computational results of the mathematical model.

| **Parameters** | **Description** | **Value** | **References** |
| --- | --- | --- | --- |
| $D_{n}$ | Random motility coefficient of EC | 3.5×10^-4^ ${cm}^{2}/s$ | [1] |
| $\chi$ | Chemotaxis coefficient | 0.16 [unity] | [1] |
| $\alpha$ | Constant of chemotaxis coefficient | 0.6 [unity] | [1, 2] |
| $\phi$ | Haptotaxis coefficient | 0.34 [unity] | [1] |
| $\beta$ | Production coefficient of fibronectin | 0.01 [unity] | [1] |
| $\gamma$ | Uptake coefficient of fibronectin | 0.1 [unity] | [1] |
| $\eta$ | Uptake coefficient of TAF | 0.1 [unity] | [1] |
| $c_{0}$ | Reference value for concentration of TAF | 1.1×10^-8^ M | [1, 3] |
| $f_{0}$ | Reference value for concentration of fibronectin | 1.36×10^-9^ M | [1] |
| $n_{0}$ | Reference value for concentration of EC density | 10^-10^ M | [1, 4] |
| $D_{c}$ | TAF diffusion coefficient | 2.9×10^-7^ ${cm}^{2}/s$ | [1] |

| Table B in S3 Text. Absorbed dose due to presence of radiopharmaceuticals in tumor interstitial space, tumor cell receptors, and internalized within tumor cells) for four patients. |
| --- |
| \| **Patients** \| **PBPK** \| **CDR** \| **RE%** \| **PBPK** \| **CDR** \| **RE%** \| **PBPK** \| **CDR** \| **RE%** \| \| --- \| --- \| --- \| --- \| --- \| --- \| --- \| --- \| --- \| --- \| \| **int** \| **int** \| **int** \| **bound** \| **bound** \| **bound** \| **intern** \| **intern** \| **intern** \| \| **1** \| 0.04 \| 0.03 \| 14.29 \| 2.5 \| 2.4 \| 4 \| 11.7 \| 9.9 \| 15.38 \| \| **2** \| 0.02 \| 0.02 \| 4.74 \| 11.54 \| 10.93 \| 5.29 \| 71.64 \| 65 \| 9.27 \| \| **3** \| 0.04 \| 0.04 \| 5.24 \| 2.77 \| 2.73 \| 1.58 \| 10.21 \| 9.3 \| 8.91 \| \| **4** \| 0.04 \| 0.04 \| 4 \| 3.4 \| 3.32 \| 2.38 \| 12.52 \| 11.17 \| 10.78 \| \| **average** \| 0.03 \| 0.03 \| 7.07 \| 5.05 \| 4.84 \| 3.31 \| 26.52 \| 23.84 \| 11.09 \| \| **std** \| 0.01 \| 0.01 \| 4.19 \| 3.76 \| 3.53 \| 1.44 \| 26.07 \| 23.77 \| 2.58 \| \| **min** \| 0.02 \| 0.02 \| 4 \| 2.5 \| 2.4 \| 1.58 \| 10.21 \| 9.3 \| 8.91 \| \| **max** \| 0.04 \| 0.04 \| 14.29 \| 11.54 \| 10.93 \| 5.29 \| 71.64 \| 65 \| 15.38 \| \| **P-Value** \| **0.0004 < 0.05** \| \| \| \| \| \| \| \| \| |

| 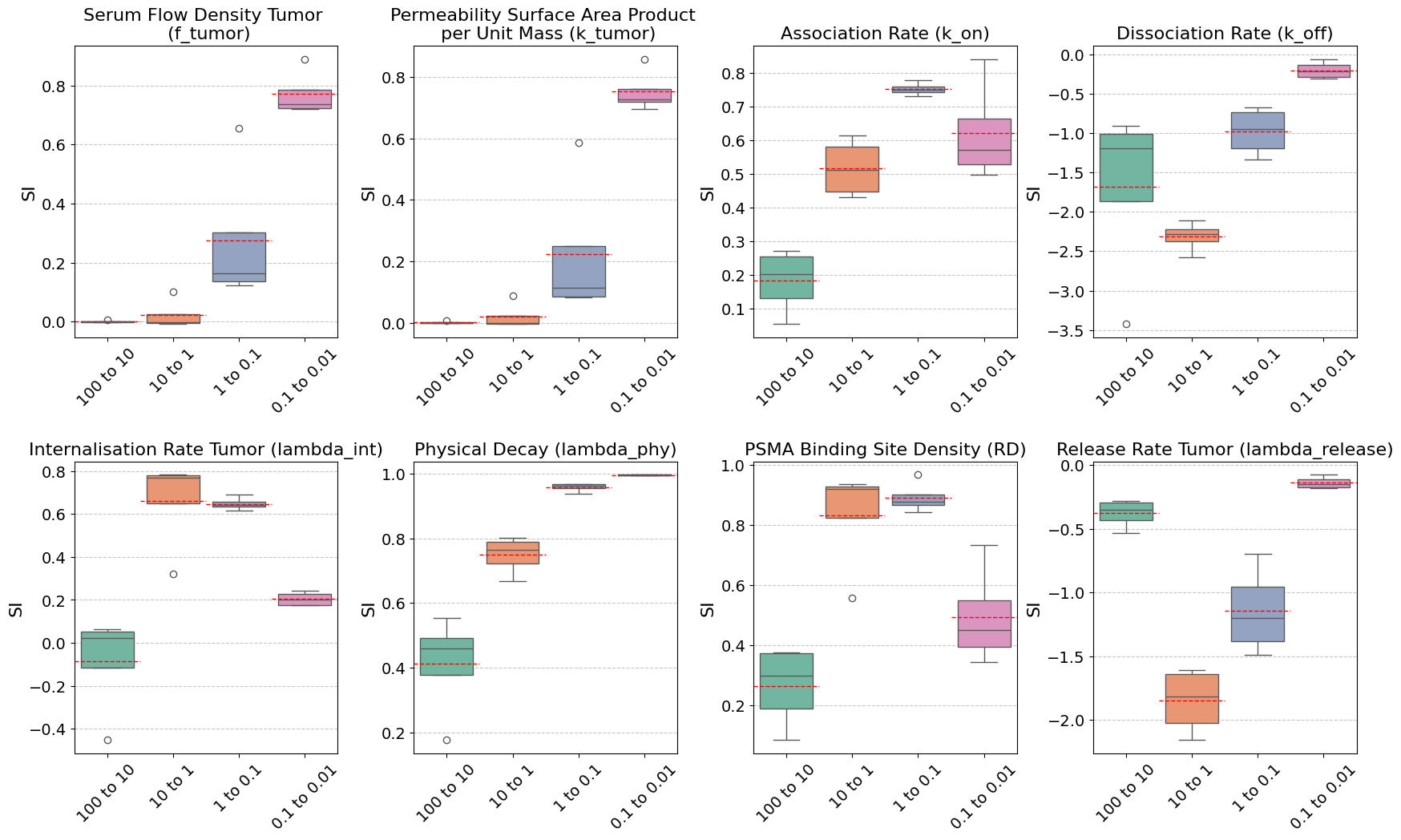 |
| --- |
| Figure A in S3 Text: Box plot showing the Normalized Sensitivity Indices (SI) Across Parameters and Scaling Ranges. The parameters—tumor serum flow density (f_tumor), permeability surface area product per unit mass (k_tumor), association rate (k_on), dissociation rate (k_off), internalization rate (lambda_int), physical decay (lambda_phy), PSMA binding site density (RD), and release rate from tumor cells (lambda_release)—were varied across four orders of magnitude (100, 10, 0.1, and 0.01). The mean and median of relative errors are displayed by red dash line and black solid line, respectively. |

**References**

1. Anderson AR, Chaplain MAJ. Continuous and discrete mathematical models of tumor-induced angiogenesis. Bull Math Biol. 1998;60:857–899.

2. Stéphanou A, McDougall SR, Anderson AR, Chaplain MA. Mathematical modelling of the influence of blood rheological properties upon adaptative tumour-induced angiogenesis. Math Comput Model. 2006;44:96–123.

3. Tang L, Van De Ven AL, Guo D, Andasari V, Cristini V, Li KC, et al. Computational modeling of 3D tumor growth and angiogenesis for chemotherapy evaluation. PLoS One. 2014;9:e83962.

4. Plank M, Sleeman B, Jones P. A mathematical model of tumour angiogenesis, regulated by vascular endothelial growth factor and the angiopoietins. J Theor Biol. 2004;229:435–454.

**Supporting Information Legends**

Figure A in S3 Text: Box plot showing the Normalized Sensitivity Indices (SI) Across Parameters and Scaling Ranges. The parameters—tumor serum flow density (f_tumor), permeability surface area product per unit mass (k_tumor), association rate (k_on), dissociation rate (k_off), internalization rate (lambda_int), physical decay (lambda_phy), PSMA binding site density (RD), and release rate from tumor cells (lambda_release)—were varied across four orders of magnitude (100, 10, 0.1, and 0.01). The mean and median of relative errors are displayed by red dash line and black solid line, respectively.

Table A in S3 Text. The parameters used for computational results of the mathematical model.

Table B in S3 Text. Absorbed dose due to presence of radiopharmaceuticals in tumor interstitial space, tumor cell receptors, and internalized within tumor cells) for four patients.
